# Intrinsic Superprotonic Conduction in Metal‐Free Hydrogen‐Bonded Organic–Inorganic Frameworks

**DOI:** 10.1002/advs.77910

**Published:** 2026-09-27

**Authors:** Jin Chen, Yongqiang Cheng, Simon J. Teat, Gobin Raj Acharya, Roknuzzaman Roknuzzaman, Wensong Yu, Yucong Xia, Weston C. Swallow, Xiaoying Huang, Xiaodong Yang, Thomas J. Emge, Kui Tan, Andrew J. Nieuwkoop, Lawrence J. Williams, Jing Li

**Affiliations:** ^1^ Department of Chemistry and Chemical Biology Rutgers University Piscataway New Jersey USA; ^2^ Neutron Scattering Division Oak Ridge National Laboratory Oak Ridge Tennessee USA; ^3^ Advanced Light Source Lawrence Berkeley National Laboratory Berkeley California USA; ^4^ Department of Chemistry University of North Texas Denton Texas USA; ^5^ College of Materials Science and Engineering Huaqiao University Xiamen China; ^6^ State Key Laboratory of Structural Chemistry Fujian Institute of Research on the Structure of Matter The Chinese Academy of Sciences Fuzhou Fujian China

**Keywords:** crystal, hybrid frameworks, hydrogen bonds, hydrogen‐bond networks, metal‐free, proton conductivity

## Abstract

Crystalline hybrid networks containing metal coordination centers often disrupt continuous hydrogen‐bonding pathways, limiting their intrinsic proton conductivity. Here we report a suite of hydrogen‐bonded organic‐inorganic frameworks (HOIFs) composed of ionic/neutral organic and metal‐free inorganic building units that assemble into two‐ and three‐dimensional hydrogen bonded networks through dense, multidirectional hydrogen bonds. These materials are nonporous and can be classified into three categories based on charge characteristics of their organic and inorganic components. They exhibit a rich structural diversity and unprecedented topologies such as the ***hoi*** net in HOIF‐101. Systematic investigations show that they possess intrinsic superprotonic conductivity, with most of them achieving conductivity values >10^−^^2^ S·cm^−^^1^ at 85°C and 100% RH, outperforming their organic counterparts by one to several orders of magnitude and exceeding those of most reported MOFs as well as other metal‐free porous frameworks. Comprehensive spectroscopic and computational studies reveal that dynamic inorganic motifs strengthen and modulate extended hydrogen‐bond networks and can act as molecular “pumps” to promote Grötthuss‐type proton hopping in HOIF‐101. Metal‐free hybridization thus provides a unique, simple and versatile route to engineer proton mobility and enhance conductivity, expanding the design space for high‐performance solid proton conductors with strong potential for diverse applications.

## Graphical abstract

A simple hybridization strategy yields a suite of metal‐free hydrogen‐bonded organic‐inorganic frameworks (HOIFs) with dense, uninterrupted hydrogen‐bonded networks that possess intrinsically high superprotonic conductivity, establishing a versatile and scalable platform for high‐performance solid‐state proton conductors.

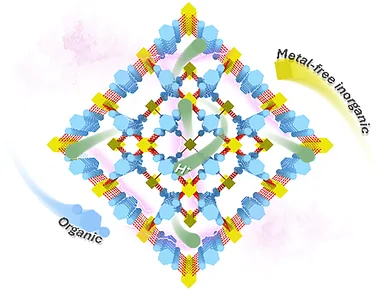

## 1 Introduction

Proton conduction is fundamental to energy transduction in both living and synthetic systems, driving processes from cellular respiration to electrochemical conversion [1, 2]. It governs the performance of fuel cells and batteries [3, 4, 5] while enabling innovations in environmental sensors [6, 7] and iontronics, including neuromorphic memristors for artificial intelligence [8, 9, 10]. The intrinsic compatibility of proton conductors with flexible and printable formats further positions them at the frontier of wearable electronics and soft robotics [11, 12].

Hydrogen bonding (H‐bonding) is widely recognized as a natural promoter of proton conduction. Extended H‐bonding networks are particularly effective in enhancing transport efficiency in crystalline frameworks [13, 14]. Hydrogen‐bonded organic frameworks (HOFs), constructed solely from organic molecules, inherently possess such networks and exhibit respectable proton conductivity (σ) [15, 16, 17, 18, 19]. Likewise, ionic HOFs (iHOFs) based on pure organic acid‐base pairs extend this family while involving charge‐assisted H‐bonds [20, 21]. However, the bulky organic units and large porosity in these systems constrain the H‐bond density/continuity. Compact inorganic modules are absent or, if present at all, have received scant attention compared to organic building blocks in constructing H‐bonded frameworks.

**FIGURE 1:**
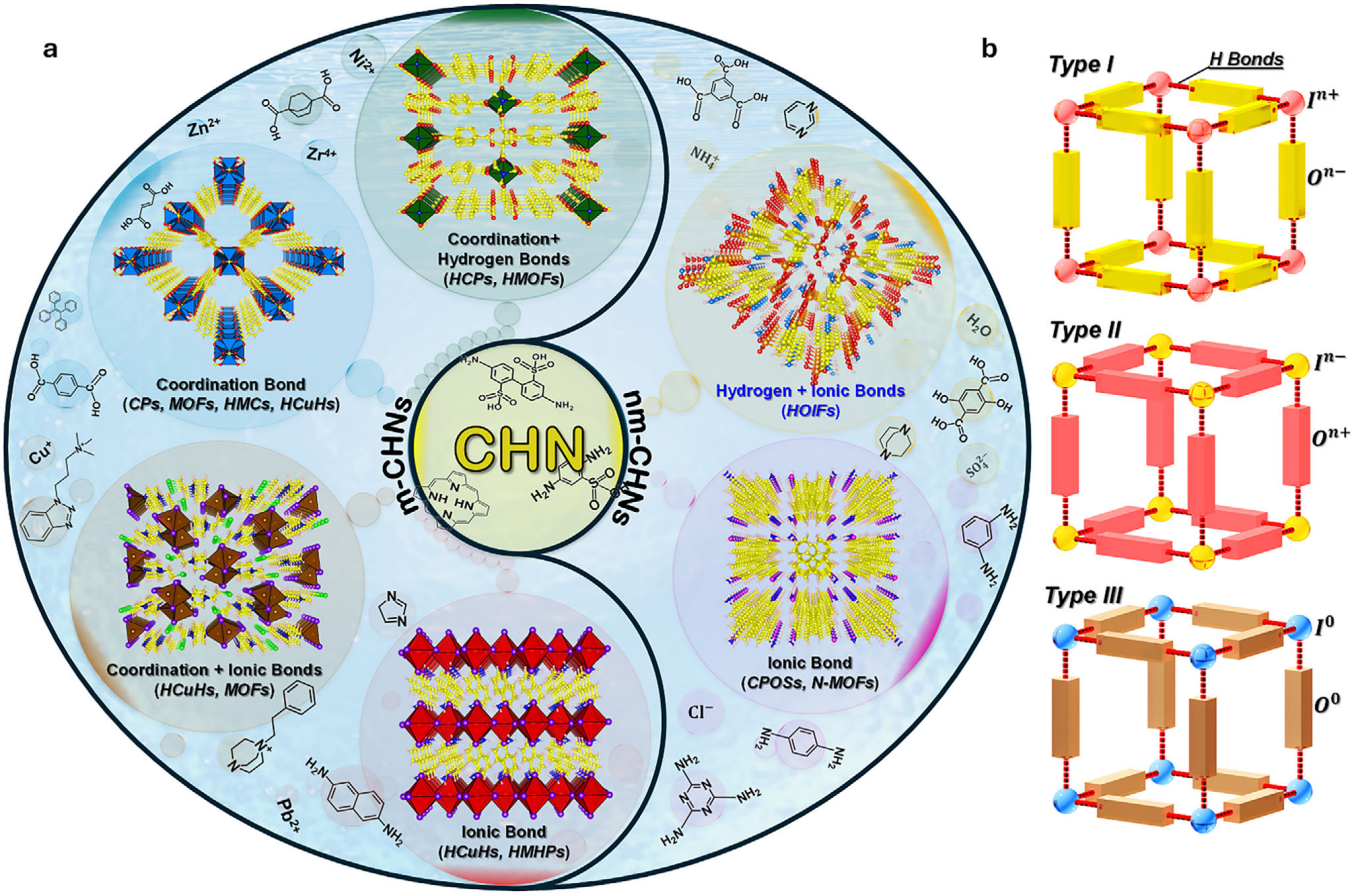
Conceptual framework for classifying CHNs and HOIFs. (a) Structural taxonomy of CHNs, divided into metal‐based (m‐CHNs) and nonmetal (nm‐CHNs) systems, including coordination polymers (CPs), MOFs, hybrid metal chalcogenides (HMCs), hybrid copper halides (HCuHs), hydrogen‐bonded CPs (HCPs), hydrogen‐bonded MOFs (HMOFs), hybrid metal halide perovskites (HMHPs), HOIFs, CPOSs and nonmetal organic frameworks (N‐MOFs). (b) Classification of HOIFs that integrate organic (O) and inorganic (I) components with different charge characteristics through ionic or neutral hydrogen bonding.

Crystalline hybrid networks (CHNs) integrate both organic and inorganic constituents in their framework structures. Nevertheless, existing CHNs face inherent limitations for intrinsic proton conduction. Crystalline porous organic salts (CPOSs) incorporating ionic inorganic nodes (e.g., Na^+^, Cl^−^) rely primarily on nondirectional electrostatic interactions, and neutral building blocks are absent in these compounds [22, 23, 24, 25]. More broadly, metal‐based CHNs (Figure 1a), most notably metal‐organic frameworks (MOFs), feature metal coordination centers that often disrupt continuous H‐bonding pathways. Consequently, pristine MOFs typically exhibit poor proton conductivity and require strong Brønsted acid functionalization, which demands exceptional stability and complicates processing [26, 27, 28, 29].

Within this landscape, we hypothesized that replacing metal coordination nodes with metal‐free and small inorganic building blocks that can act as either H‐bond donors or accepters‐capable of forming dense, directional and continuous H bonds in both ionic and neutral states – would afford uninterrupted H‐bonding networks with high H‐bond density, thereby enabling intrinsically high σ. Following this design, we have assembled a family of metal‐free nonporous CHNs using both compact organic and inorganic building units with high density of H‐bond acceptors/donors. We refer to this material class as hydrogen‐bonded organic‐inorganic frameworks (HOIFs, Figure 1a), as their structural characteristics and hybrid nature cannot be fully encompassed by the existing classification of HOFs, CPOSs and their extended families (e.g. iHOFs, nonmetal organic frameworks or N‐MOFs) (Figure S1) [22, 23]. Particularly, most of the HOIFs are nonporous as the porosity is not a necessary requirement for inherent and fast proton conduction in these structures.

**FIGURE 2:**
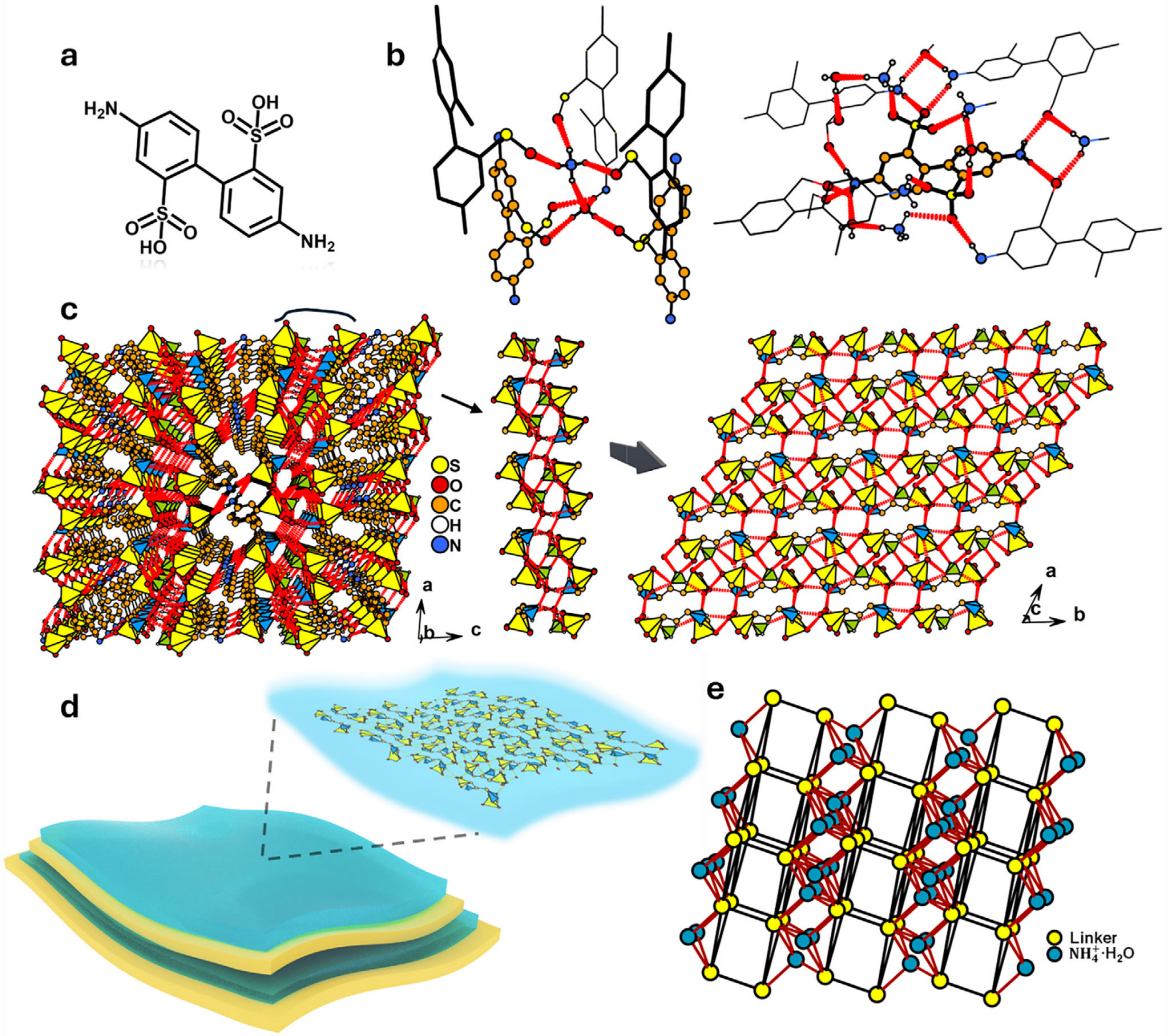
Structural features of HOIF‐101 and its organic linker. (a) Chemical structure of DADS. (b) Local hydrogen‐bonding environment and connectivity of inorganic cations (left) and organic anions (right) in HOIF‐101. Color scheme for the atoms: H, white; C, orange; N, blue; O, red; S, yellow. Selected phenyl rings and functional groups are simplified or omitted for clarity. (c) 3D crystal structure and hydrogen‐bonding layers of HOIF‐101 viewed along the *b*‐ and *c*‐axes (yellow tetrahedra, sulfonate groups; blue tetrahedra: protonated amino groups or ammonium cations). (d) Schematic illustration highlighting the layered architecture: the blue regions depict the 2D hydrogen‐bonding networks, while the yellow regions correspond predominantly to the organic phenyl backbone, with minor hydrogen‐bonding interactions retained. (e) Topological representation of the newly identified ***hoi*** net in HOIF‐101, with organic components in yellow and inorganic components in blue.

Herein, we report a suite of HOIFs containing two‐ and three‐dimensional (2D‐3D) H‐bonding networks. They can be grouped into three categories based on charge characteristics of the organic and inorganic building units (Figure 1b): Type I, organic anions + inorganic cations (HOIF‐101–104); Type II, organic cations + inorganic anions (HOIF‐201–204); and Type III, neutral organic + inorganic components (HOIF‐301/302), in which stoichiometric, crystallographically resolved water molecules act as connectivity‐bearing units within the extended H‐bonded networks. Single‐crystal X‐ray diffraction (SC‐XRD) analysis reveals novel structural types and topologies across the suite. Systematical impedance analysis verifies the efficacy and generalizability of such a hybridization strategy to enhance proton conductivity. Notably, the σ values surpass their organic counterparts by 1–5 orders of magnitude. HOIF‐101, assembled from 4,4′‐diaminobiphenyl‐2,2′‐disulfonic acid (DADS, Figure 2a) and ammonium hydroxide, adopts a new topology designated as ***hoi***. HOIF‐101, without additional functionalization, achieves a σ of 3.19 × 10^−2^ S·cm^−1^ at 85°C and 100% relative humidity (RH), firmly within the superprotonic regime (≥10^−4^ S·cm^−1^) [30]. It outperforms most MOF and HOF analogues [20, 30, 31, 32]. An integrated analysis of SC‐XRD, molecular dynamics (MD) simulations, density function theory (DFT) calculation, x‐ray photoelectron spectroscopy (XPS), in‐situ Fourier transform infrared spectroscopy (FTIR) and solid‐state nuclear magnetic resonance (ssNMR) indicate a favorable 2D proton conducting pathway in HOIF‐101, in which proton pumping mediated by ammonium species accounts for the high proton conductivity.

## 2 Results and Discussion

### 2.1 Synthesis and Structure Analysis

The synthesis of HOIFs is fast, straightforward, low‐cost, and readily scalable, unlike typical MOFs which often require multi‐day procedures to yield crystalline, thermodynamically stable products [33]. Type I HOIFs were synthesized by reacting ammonia hydroxide with DADS, 2,5‐dihydroxyterephthalic acid (BDCDH), 1,3,5‐benzenetricarboxylic acid (BTC) and 1,4‐benzenedicarboxylic acid (BDC) to afford HOIF‐101∼104, respectively. Type II HOIFs were obtained by reacting sulfuric acid with 2,5‐diaminobenzenesulfonic acid (DAS), melamine (MA), 1,4‐phenylenediamine (BDA) and 1,3‐phenylenediamine (mBDA) to yield HOIF‐201∼204, respectively (Figure S1). Type III HOIFs were formed from zwitterionic DADS or DAS and stoichiometric water molecules that directly bridge the organic components and define the extended H‐bonded connectivity, affording HOIF‐301 and HOIF‐302, respectively. Single crystals suitable for SC‐XRD analysis were grown via slow evaporation or vapor diffusion method, with acetone employed as the antisolvent (Figures S2–S4). Powder x‐ray diffraction (PXRD) patterns confirmed the phase purity and structural consistency between bulk powders and single crystals, distinct from parent organic counterparts (Figures S5 and S6).

The crystal structures of the suite were determined by SC‐XRD, revealing nine new structures (Tables S1–S3). Detailed H‐bonding motifs and connectivity were resolved (Figures S7–S22), with novel topologies observed in each case (Table S4). For instance, HOIF‐101, a new compound with an unprecedented structure, crystalizes in triclinic *P* $\bar{1}$ space group with a formula of [C_12_H_6_(NH_2_)(${\mathrm{NH}}_{3}^{+}$)(${\mathrm{SO}}_{3}^{-}$)_2_]∙[(${\mathrm{NH}}_{4}^{+}$)∙(H_2_O)] (Table S1). Within HOIF‐101, each organic linker is deprotonated as an anion to connect with four pairs of inorganic ammonia‐water cations as well as surrounding six linkers through hydrogen bonding (Figure 2b), while each ammonia‐water cluster is linked by five DADS anions, collectively generating the ***hoi*** topology with 5,10‐c binodal net (point symbol, {3^3^.4^7^}{3^6^.4^18^.5^18^.6^3^}) (Figure 2b,e). Sulfonate and protonated amino groups of organic linkers closely align with ammonia clusters to form a continuous, dense H‐bonding layer in (001) lattice plane, whereas the remaining neutral amino groups of adjacent linkers interact mutually in the interlayer to construct the 3D framework structure (Figure 2c,d). Thermogravimetric analysis (TGA) demonstrated high thermal stability of these HOIFs (Figures S23 and S24).

### 2.2 Proton Conducting Properties

The HOIF materials were expected to have proton conducting properties given their high H‐bond density and continuous 2D and 3D H‐bonding networks. Proton conductivity (*σ*) was systematically evaluated via alternating current (AC) impedance analysis (Figure 3a,b and Figures S25–S31).

**FIGURE 3:**
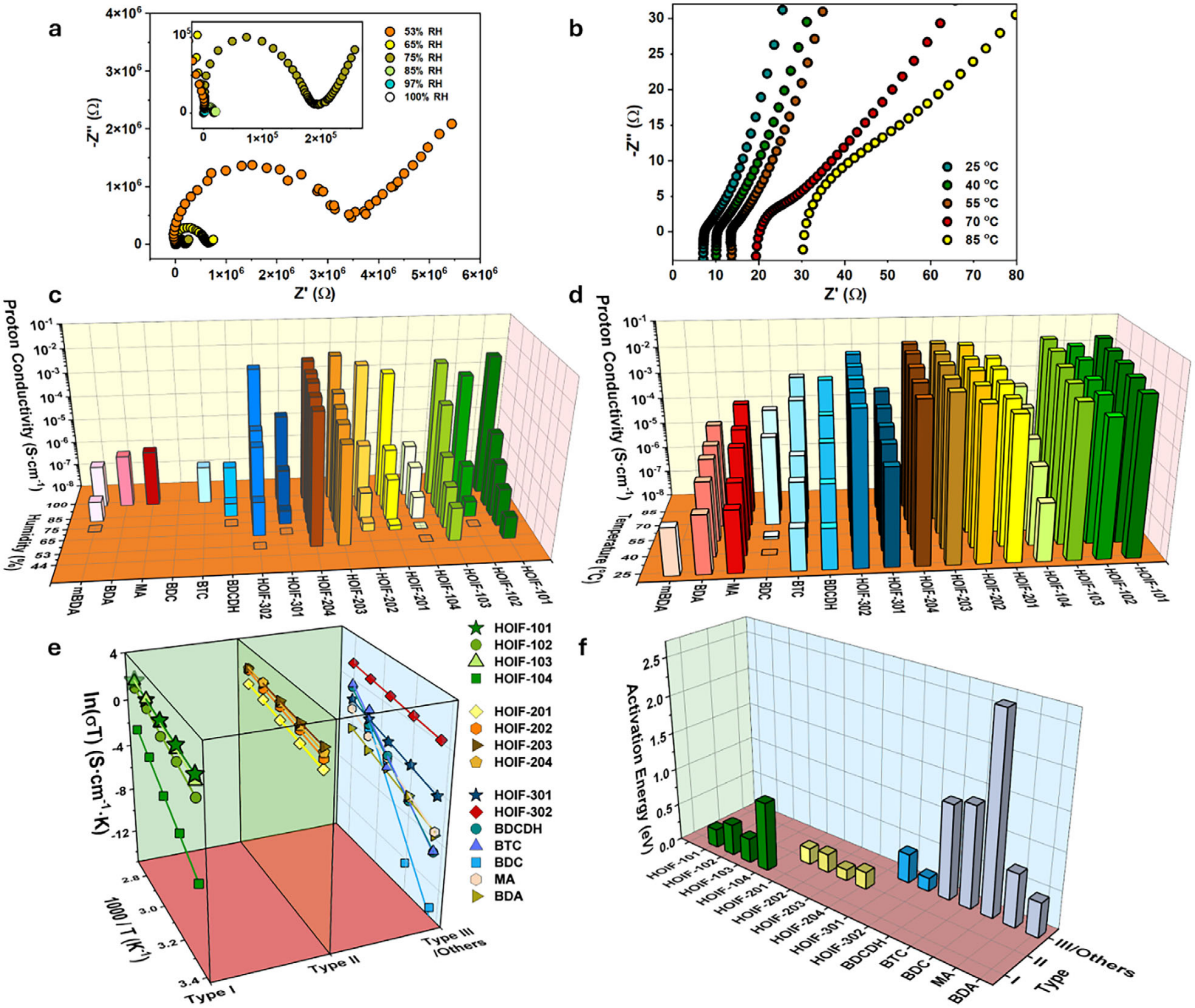
Proton‐conducting behavior of the HOIF series. (a,b) Nyquist plots of HOIF‐101 measured under different relative humidities at 25°C (a) and different temperatures at 100% RH (b). (c,d) Proton conductivity of HOIF‐10X (X = 1–4), HOIF‐20X (X = 1–4), HOIF‐30X (X = 1–2) and their parent analogues as a function of RH (c) and temperature (d). (e,f) *Arrhenius* plots (e) and derived activation energies (f) for all three types of HOIFs and the parent counterparts.

All HOIFs exhibit superprotonic conductivity (>10^−4^ S·cm^−1^) at 85°C, 100% RH [30], with most surpassing 10^−2^ S·cm^−1^ (Figure 3c,d and Table S5), underscoring the intrinsic proton conducting nature of this type of materials as well as the versatility of such a hybridization strategy. Compared with their parent organic counterparts, HOIFs show conductivity enhancement ranging from one to five orders of magnitude (Table S5), e.g., *σ* increasing by >10 000 fold from 3.66 × 10^−7^ S·cm^−1^ in BTC to 4.51 × 10^−3^ S·cm^−1^ in HOIF‐103 at 25°C, 100% RH. Such metal‐free hybridization is superior to those involving metals for enhancing *σ*. For example, pristine MOFs without additional modification naturally display low *σ*, even below those of the starting organic ligands (Table S6). Across the suite, HOIF‐101 exhibits the highest *σ*, 3.19 × 10^−2^ S·cm^−1^, at 85°C, 100% RH (Figure S32 and Table S5), outperforming most MOFs and HOFs (Table S7). It is also interesting to note that HOIF‐10X (X = 1–4) employed a Brønsted base, ammonia hydroxide, for the hybridization and *σ* improvement, which is in sharp contrast to conventional strategies relying on Brønsted acids, further highlighting the novelty and efficiency of the hybridization approach [34, 35].

The conductivity of HOIFs under low humidity was also significantly improved, whereas some of their organic building units were below measurable limits (Figure 3c). A humidity‐dependent σ under wide relative humidity (RH) range is particularly advantageous for practical application such as humidity sensing in monitoring human health and agroforestry management [6, 36]. It is intriguing that *σ* in HOIF‐203 and HOIF‐204 is relatively independent on humidity in the examined RH range (Figure 3c), an essential property for the application in humidity‐sensitive scenarios such as fuel cells [26]. In addition, hybridization also increased the stability of HOFs, for example, mBDA tended to degrade at elevated temperature (>25°C), while its counterpart, HOIF‐204, remained robust and achieved *σ* > 10^−2^ S·cm^−1^ across 40°C–85°C, with the highest *σ* of 2.58 × 10^−2^ S·cm^−1^ (Figure 3d and Table S5).

This hybridization strategy also significantly reduces the activation energy (*E_a_*) for proton transport (Figure 3e,f and Table S5). Specifically, most HOIFs have *E_a_* less than 0.4 eV (e.g., 0.25, 0.22, 0.39 eV for HOIF101, −201, −301, respectively; see Table S5), indicative of a Grötthuss‐type proton conducting mechanism where protons transfer by hopping in a well‐linked H‐bonding system, with lower energy barrier accounting for the high *σ* [34]. The reduced E_a_ values are consistent with the higher hydrogen‐bond density and network continuity engineered through metal‐free hybridization. By contrast, the parent organic counterparts lacking such continuous hydrogen‐bonded networks show *E_a_* greater than 0.4 eV (e.g., 0.46, 0.71, 1.36 eV for BDA, MA, BTC, respectively; Table S5), indicating a Vehicle‐type mechanism involving proton transport via the diffusion of proton carriers, *e.g*. H_2_O [30].

### 2.3 Mechanism Studies

To gain deeper insight into the proton conducting mechanism, HOIF‐101 that exhibited the best *σ* was studied in detail, with HOIF‐301 as reference.

NMR spectra suggested the retention of chemical structure of the organic building block upon hybridization (Figure S35), ruling out molecular structure effect on *σ* enhancement. Scanning electron microscopy with energy dispersive x‐ray spectroscopy (SEM‐EDX) and XPS excluded the presence of any metals in this type of hybrid materials (Figures S36 and S37), proving the vital role of metal‐free inorganic building blocks in σ enhancement. FTIR and XPS studies revealed the presence of both protonated and deprotonated amino groups in HOIF‐101, which are critical in maintaining 2D hydrogen‐bonding layers and 3D structure respectively (Figure S38). These findings are consistent with SC‐XRD results. Interestingly, HOIF‐101 also shows good photoluminescent properties (Figure S39), indicating potential applications in sensing, anticounterfeiting, information storage/encryption [37, 38].

**FIGURE 4:**
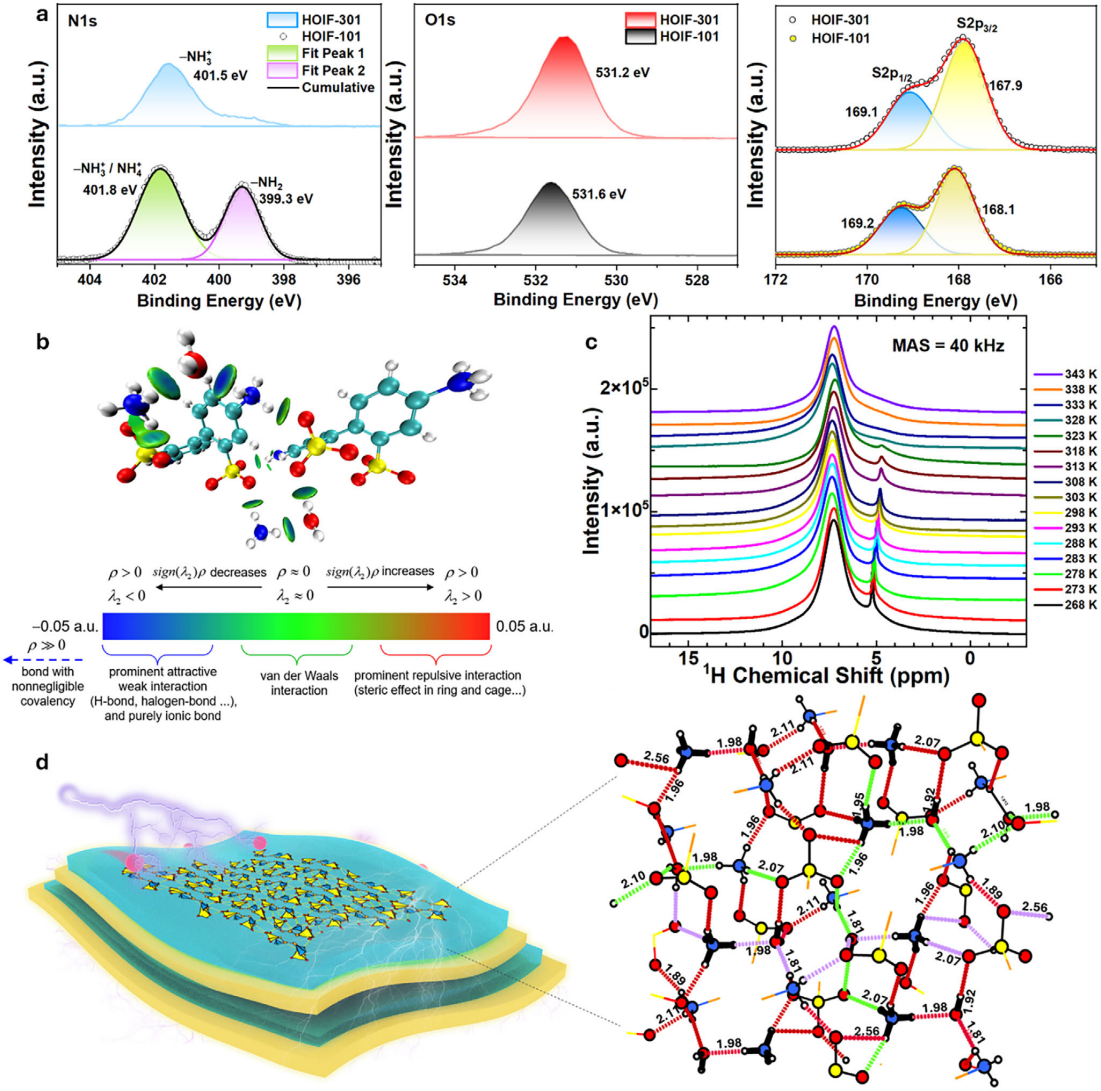
Mechanism studies of proton conductivity in HOIF‐101. (a) High‐resolution XPS spectra of N 1s (left), O 1s (middle), S 2p (right) regions for HOIF‐101 and HOIF‐301. (b) Independent Gradient Model based on Hirshfeld partition (IGMH) isosurfaces for HOIF‐101. Color scheme for atoms: C, cyan; O, red; H white; N, blue; S, yellow. The color scale represents the value of sign(λ_2_)ρ, where blue, green, and red correspond to attractive (hydrogen bonding), van der Waals, and repulsive (steric repulsion) interactions, respectively. (c) Solid‐state ^1^H NMR spectra of HOIF‐101 at different temperatures (MAS = 40 kHz). (d) Illustration of proton conduction based on the dense 2D layers of hydrogen‐bonding networks in HOIF‐101. Color scheme for atoms: C, orange; O, red; H white; N, blue; S, yellow.

The nonporous nature of HOIF‐101, as revealed by N_2_/CO_2_ isotherms and PLATON analysis with Brunauer‐Emmett‐Teller (BET) surface area < 10 m^2^·g^−1^ (Figure S40), yielded high density of H bonds in the structure, which could be beneficial to proton migration. Indeed, as shown in Figure S41, proton conductivity correlates positively with H‐bond density across the HOIF family. Detailed H‐bond analysis further revealed that HOIF‐101 possesses higher H‐bond density (56.4 vs. 52.2 mol·L^−1^ compared to HOIF‐301), longer donor (D) – acceptor (A) distances (3.31 vs. 3.02 Å) and broader H‐bond length distribution (0.17 vs. 0.09, standard deviation) features that facilitate long‐range proton transport by enabling continuous and smooth conducting pathways (Figure S42 and Tables S8–S10). In (001) plane, HOIF‐101 exhibits extended 2D H‐bonding networks with geometries ideally suited for proton hopping (Figure 4d and Tables S8 and S9).

Importantly, the absence of permanent pores does not preclude hydration; instead, water can interact with accessible polar sites within the dense framework and dynamically reorganize the local H‐bonding network. Water vapor sorption analysis confirmed stronger water‐framework interactions in HOIF‐101 than HOIF‐301 (Figure S40c). Consistently, the H_2_O bending component at 1646 cm^−1^ became increasingly prominent in the in situ FTIR spectra of HOIF‐101 at higher water‐vapor pressures, while ammonia‐related and aromatic C‐H vibrations were simultaneously perturbed (Figure S43). These observations indicate cooperative hydration of multiple polar sites and the formation of an extended water–framework H‐bonding network, rather than water migration through permanent pore channels. By contrast, only limited perturbation of the aromatic C‐H modes was observed for HOIF‐301, consistent with its lower water uptake and fewer water–framework interaction modes (Figures S43 and S44).

XPS further indicated stronger H‐bonding interactions in HOIF‐101, as evidenced by higher binding energies of N 1s (401.8 vs. 401.5 eV) and O 1s (531.6 vs. 531.2 eV), compared to HOIF‐301, in line with in situ FTIR and crystallographic observations (Figure 4a and Figure S42). Specifically, both HOIF‐101 and HOIF‐301 feature three primary H‐bonding motifs, i.e., $-{\mathrm{NH}}_{3}^{+}$···OH_2_ (h1), $-{\mathrm{NH}}_{3}^{+}$···$O_{3}^{-}S-$ (h2) and $-{\mathrm{SO}}_{3}^{-}$···H_2_O (h3), whilst HOIF‐101 contains additional ${\mathrm{NH}}_{4}^{+}$‐based interactions. HOIF‐101 presents shorter average H‐bond length (H···A distance: h1, 1.91 vs. 1.81 Å; h2, 2.22 vs. 2.19 Å; h3, 2.04 vs. 1.97 Å) than HOIF‐301 (Table S11). This can be attributed to the ${\mathrm{NH}}_{4}^{+}$‐H_2_O pairing and partial H_2_O ionization in HOIF‐101, which strengthens h1 and h3 interactions and indirectly modulate h2 (Figure S45). This interpretation is further supported by DFT calculations (Table S12) and independent gradient model with Hirshfeld partition (IGMH) analysis (Figure 4b and Figure S46) [39, 40], revealing more intense proton delocalization along donor‐acceptor vectors in HOIF‐101.

Solid state NMR and T_2_ analyses show that HOIF‐101 hosted highly mobile proton species with a long T_2_ (1.6 ms at 273 K) and strong temperature‐dependent chemical shift and line broadening (Figure 4c), characteristic of an intermediate chemical exchange regime. In contrast, proton motions in HOIF‐301 remain confined to localized dynamics (Figures S47 and S48). These results provide direct evidence that the incorporation of ammonia species in HOIF‐101 activates dynamic proton exchange beyond local fluctuations as well as regulates proton mobility within dense H‐bonding hybrid networks.

To understand the atomic‐level dynamic behavior, the mean squared displacement (MSD) based on MD simulations was analyzed for atoms belonging to each specific group (H_2_O, ‐NH_2_, $-{\mathrm{NH}}_{3}^{+}$ and ${\mathrm{NH}}_{4}^{+}$) (Figure S49). In HOIF‐301, the results exhibited common features of atoms deviating from their equilibrium (minimum potential energy) positions due to thermal vibrations and fluctuations. The average deviation, as represented by the MSD, reached a steady state after 5 ps. In contrast, the results in HOIF‐101 demonstrated distinct behavior, as revealed by the abrupt (step‐like) changes in MSD associated with H in $-{\mathrm{NH}}_{3}^{+}$ and ${\mathrm{NH}}_{4}^{+}$ (and H_2_O). Closer examination of the MD trajectories confirmed that these abrupt changes corresponded to jump rotations of $-{\mathrm{NH}}_{3}^{+}$ and ${\mathrm{NH}}_{4}^{+}$, which act like pedal wheels or dynamic “proton pumps” to shuttle protons between neighboring donors and acceptors. The H_2_O molecules also underwent reorientation in this process, as indicated by the spikes in the MSD associated with H in H_2_O. These rotational and reorientational motions in HOIF‐101, which were absent in HOIF‐301, could collectively enable a relay mechanism that facilitated long‐range proton transport, when an external electric field was applied and proton defects were present.

Taken together, the incorporation of ${\mathrm{NH}}_{4}^{+}$ in HOIF‐101 enhances proton mobility by strengthening H‐bonding interactions and introducing a “pumping” effect that enables rapid proton hopping across the well‐connected H‐bond network, revealing the nature of Grötthuss mechanism and high proton conduction in this type of hybrid materials. The mobile protons arise from ${\mathrm{NH}}_{4}^{+}$/‐${\mathrm{NH}}_{3}^{+}$ with relatively weak acidity. Such dense, continuous networks with enhanced proton mobility overcome the weak acidity (Figure S45). Unlike traditional strategies to improve *σ* in a solid that depends on increasing proton sources (often through strong Brønsted acids), this approach leverages a Brønsted base to modulate proton mobility, broadening the molecular design space for high‐performance solid proton conductors.

Remarkably, HOIF‐101 exhibits excellent thermal stability and recyclability, as evidenced by consistent proton conductivity during repeated heating‐cooling cycles (Figure S50). It can be readily incorporated into flexible mixed‐matrix membranes with polyvinylpyrrolidone and polyvinylidene fluoride, achieving a maximum σ of 3.91 × 10^−2^ S·cm^−1^ at 85°C, 100% RH (Figure S51). These characteristics underscore the robustness of HOIF‐101 and highlight its potential for practical applications.

## 3 Conclusion

We have introduced a suite of nonporous HOIFs featuring superprotonic conductivity with most achieving >10^−^^2^ S·cm^−^^1^ at 85°C and 100% RH, surpassing their organic counterparts by 1–5 orders of magnitude and outperforming most reported MOFs as well as other metal‐free porous frameworks. This crystalline material class can be categorized into three sub‐families based on the charge states of their organic and inorganic building units. By integrating ionic or neutral organic linkers with metal‐free and small inorganic nodes as H‐bond donors or accepters, we have engineered a large number of densely interconnected 2D and 3D H‐bonded architectures featuring high density hydrogen bonds and efficient proton transport. Systematic crystallographic and proton conductivity studies reveal structural novelty and intrinsically high performance, demonstrating the effectiveness of this hybridization strategy for enhancing proton conduction in framework materials. Mechanistic studies on HOIF‐101 show that dynamic inorganic motifs not only reinforce hydrogen bonds but can actively modulate proton transport, serving as molecular rotors or pumps that facilitate efficient Grötthuss‐type hopping. This work establishes metal‐free hybridization as a general strategy to tune and enhance proton mobility, offering clear design principles based on hydrogen‐bond density, network continuity, and the dynamic role of inorganic nodes. The simplicity, scalability, and processability of HOIFs position them as a versatile platform for a range of applications including membrane‐based technologies.

## Author Contributions

**Jin Chen**: conceptualization, investigation, formal analysis, writing – original draft, writing – review and editing, validation, data curation. **Yongqiang Cheng**: investigation, methodology, formal analysis, data curation. **Simon J. Teat**: formal analysis, data curation, resources. **Gobin Raj Acharya**: formal analysis, data curation. **Roknuzzaman Roknuzzaman**: data curation, formal analysis. **Wensong Yu**: data curation, investigation. **Yucong Xia**: data curation, formal analysis, investigation. **Weston C. Swallow**: investigation, data curation. **Xiaoying Huang**: resources, supervision. **Xiaodong Yang**: supervision, resources. **Thomas J. Emge**: formal analysis, resources. **Kui Tan**: supervision, data curation, resources, validation, formal analysis. **Andrew J. Nieuwkoop**: supervision, resources, validation. **Lawrence J. Williams**: supervision, investigation, funding acquisition, project administration, writing – review and editing. **Jing Li**: conceptualization, investigation, funding acquisition, validation, project administration, supervision, resources, formal analysis, writing – review and editing.

## Conflicts of Interest

The authors declare no conflicts of interest.

## Supporting information




**Supporting File**: advs77910‐sup‐0001‐SuppMat.pdf.

## Data Availability

The data that supports the findings of this study are available in the supplementary material of this article. Deposition Numbers 2490175 (for HOIF‐101) and 2490177–2490187 (for HOIF‐102∼104, ‐201∼204, ‐301 and ‐302) Contain the Supplementary Crystallographic Data for this Paper. These Data Are Provided Free of Charge by the Joint Cambridge Crystallographic Data Centre and Fachinformationszentrum Karlsruhe Access Structures Service.
