# Supplementary material for: Intrinsic Superprotonic Conduction in Metal‐Free Hydrogen‐Bonded Organic–Inorganic Frameworks
Source: Adv Sci (Weinh). 2026 Sep 27:e77910. Online ahead of print. doi: 10.1002/advs.77910 (PMC13616279; doi:10.1002/advs.77910)
Supplement: Supplementary file 1 [file ADVS-9999-e77910-s001.pdf]

Supporting Information for

Intrinsic Superprotonic Conduction in Metal-Free Hydrogen-Bonded Organic-Inorganic Frameworks

Jin Chen[1], Yongqiang Cheng[2], Simon J. Teat[3], Gobin Raj Acharya[1], Roknuzzaman Roknuzzaman[4], Wensong Yu[5], Yucong Xia[1], Weston C. Swallow[1], Xiaoying Huang[6], Xiaodong Yang[5], Thomas J. Emge[1], Kui Tan[4], Andrew J. Nieuwkoop[1], Lawrence J. Williams[1,*], Jing Li[1,*]

[1] Department of Chemistry and Chemical Biology, Rutgers University, Piscataway, New Jersey 08854, United States

[2] Neutron Scattering Division, Neutron Sciences Directorate, Oak Ridge National Laboratory, Oak Ridge, Tennessee 37831, United States

[3] Advanced Light Source Lawrence Berkeley National Laboratory, 1 Cyclotron Road, Berkeley, California 94720, United States

[4] Department of Chemistry, University of North Texas, Denton, Texas 76201, United States

[5] College of Materials Science and Engineering, Huaqiao University, Xiamen 361021, China

[6] State Key Laboratory of Structural Chemistry, Fujian Institute of Research on the Structure of Matter, the Chinese Academy of Sciences, Fuzhou, Fujian 350002, China

Correspondence to: jingli@rutgers.edu; lawjw@chem.rutgers.edu

Contents

1. Materials and Methods 3
  1.1. Synthesis and Membrane Preparation 3
  1.2. Characterizations 4
  1.3. Structure Determination 4
  1.4. Impedance Measurements 5
  1.5. *In-situ* FTIR Spectroscopy 5
  1.6. Solid-State NMR Spectroscopy 5
  1.7. Computation Methods 5
2. Supplementary Figures (S1-S51) 7
3. Supplementary Tables (S1-S12) 58
4. Supplementary References 70

1. Materials and Methods

All chemicals and reagents were obtained from commercial suppliers and used without further purification.

1.1. Synthesis and Membrane Preparation

1.1.1. Synthesis of HOIF-10X

4,4'-diaminobiphenyl-2,2'disulfonic acid (34.4 mg) was mixed with ammonia hydroxide solution (200 μL 1 M), prior to 10-minute sonication. The mixture was dispersed in deionized water (800 μL) and heated at 70 °C overnight with stirring. Subsequently, the resulting solution was either subjected to acetone vapor diffusion for three days to grow elongated plated-like colorless single crystals of HOIF-101, or added with acetone as the antisolvent to precipitate the powdery HOIF-101 which was isolated by filtration under reduced pressure, washed with acetone and dried naturally (yield: 57.1%).

2,5-dihydroxyterephthalic acid (99.0 mg) was reacted with ammonia solution (500 μL 14 M) at 75 °C overnight. The resulting solution was: (i) centrifuged under 8000 rpm for 3 minutes, whose supernatant was evaporated slowly for 2 days to grow needle-like yellow single crystals of HOIF-102; (ii) added with acetone as the antisolvent to precipitate the powdery HOIF-102 (yield: 94.7%).

1,3,5-benzenetricarboxylic acid (105.1 mg) was dissolved in ammonia solution (500 μL 14 M) at room temperature. The resulting solution was either applied with acetone vapor diffusion for 1 day to afford needle-like colorless single crystals of HOIF-103 or added with acetone as the antisolvent to precipitate the powdery HOIF-103 (yield: 94.0%)

Terephthalic acid (83.1 mg) reacted with ammonia solution (500 μL 14 M) at 75 °C overnight to afford plate-like colorless single crystals of HOIF-104. The resulting solution was further subjected to acetone to increase the yield (93.9%).

1.1.2. Synthesis of HOIF-20X

2,5-diaminobenzenesulfonic acid (47.0 mg) was mixed with sulfuric acid (514 μL 0.5 M) with the aid of sonication at room temperature. The mixture experienced either slow evaporation for 12 days to grow elongated needle-like purple crystals of HOIF-201, or added with acetone as the antisolvent to precipitate the powdery HOIF-201 which was isolated by filtration under reduced pressure, washed with acetone and dried naturally (yield: 96.6%).

Melamine (126.0 mg) reacted with sulfuric acid (1 mL 1.8 M) at 80 °C for 1 day to afford block-like colorless single crystals of HOIF-202. The resulting solution was further subjected to acetone to increase the yield (91.2%).

1,4-phenylenediamine (108.1 mg) reacted with sulfuric acid (625 μL 3.7 M) at 75 °C overnight. The resulting solution was: (i) centrifuged under 8000 rpm for 3 minutes, whose supernatant was evaporated slowly for 2 days to grow block-like purple single crystals of HOIF-203; (ii) added with acetone as the antisolvent to precipitate the powdery HOIF-203 (yield: 69.0%).

1,3-phenylenediamine (108.1 mg) was dissolved in sulfuric acid (420 μL 5.4 M) at room temperature with the aid of sonication. The resulting solution experienced either slow evaporation at room temperature for 3 days to yield large elongated plated-like colorless single crystals of HOIF-204, or precipitation of powdery HOIF-204 by acetone as the antisolvent (yield: 97.3%)

1.1.3. Synthesis of HOIF-30X Single Crystals

Large, block-like, yellow single crystals of HOIF-301 were hydrothermally grown by heating 10 mg of 4,4'-diaminobiphenyl-2,2'disulfonic acid (10 mg) in 5 mL of deionized water at 85 °C for three days.

5 mg of 2,5-diaminobenzenesulfonic acid was dissolved in 2 mL of deionized water. The resulting solution was then subjected to acetone vapor diffusion for three days, yielding plate-like pale purple

single crystals of HOIF-302.

1.1.4. Preparation of Mixed-Matrix Membrane

HOIF-101 was ground first into a fine powder and then sonically dispersed in 1 mL of *N*,*N*-dimethylformamide for 2 hours. To this suspension, 29.7 mg of poly(vinylidene fluoride) and 69.2 mg of polyvinylpyrrolidone were added, followed by stirring at room temperature for 4 hours to obtain a homogeneous gel. Subsequently, 200 µL of the gel was cast onto a 2.5 × 2.5 cm plastic plate and dried in an oven at 50 °C overnight, yielding a flexible membrane with a thickness of ~50 µm. The HOIF-101 loadings of 0%, 20%, 50%, and 80% were achieved by incorporating 0, 24.7, 98.8, and 395.4 mg of HOIF-101 fine powder, respectively.

1.2. Characterizations

Powder X-ray diffraction (PXRD) patterns were measured in the 2θ range from 5° to 40° on a Bruker D2 Phaser Powder Diffractometer with Cu-Kα (λ = 1.54056 Å, 40 kV and 30 mA). Infrared spectra were collected in the range of 4000-400 cm^{-1} by a Thermo Scientific Nicolet iS5 spectrometer with iD5 ATR attachment. Scanning electron microscopy (SEM) images were obtained on an FESEM Hitachi SU-8020 scanning electron microscope with an energy dispersive X-ray (EDX) spectroscopy. X-ray photoelectron spectroscopy (XPS) was performed on a Thermo Scientific ESCALAB 250 Xi XPS system with Al Kα radiation as the source. Thermogravimetric analysis (TGA) was performed under nitrogen gas flow (10 $mL \cdot min^{-1}$) at a heating rate of 10 °C min^{-1} from room temperature to 600 °C on a TA Instrument Q5000IR thermal gravimetric analyzer. Liquid-phase ^{1}H NMR spectra were collected on a Varian VNMRS 500 MHz. UV–Vis diffuse reflectance spectra were collected in the region 1000 to 300 nm on a Shimadzu UV-3600 UV/vis/NIR spectrophotometer equipped with an integrating sphere utilizing $BaSO_4$ as reference. The reflectance spectra were transformed using the Kubelka–Munk function, defined as $\alpha/S = (1 - R)^2 / (2R)$, where α denotes the absorption coefficient, S the scattering coefficient, and R the reflectance. Since the average particle size of the measured samples exceeded 5 µm, the scattering coefficient was considered constant. To estimate the band gap energy (E_g), the Kubelka–Munk function was further converted into the Tauc function, $(\alpha hv)^2=A(hv-E_g)$, where α (to be replaced with absorbance), h, v and A are the molar absorption coefficient, Plank's constant, frequency and absorbance, respectively.[1] For the reflectance measurements, finely ground powder samples were uniformly spread between two quartz slides. Solid-state excitation and emission spectra were recorded using a Varian Cary Eclipse spectrophotometer with an excitation wavelength of 365 nm. Internal quantum yield measurements were performed on a Hamamatsu C9220-03 spectrophotometer equipped with an integrating sphere (λ_{ex} = 365 nm). Optical microscopy was performed on an Olympus SZX2-ILLK microscope. BET surface areas were calculated from N_2 isotherms collected at 77 K as well as from CO_2 isotherms collected at 195 K on a Micromeritics 3-flex apparatus. With the same instrument, water vapor adsorption isotherms were measured at room temperature.

1.3. Structure Determination

Single crystals were selected and mounted on MiTeGen® loops with Paratone-N oil on a Bruker D8 diffractometer equipped with a PHOTONII CPAD detector and Oxford Cryosystems Cryostream 800 plus, on Beamline 12.2.1 of the Advanced Light Source at Lawrence Berkeley National Lab. A sphere of data were collected at 100 K or 298 K using Bruker APEX3 software[2] in shutterless mode with ω rotations at fixed φ values at λ= 0.7288Å, from a double crystal Silicon [111] monochromator, The intensity data were integrated and correction applied with SAINT v8.40b,[3] absorption and other correction were made using SADABS 2016/2.[4] Dispersion corrections appropriate for this wavelength were calculated using the Brennan method in XDIP[5] with in WinGX[6]. The structures were solved with a dual space method with SHELXT 2018/2 and refined using SHELXL 2019/2.[7] The finalized structures have been deposited in the Cambridge Crystallographic Data Center (CCDC 2490175 and 2490177–2490187) at www.ccdc.cam.ac.uk/data_request/cif. The crystallographic data and structural

refinement are summarized in Table S1-S3.

1.4. Impedance Measurements

Proton conductivity was measured by impedance analysis performed by a Princeton VersaSTAT 3 Potentiostat over a frequency range of 1~10^6 Hz with an amplitude of 100 mV and 0 mV DC rest voltage. The samples were finely ground and pressed into pellets of 6.4 mm in diameter and 0.7 mm in thickness and coated with silver paste on both sides to improve the contact with the silver electrodes in an electrochemical cell. The samples were equilibrated for a few hours, and in some cases for a few days, at each temperature and humidity condition before impedance measurements. Proton conductivity (σ, S·cm^{-1}) was obtained using the equation $\sigma = l/(RS)$, where l and S are the thickness and cross-sectional area of sample pellets, respectively, and R is the resistance of the sample analyzed from Nyquist plots. The activation energy (E_a) was extracted from measurements of σ over various temperatures at 100% RH using the *Arrhenius* equation: $\sigma = (\sigma_0/T)\exp[-E_a/(kT)]$, where σ_0, T and k are the pre-exponential factor, temperature in K and Boltzmann constant, respectively.

1.5. *In-situ* FTIR Spectroscopy

In situ IR measurements were performed on a Nicolet Is50 FTIR spectrometer using a liquid N_2-cooled mercury cadmium telluride (MCT-A) detector. The spectrometer is equipped with a vacuum cell that is placed in the main compartment with the sample at the focal point of the infrared beam. The as-synthesized sample (~2 mg) was gently pressed onto KBr pellet and placed into a cell that is connected to a vacuum line for evacuation. The sample was activated by evacuation at 100 ºC for ~1 h and then cooled back to room temperature for water vapor adsorption measurement.

1.6. Solid-State NMR Spectroscopy

All solid-state NMR data was acquired with a Bruker AVANCE III HD 600 MHz spectrometer using a HFXY 1.6 mm Phoenix NMR broad-band solid-state MAS probe tuned to HCN mode. Approximately 8 mg of sample was packed into each 1.6 mm sample rotor. The Magic angle spinning rates were between 15 and 40 kHz and regulated by the Phoenix MAS controller. The variable temperature set point was varied between -25 and +50 °C with the reported sample temperatures calibrated externally using ethylene glycol. The actual temperatures were +20 °C higher than the set points. The tuning frequencies of the probe for ^{1}H, ^{13}C, and ^{15}N were 599.540, 150.755, and 60.761 MHz, respectively. Typical 90 pulse widths for ^{1}H, ^{13}C , and ^{15}N nuclei were 2.2, 2.0, and 2.5 µs, respectively. Interscan delays of 2.5 and 3.5 sec were used for ^{1}H 1D and ^{1}H-^{13}C 2Ds respectively. Cross polarization at 40 kHz MAS was done with a 1.25 ms contact time and spin locks of 95 kHz on ^{1}H and a 10% Tangent ramp centered at 60 kHz on ^{13}C. Proton detected NH experiments were attempted but no signal from the natural abundance ^{15}N was observed.

1.7. Computation Methods

Molecular dynamics (MD) simulations by Density Functional Theory (DFT) were performed using the Vienna Ab initio Simulation Package (VASP).[8] The calculation used Projector Augmented Wave (PAW) method to describe the effects of core electrons,[9,10] with an energy cutoff of 500 eV for the plane-wave basis of the valence electrons. The optB86b-vdW functional for dispersion corrections was applied.[11,12] The lattice parameters and atomic coordinates determined in this work were used as the initial structure. A 2×2×2 supercell was used for the MD simulation. The supercell contains 272 H, 192 C, 48 N, 112 O, and 32 S for HOIF-101, and 288 H, 192 C, 32 N, 144 O, and 32 S for HOIF-301. The electronic structure was calculated on Γ point only. The total energy tolerance for electronic energy minimization was 10^{-4} eV. The structure was first relaxed to minimize the potential energy with default convergence criterion. MD simulations at 600 K were then performed following the canonical ensemble (NVT) implemented by Nose-Hoover thermostat. The elevated temperature was used to

accelerate the dynamics so that relevant events can be observed within the MD simulation timescale. The time step for the simulations was 0.5 fs, and a total of 20,000 steps (10 ps) were calculated for each trajectory.

Electronic structure calculations were performed at the DFT level with the Gaussian 09 software package. To preserve crystal geometry, heavy atoms were kept fixed at the experimental coordinates during single-point calculations; hydrogen atoms were only relaxed when adjustment of experimentally unresolved H-positions was required. The density functional M06-2X was employed, which is a hybrid meta-GGA functional specifically parameterized for main-group thermochemistry, kinetics, and noncovalent interactions.[13] The def2-TZVP basis set was used throughout unless otherwise specified.[14] The interaction energy was calculated based on the supermolecule approach.[17] Tight self-consistent field (SCF) convergence criteria were applied, and counterpoise corrections were carried out within the Boys–Bernardi scheme to estimate basis-set superposition errors (BSSE).[15] Intermolecular interactions were further analyzed using the independent gradient model based on Hirshfeld partition (IGMH)[16,17] as implemented in Multiwfn.[33] The electron density and its derivatives were obtained from the Gaussian checkpoint files computed at the M06-2X/def2-TZVP level. The IGMH analysis was carried out under default parameters, and the δg_inter isosurfaces were visualized with VMD[18] to qualitatively characterize the strength and nature of intermolecular hydrogen bonds and other noncovalent interactions.

2. Supplementary Figures

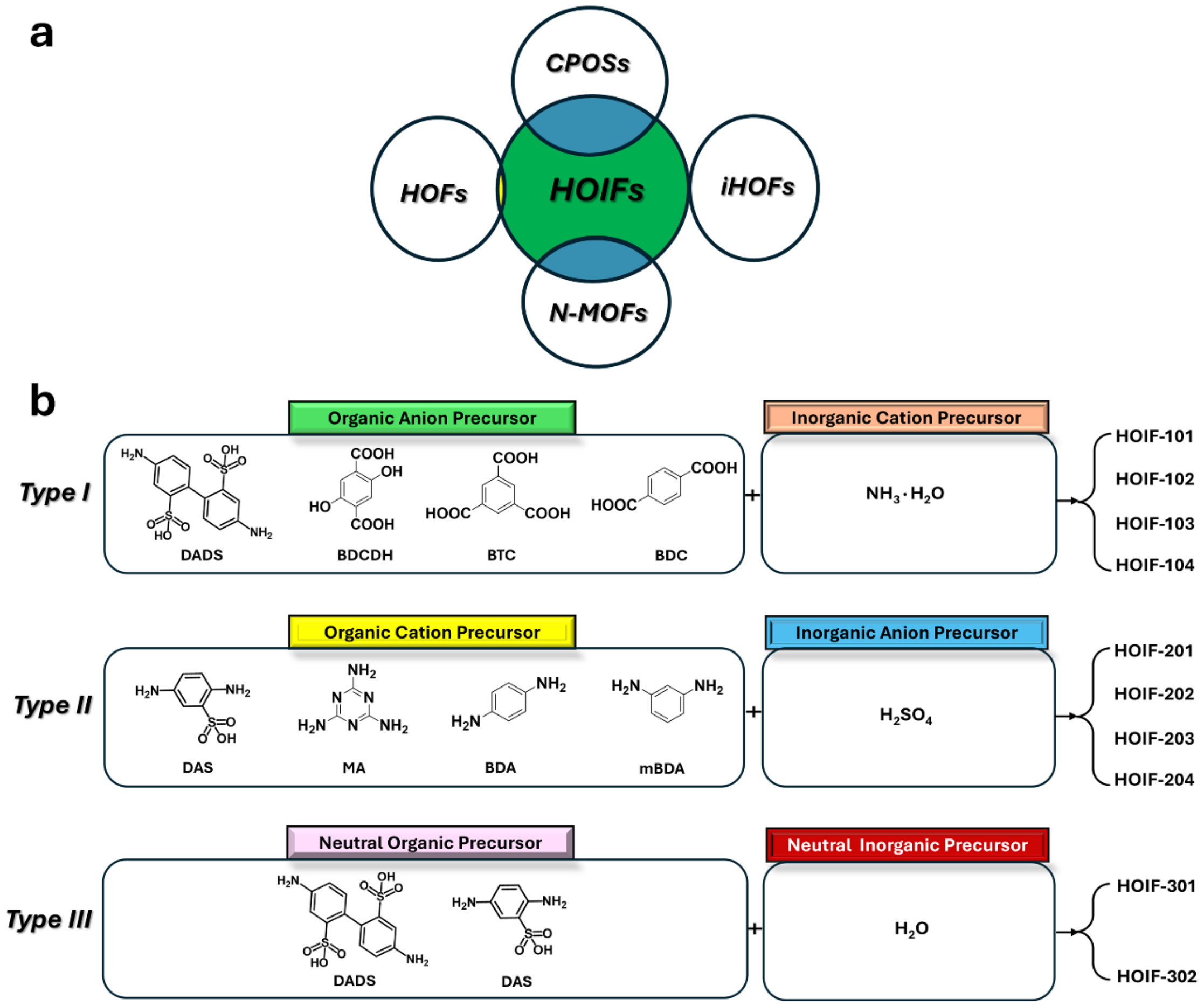

Figure S1. (a) Schematic illustration of the relationships among HOIFs (nonporous) and related porous framework families. Considering the composition and chemical bonding, HOIFs overlap with CPOSs and N-MOFs, but differ in that the latter two lack neutral building blocks. HOFs are by definition purely organic, although some reports have included inorganic species without recognizing them as equally important building blocks as their organic counterparts. All reported iHOFs are also solely organic. In contrast, HOIFs are constructed exclusively using both organic and metal-free inorganic units as proton acceptors and donors, which can be cationic, anionic, or neutral species. (b) Classification of HOIFs discussed in this work, and illustration of their individual building blocks.

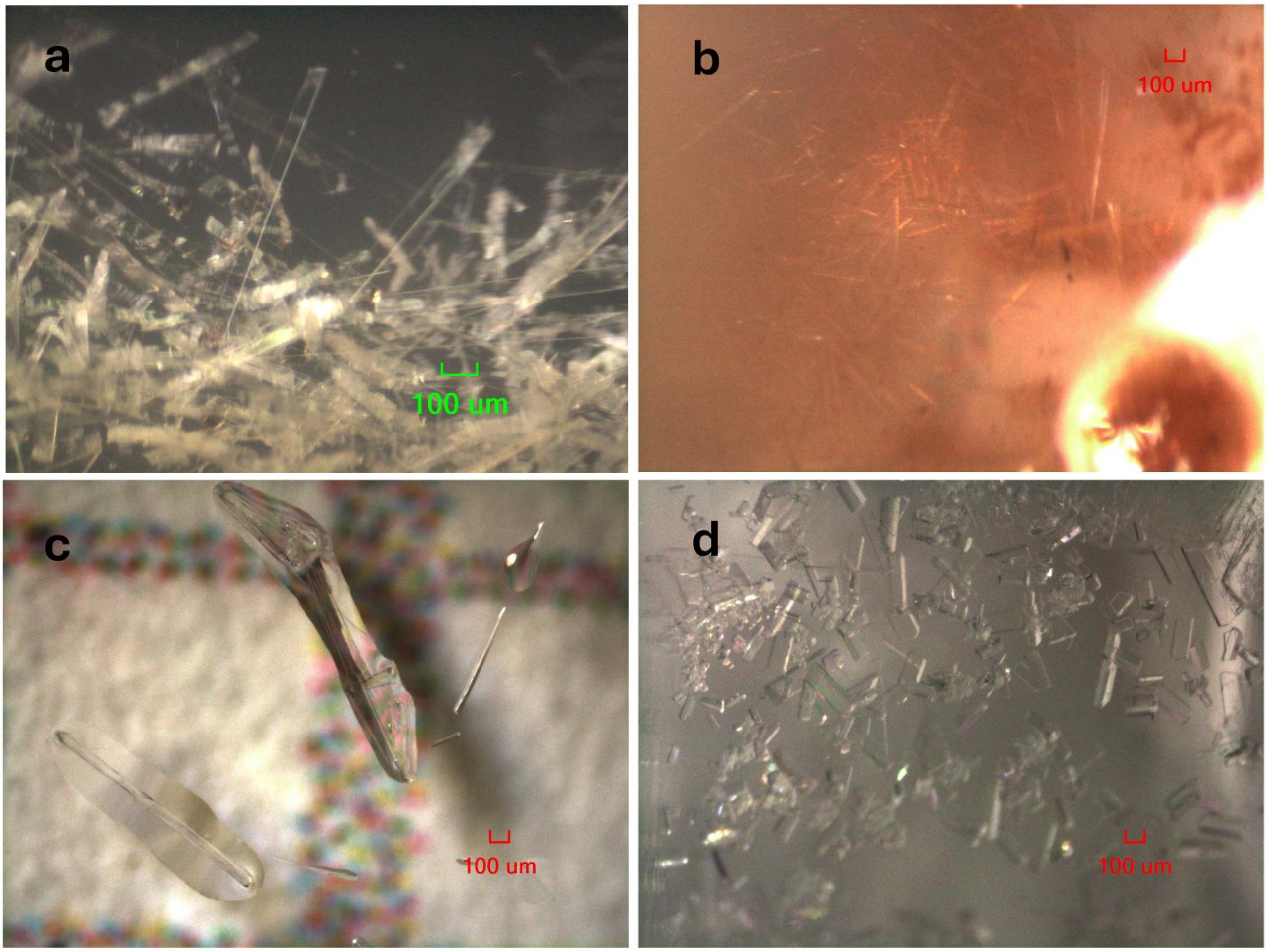

Figure S2. Microscope images of single crystals of (a) HOF-101, (b) HOIF-102, (c) HOIF-103 and (d) HOIF-104. Scale bar: 100 μm.

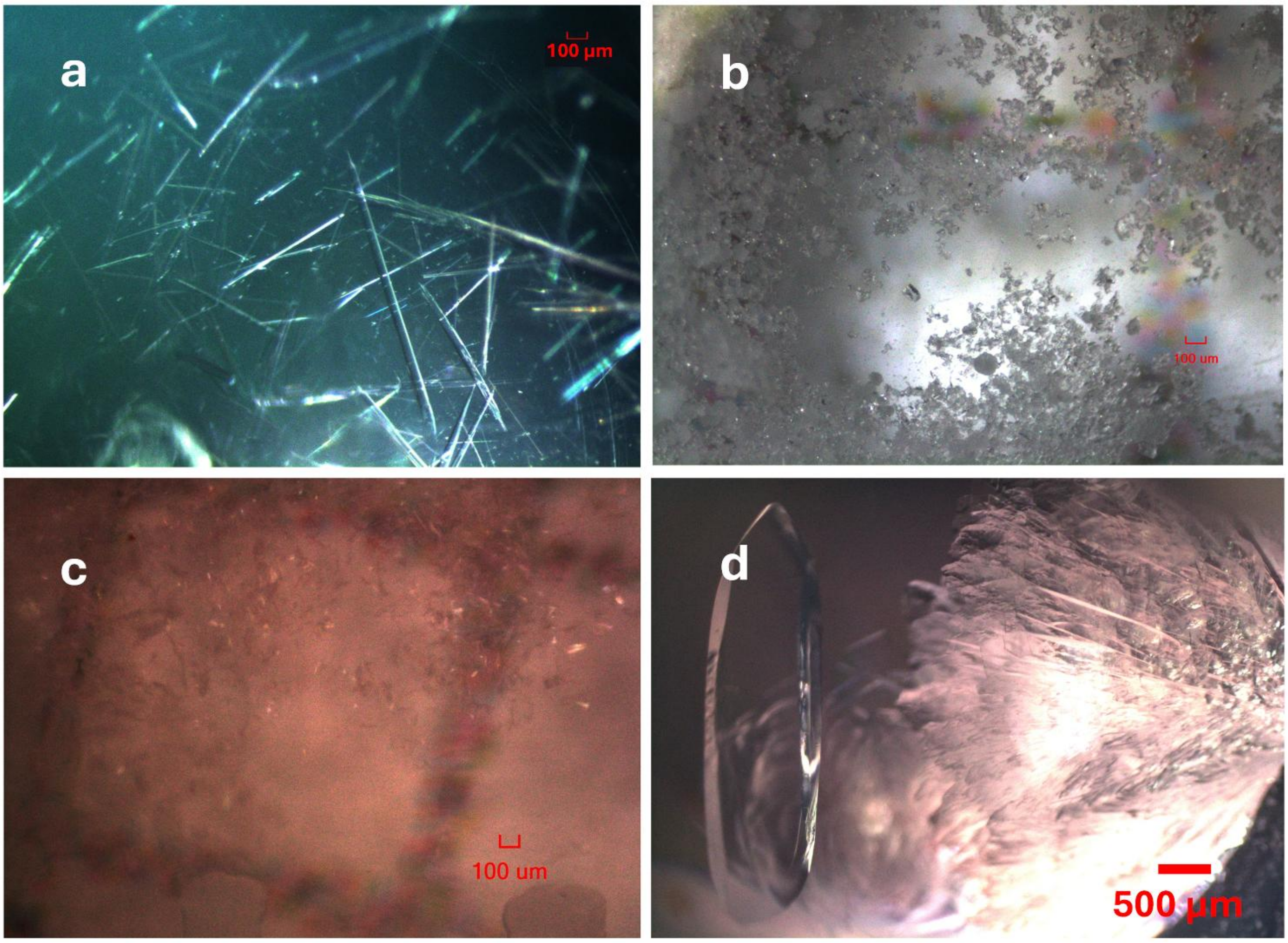

Figure S3. Microscope images of single crystals of (a) HOF-201, (b) HOIF-202, (c) HOIF-203 and (d) HOIF-204. Scale bar: 100 μm for (a)-(c); 500 μm for (d).

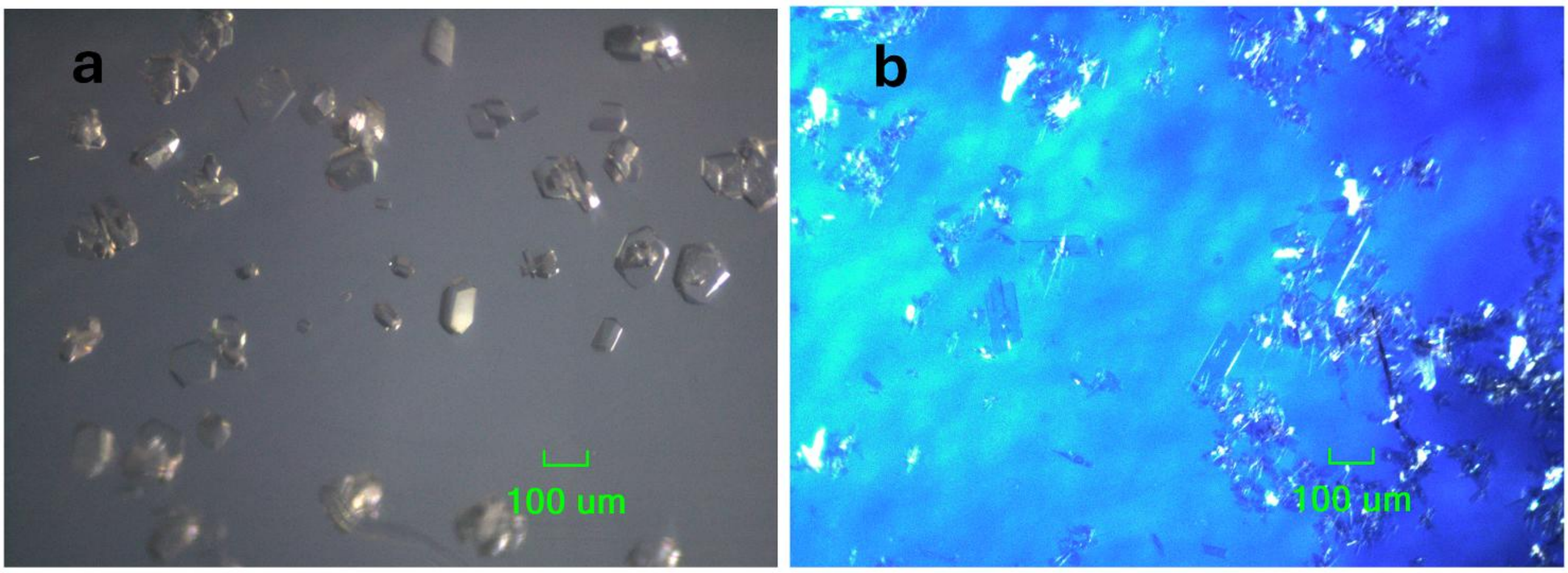

Figure S4. Microscope images of single crystals of (a) HOF-301, (b) HOIF-302. Scale bar: 100 μm.

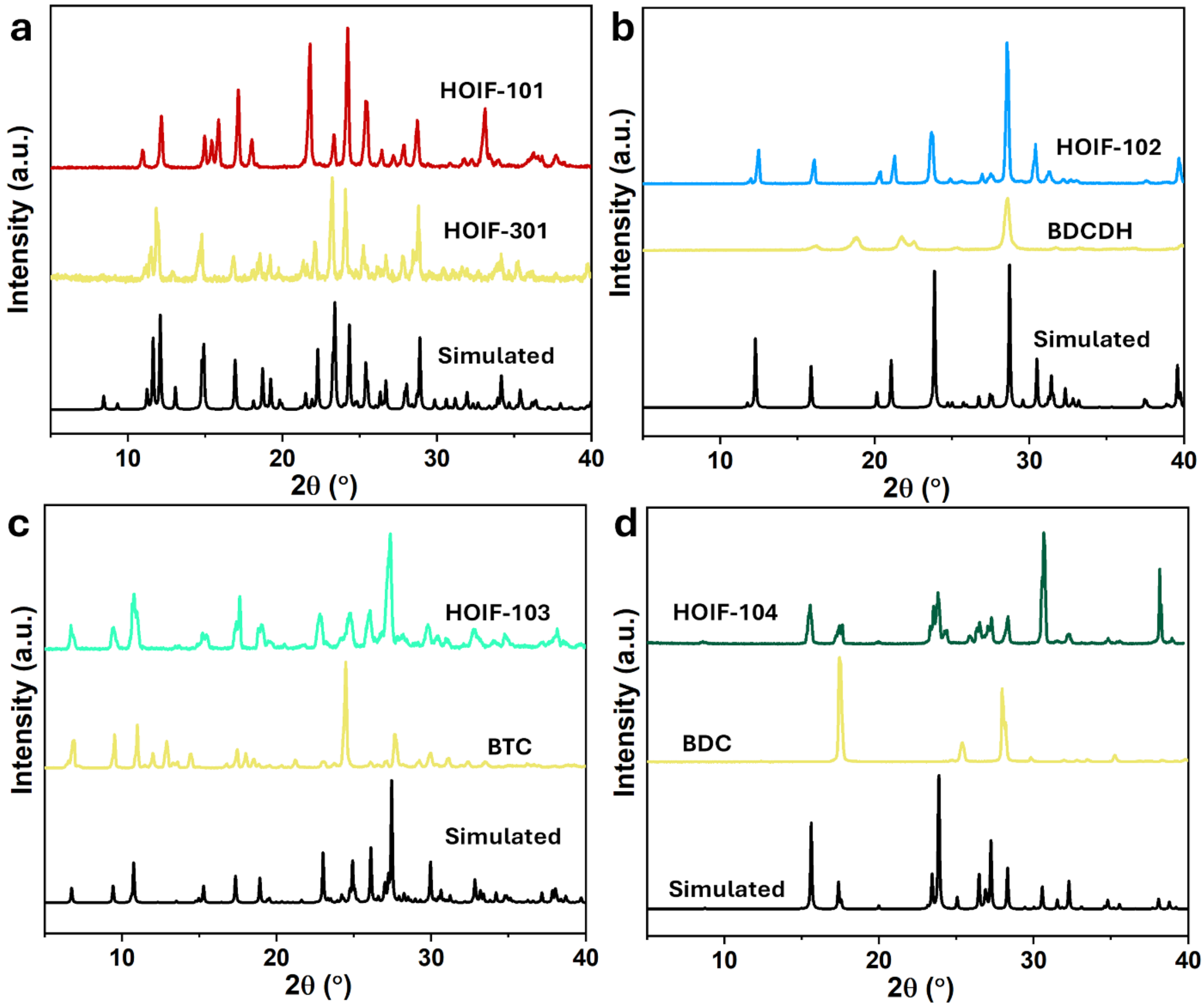

Figure S5. PXRD patterns of simulated and as-prepared (a) HOIF-101 and HOIF-301, (b) HOIF-102, (c) HOIF-103, and (d) HOIF-104, as well as their parent organic counterparts.

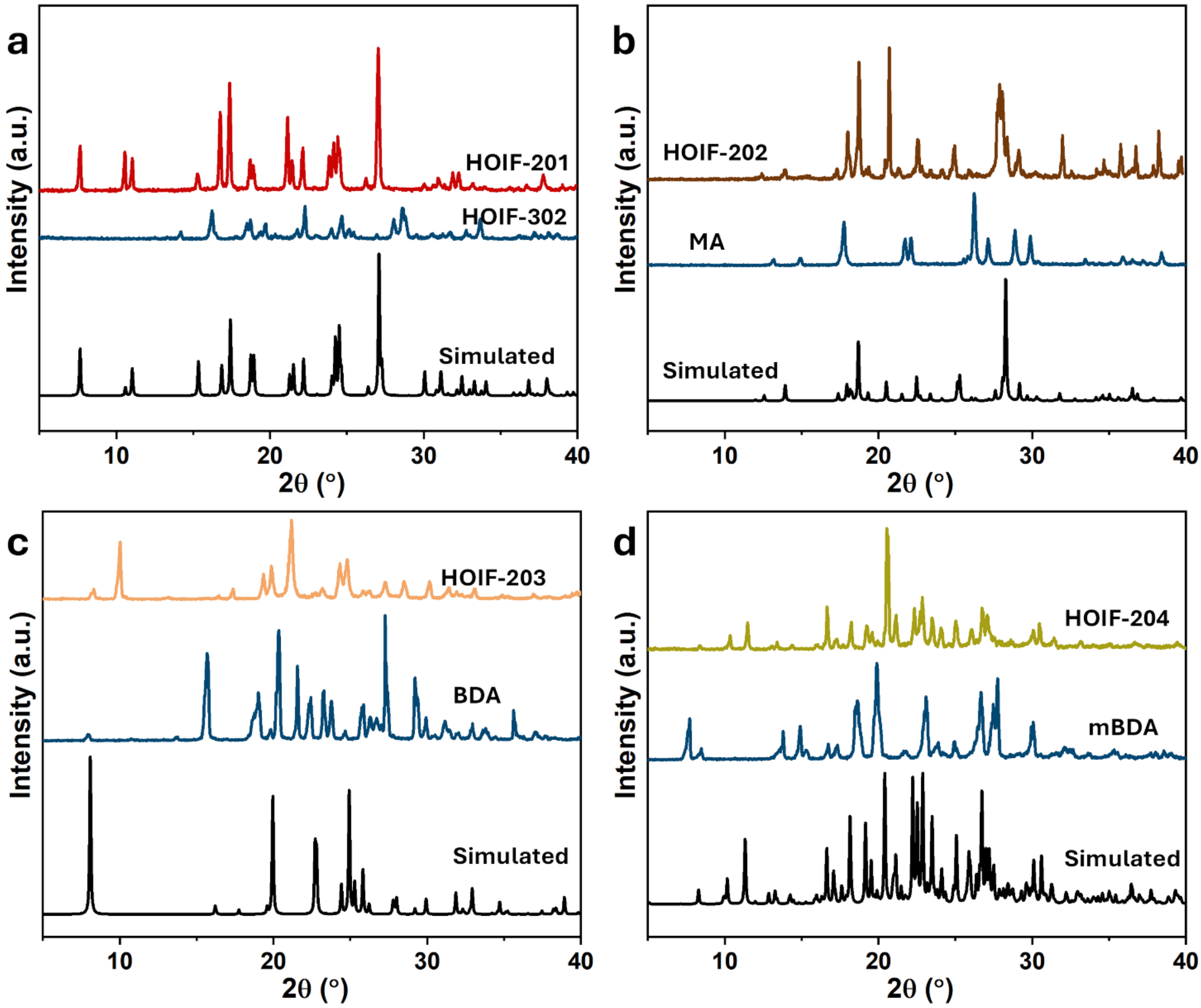

Figure S6. PXRD patterns of simulated and as-prepared (a) HOIF-201 and HOIF-302, (b) HOIF-202, (c) HOIF-203, and (d) HOIF-204, as well as their parent organic counterparts.

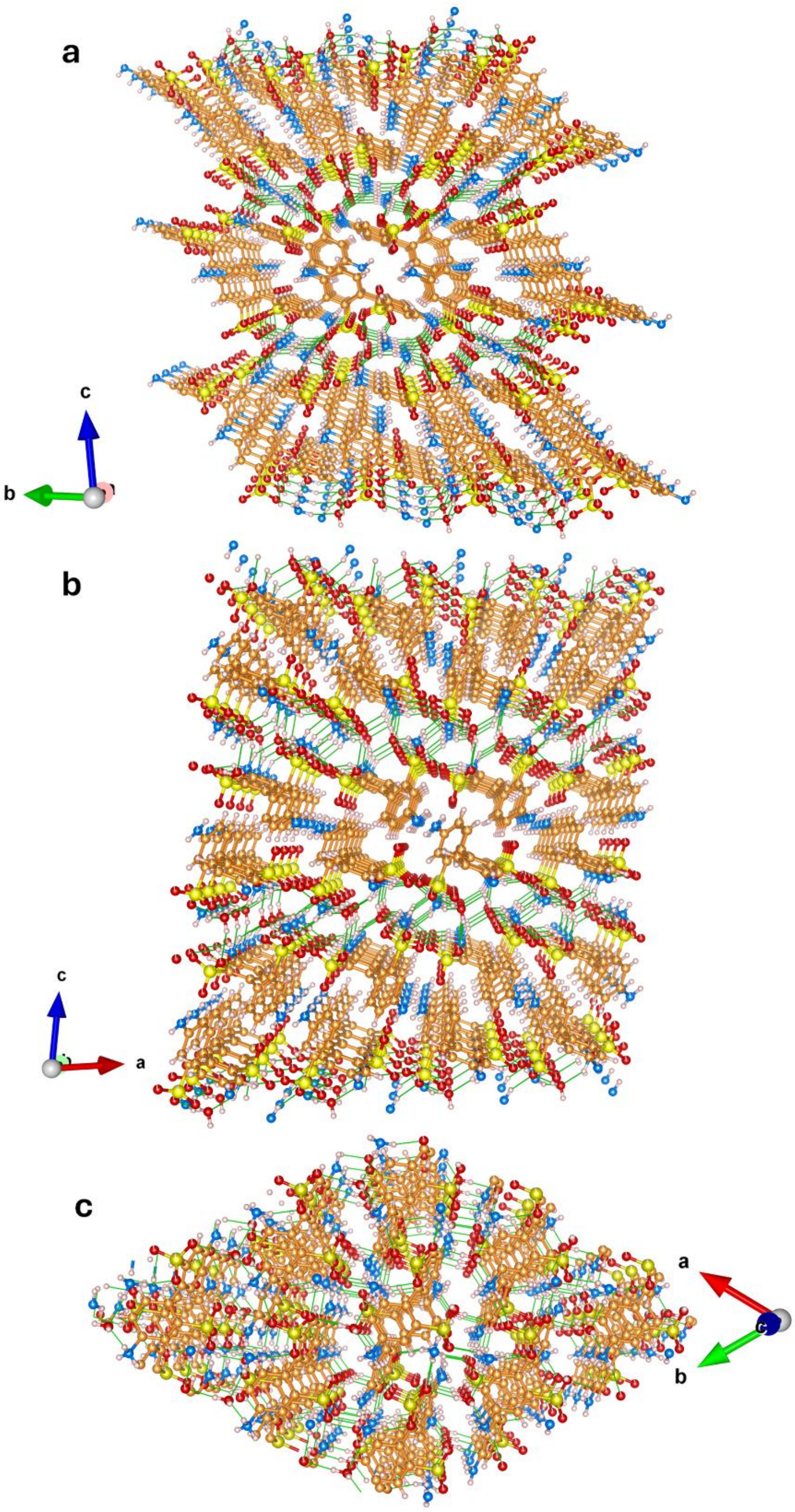

Figure S7. Crystal structure of HOIF-101 viewed along (a) *a*-, (b) *b*- and (c) *c*-axis. Color scheme: carbon, orange; oxygen, red; nitrogen, blue; sulfur, yellow; hydrogen, pink. Notably, this is a new structure with a space group of triclinic $P\overline{1}$. Within the structure, there are condensed 2D hydrogen-bonding networks in *ab* plane, which are further bridged by dangling amino groups in the interlayers. While protons are capable of transporting in three dimensions, a 2D proton conducting mode is favored.

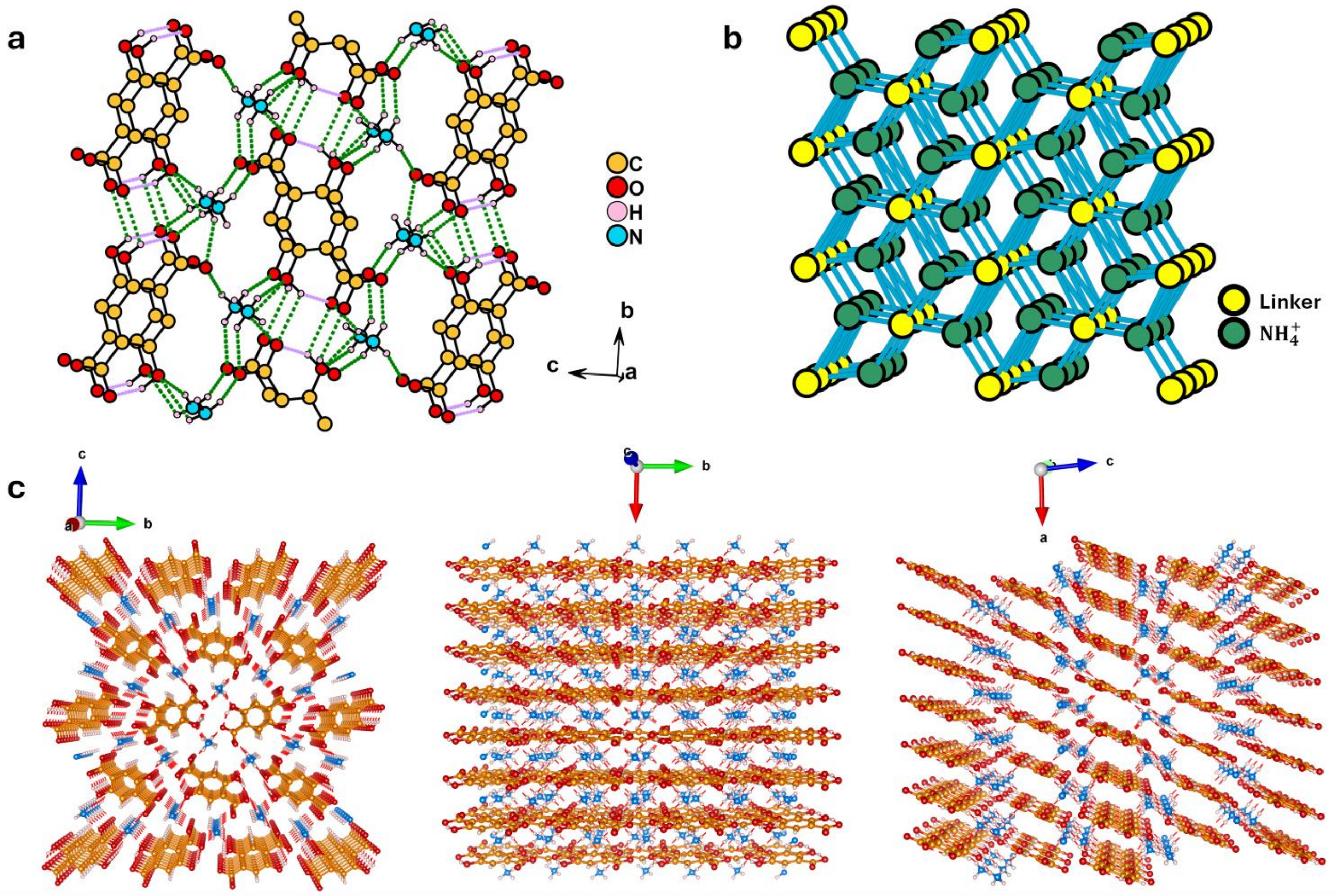

Figure S8. (a) Zoom-in view of crystal structure of HOIF-102, showing hydrogen bonds and connectivity between organic and inorganic components. (b) Topology of HOIF-102, a novel type of topology designated as ***coa***, with 4,8-c, 2-nodal net, point symbol: $\{4^{10}.6^{14}.8^4\}\{4^5.6\}_2$. (c) Crystal structure of HOIF-102 in 3D view along *a*, *b* and *c* direction. Color scheme: carbon, orange; oxygen, red; nitrogen, blue; hydrogen, pink. HOIF-102 presents a new structure with monoclinic $P2_1/n$ space group. A layered structure is constructed by hydrogen bonds between organic anions and ammonia ions in $(10\overline{3})$ plane. With Td geometry, ammonia ions further bridge adjacent layers to yield 3D networks. The intramolecular hydrogen bonds of the organic molecules also contribute to the hydrogen-bonding networks, facilitating the 3D proton conducting mode.

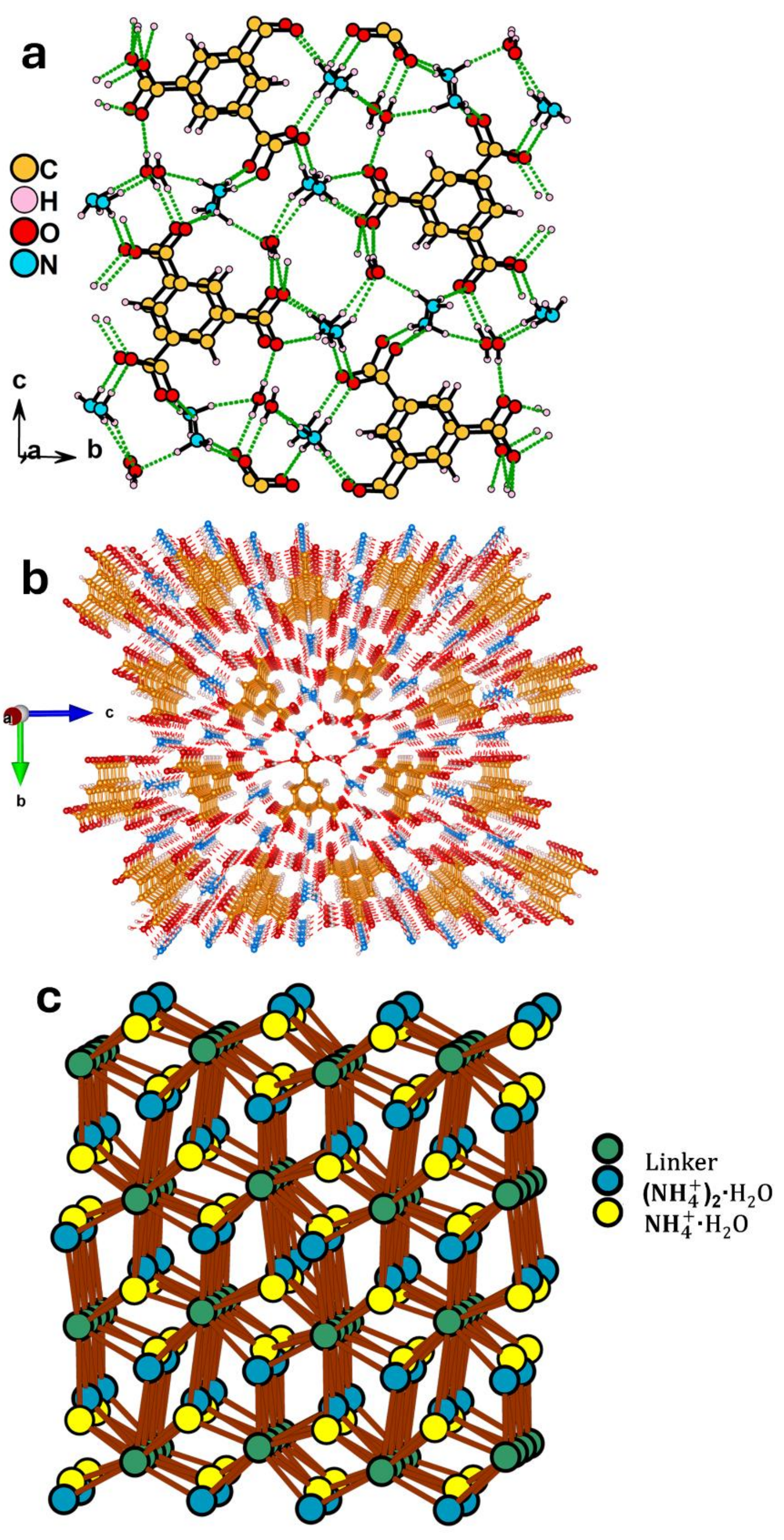

Figure S9. (a) Crystal structure of HOIF-103 showing hydrogen bonds and connectivity between organic and inorganic components. (b) 3D view of HOIF-103 structure along *a* direction. Color scheme: carbon, orange; oxygen, red; nitrogen, blue; hydrogen, pink. (c) Topology of HOIF-103, a novel type of topology designated as ***cta***, with 5,5,5,6,10,11-c, 6-nodal net, point symbol: $\{4^{10}\}_3\{4^{13}.6^2\}\{4^{28}.6^{17}\}\{4^{32}.6^{23}\}$. HOIF-103 presents a new structure with monoclinic $P2_1/n$ space group. Organic anions are bridged by ammonia/H_2O cluster cations to afford 3D hydrogen-bonding networks.

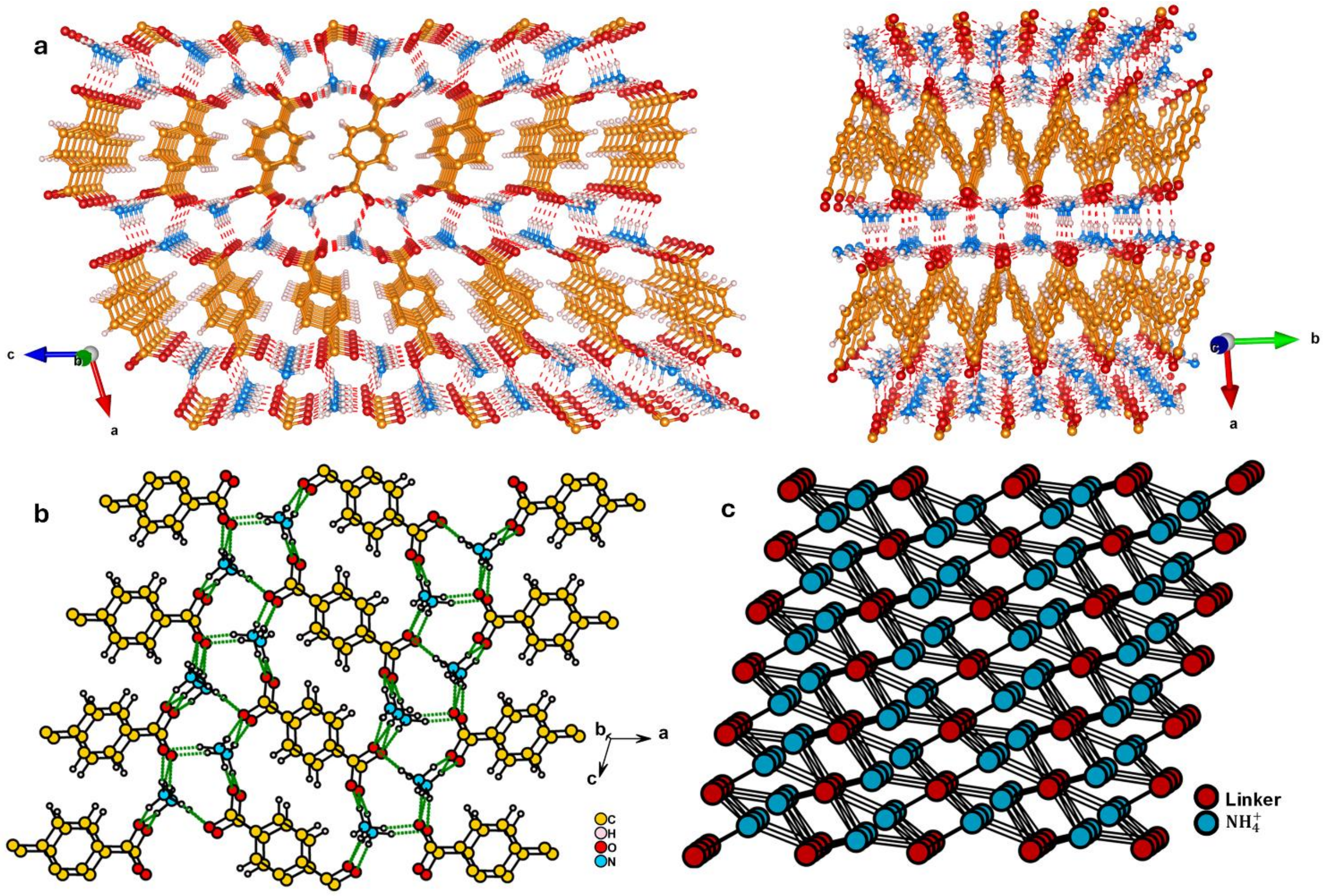

Figure S10. (a) Crystal structure of HOIF-104 in 3D view along *b* and *c* direction. Color scheme: carbon, orange; oxygen, red; nitrogen, blue; hydrogen, pink. (b) The hydrogen bonds and connectivity between organic anions and inorganic cations in HOIF-104. (c) Topology of HOIF-104, a novel type of topology designated as ***cca***, with 5,10-c binodal net, point symbol: $\{4^{26}.6^{18}.8\}\{4^{9}.6\}_2$. HOIF-104 presents a new structure with a space group of monoclinic $P2_1/c$. Organic anions are bridged by ammonia cations, yielding a zigzag-like 2D hydrogen-bonding networks in *bc* plane.

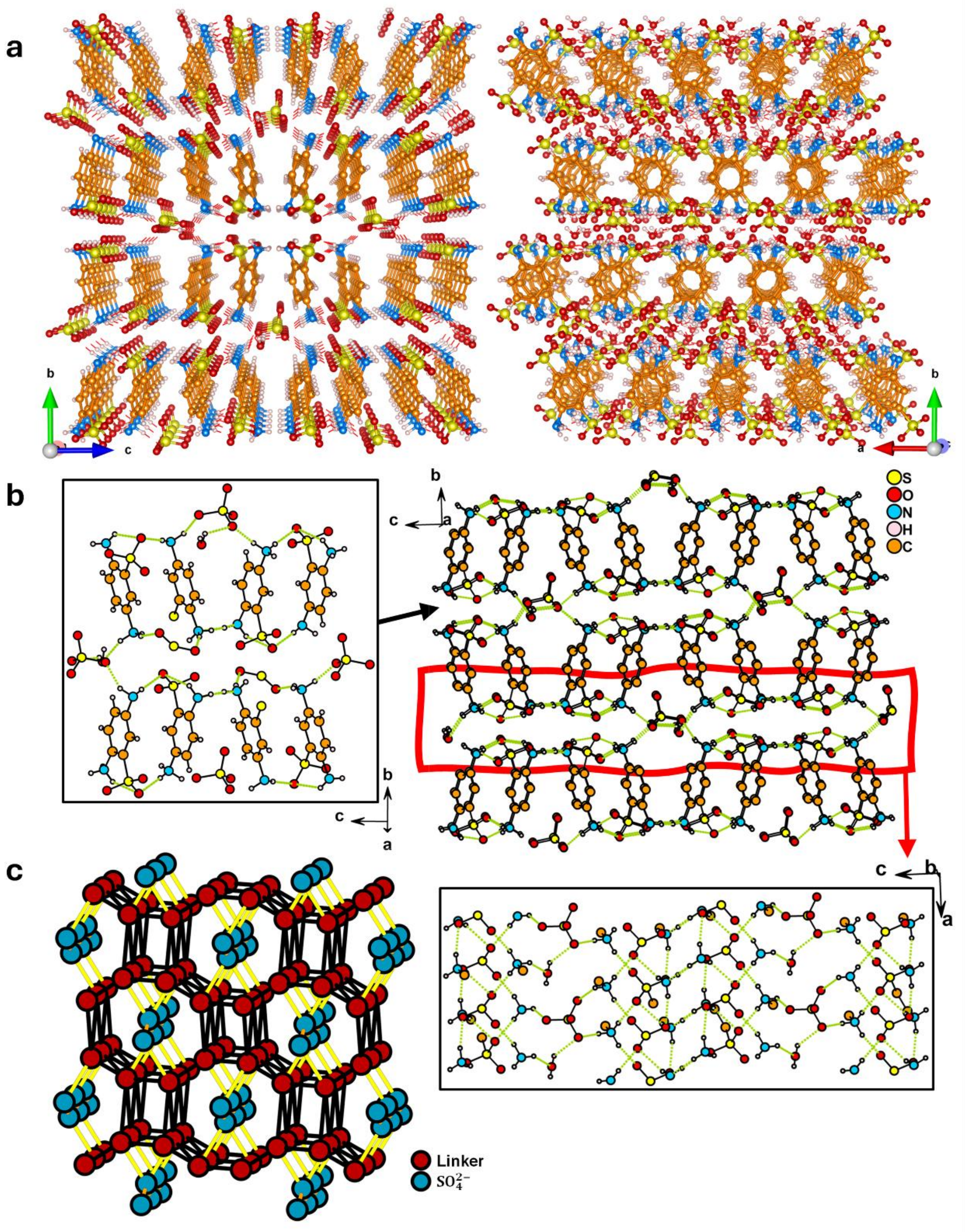

Figure S11. (a) Crystal structure of HOIF-201 in 3D view along *a* and *c*-axis. Color scheme: carbon, orange; oxygen, red; nitrogen, blue; sulfur, yellow; hydrogen, pink. (b) The hydrogen bonds and connectivity between organic cations and inorganic anions in HOIF-201. (c) Topology of HOIF-201, a novel type of topology designated as ***hio***, with 4,8-c binodal net, point symbol: $\{3^6.4^{12}.5^8.6^2\}_2\{4.5^5\}$. HOIF-104 showcases a new structure with a space group of monoclinic *I*2/*a*. The cationic/zwitterionic organic components linked mutually through amino and sulfonate groups, which is further assisted by inorganic sulfate anions to form a 2D hydrogen-bonding networks in *ac* plane.

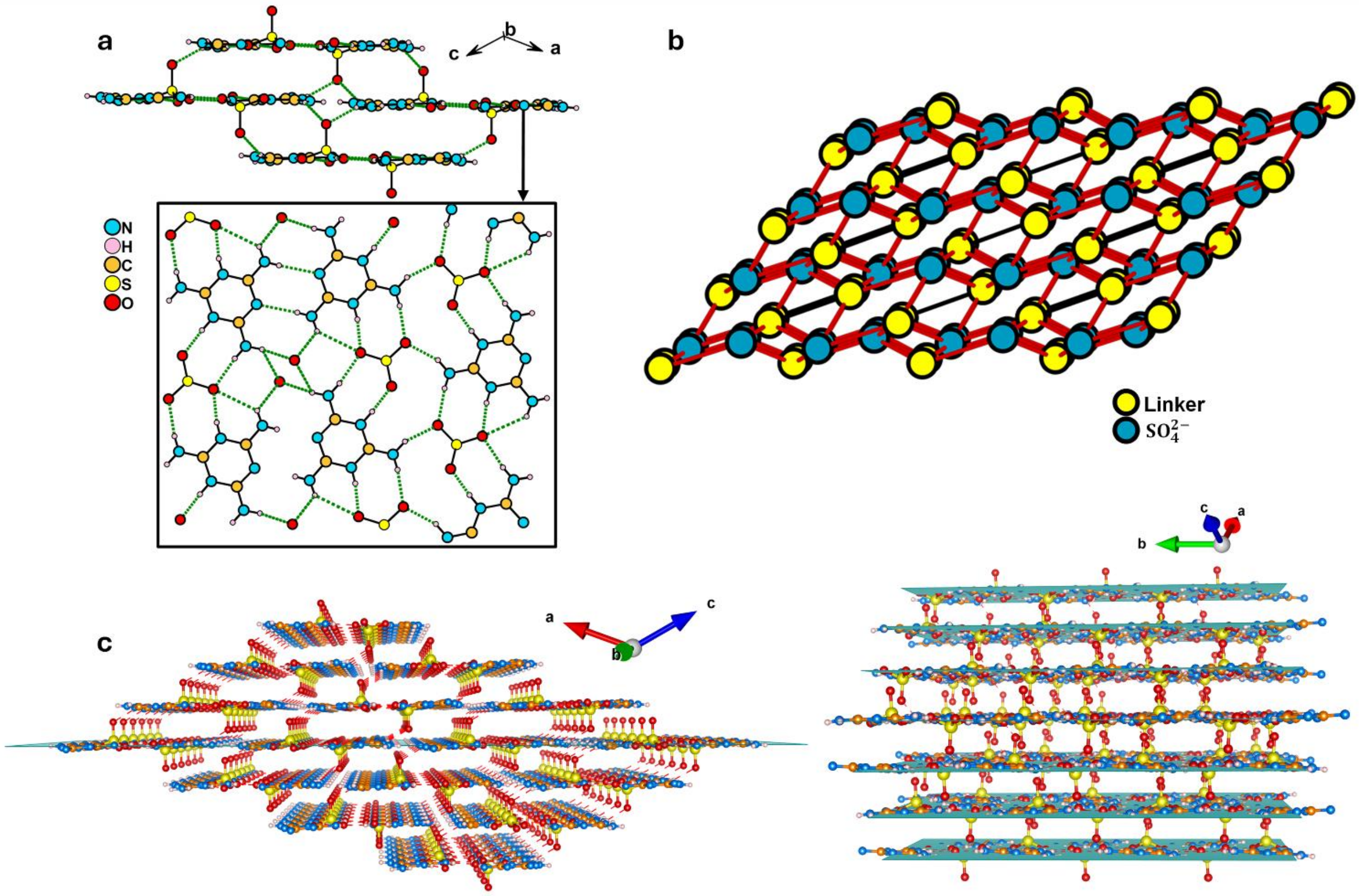

Figure S12. (a) Crystal structure representing hydrogen bonds and connectivity between organic cations and inorganic anions in HOIF-202. (b) Topology of HOIF-202, a novel type of topology designated as ***mes***, with 6,7-c binodal net, point symbol: $\{3.4^{10}.5^{3}.6\}\{3^{2}.4^{11}.5^{5}.6^{3}\}$. (c) Crystal structure of HOIF-202 in 3D view, color scheme: carbon, orange; oxygen, red; nitrogen, blue; sulfur, yellow; hydrogen, pink. HOIF-202 crystalizes in a space group of monoclinic *C*2/*c*. The organic cations form multiple hydrogen bonds with the inorganic sulfate anions, leading to a layered structure with a dense 2D hydrogen-bonding networks in (101) plane.

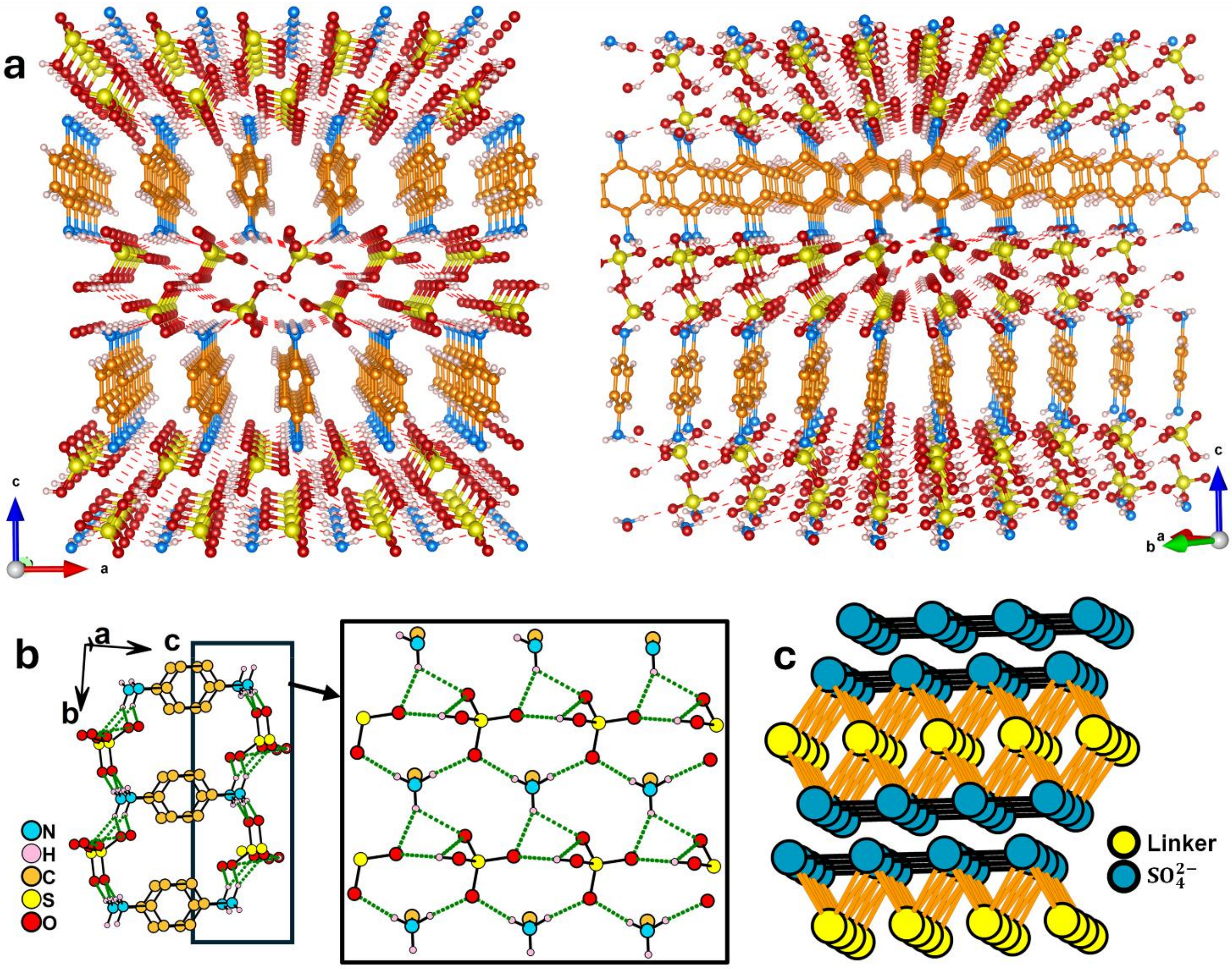

Figure S13. (a) Crystal structure of HOIF-203 in 3D view, color scheme: carbon, orange; oxygen, red; nitrogen, blue; sulfur, yellow; hydrogen, pink. (b) Crystal structure representing hydrogen bonds and connectivity between organic cations and inorganic anions in HOIF-203. (c) Topology of HOIF-203, a novel type of topology designated as ***pas***, with 6,8-c binodal net, point symbol: $\{3^4.4^{16}.5^8\}\{3^4.4^6.5^4.6\}_2$. HOIF-203 presents a new structure with a space group of monoclinic $P2_1/n$. The organic cations form multiple hydrogen bonds with the inorganic sulfate anions through protonated amino groups, constructing 2D hydrogen-bonding networks in *ab* plane.

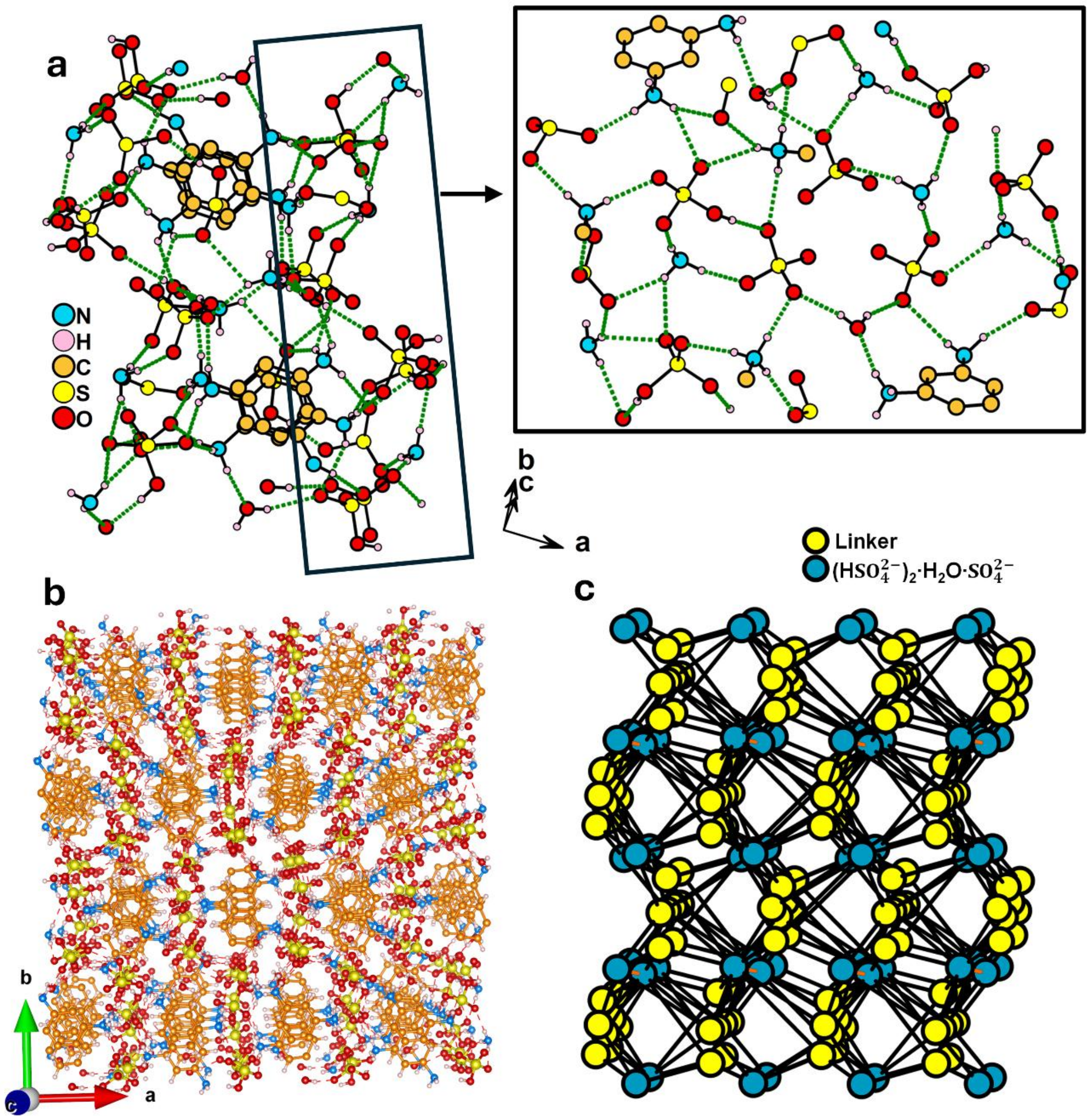

Figure S14. (a) Crystal structure representing hydrogen bonds and connectivity between organic cations and inorganic anions in HOIF-204. (b) Crystal structure of HOIF-204 in 3D view, color scheme: carbon, orange; oxygen, red; nitrogen, blue; sulfur, yellow; hydrogen, pink. (c) Topology of HOIF-204, a novel type of topology designated as ***mas***, with 4,5,5,5,9,12-c, 6-nodal net, point symbol: $\{3.4^7.5.6\}\{3.4^8.5\}\{3^2.4^5.5^3\}\{3^4.4^{17}.5^4.6^{11}\}\{3^4.4^{22}.5^6.6^{30}.7^2.8^2\}\{4^5.6\}$. HOIF-204 presents a new structure with a space group of monoclinic $P2_1/c$. The organic cations form multiple hydrogen bonds with the inorganic sulfate anions through protonated amino groups, forming 3D hydrogen-bonding networks.

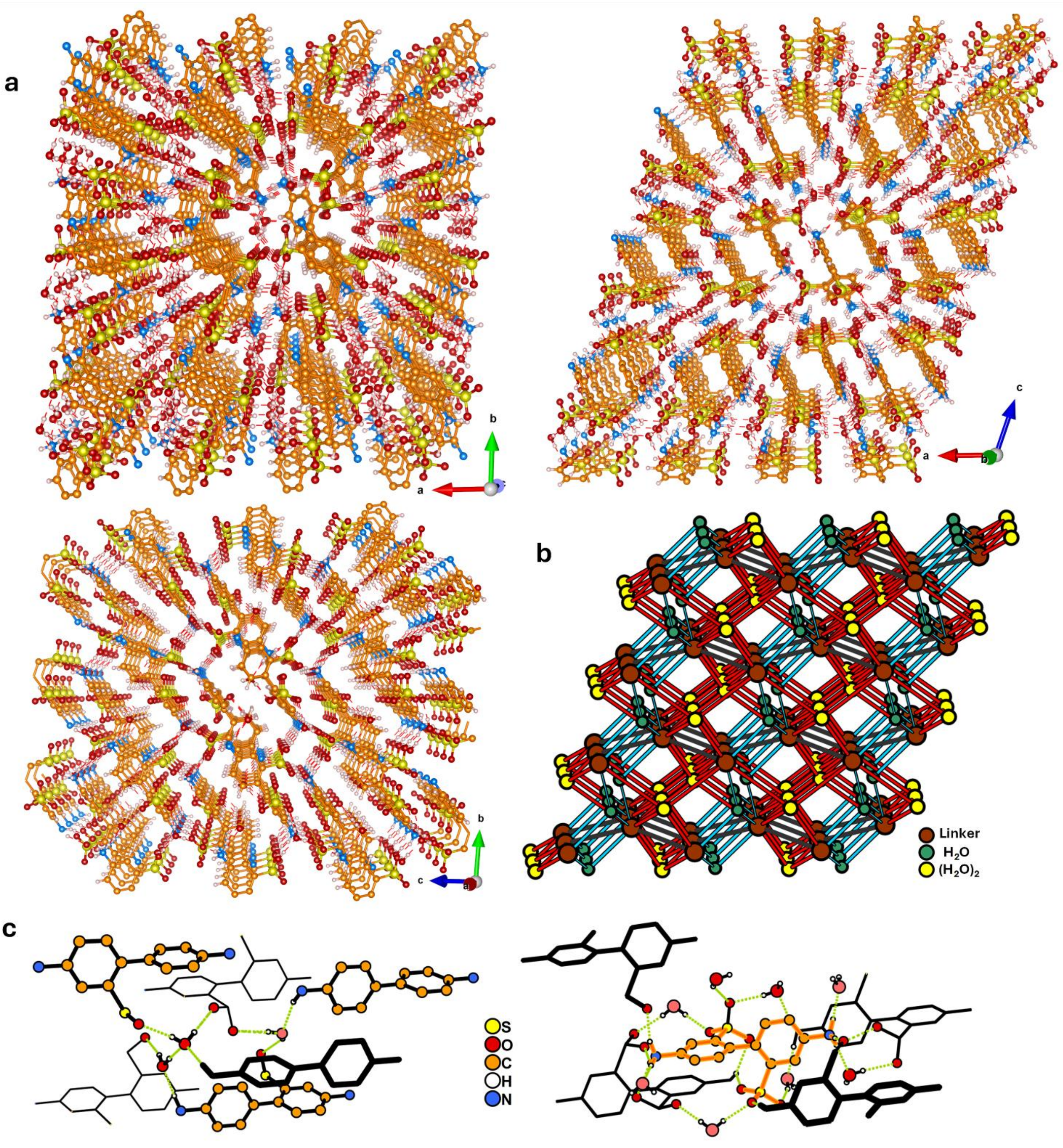

Figure S15. (a) Crystal structure of HOIF-301 in 3D view, color scheme: carbon, orange; oxygen, red; nitrogen, blue; sulfur, yellow; hydrogen, pink. (b) Topology of HOIF-301, a novel type of topology designated as ***oih***, with 3,5,12-c, 3-nodal net, point symbol: $\{3.4^2\}\{3^2.4^5.5^3\}\{3^6.4^{25}.5^{23}.6^{12}\}$. (c) Crystal structure representing hydrogen bonds and connectivity based on inorganic building blocks, water molecules, and organic zwitterions in HOIF-301. HOIF-301 presents a new structure with a space group of triclinic $P\overline{1}$. The organic components form hydrogen bonds mutually, which are further linked by water molecules to afford 3D hydrogen-bonding networks.

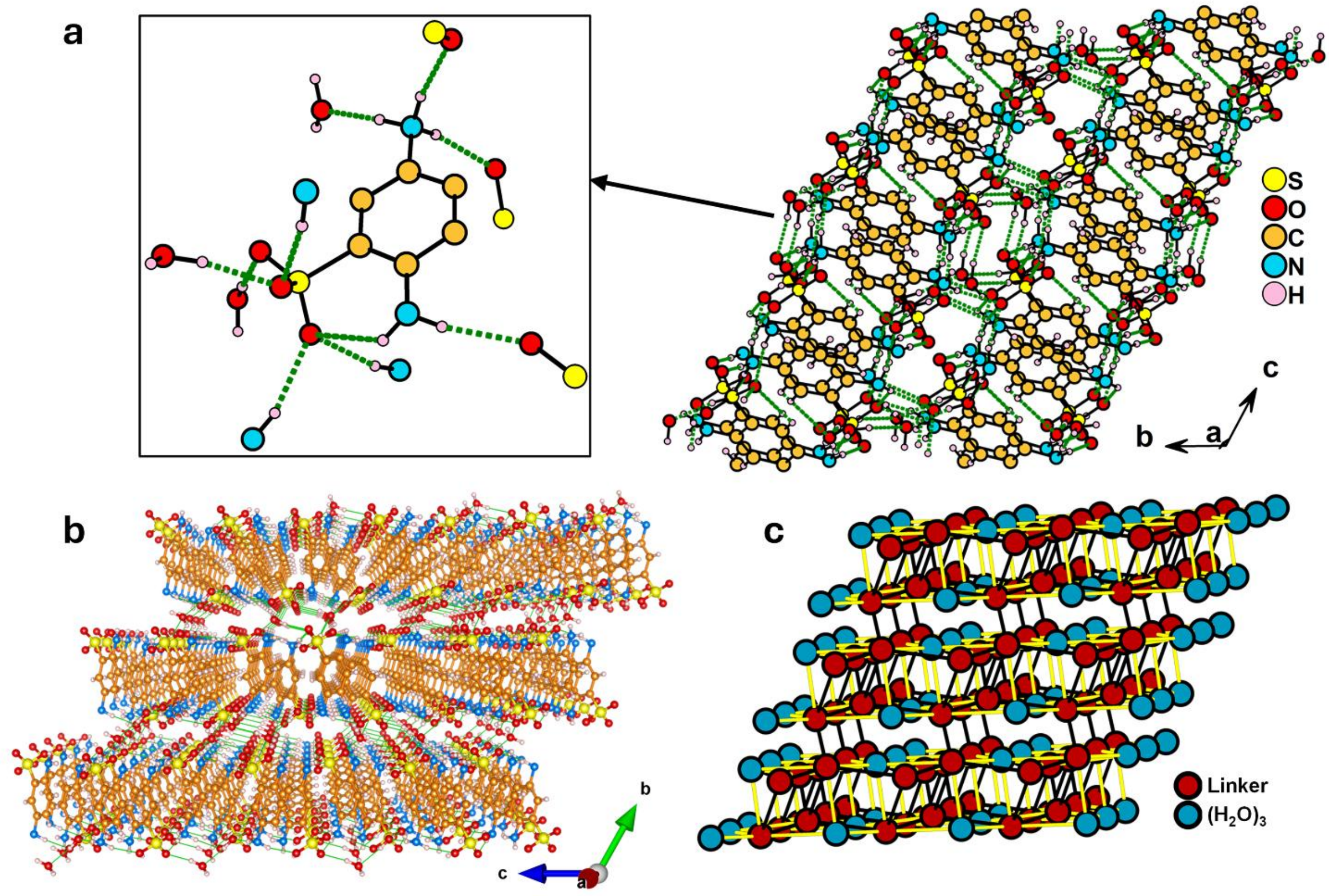

Figure S16. (a) Crystal structure representing hydrogen bonds and connectivity based on zwitterionic organic building blocks in HOIF-302. (a) Crystal structure of HOIF-302 in 3D view, color scheme: carbon, orange; oxygen, red; nitrogen, blue; sulfur, yellow; hydrogen, pink. (c) Topology of HOIF-302, a novel type of topology designated as ***das***, with 6,6,8-c, 3-nodal net, point symbol: $\{3^2.4^{10}.5^2.6\}\{3^2.4^{14}.5^5.6^7\}\{3^2.4^9.5^2.6^2\}$. HOIF-302 presents a new structure with a space group of triclinic $P\overline{1}$. The organic components form hydrogen bonds mutually, which further linked by water molecules to afford 2D hydrogen-bonding networks in *ac* plane.

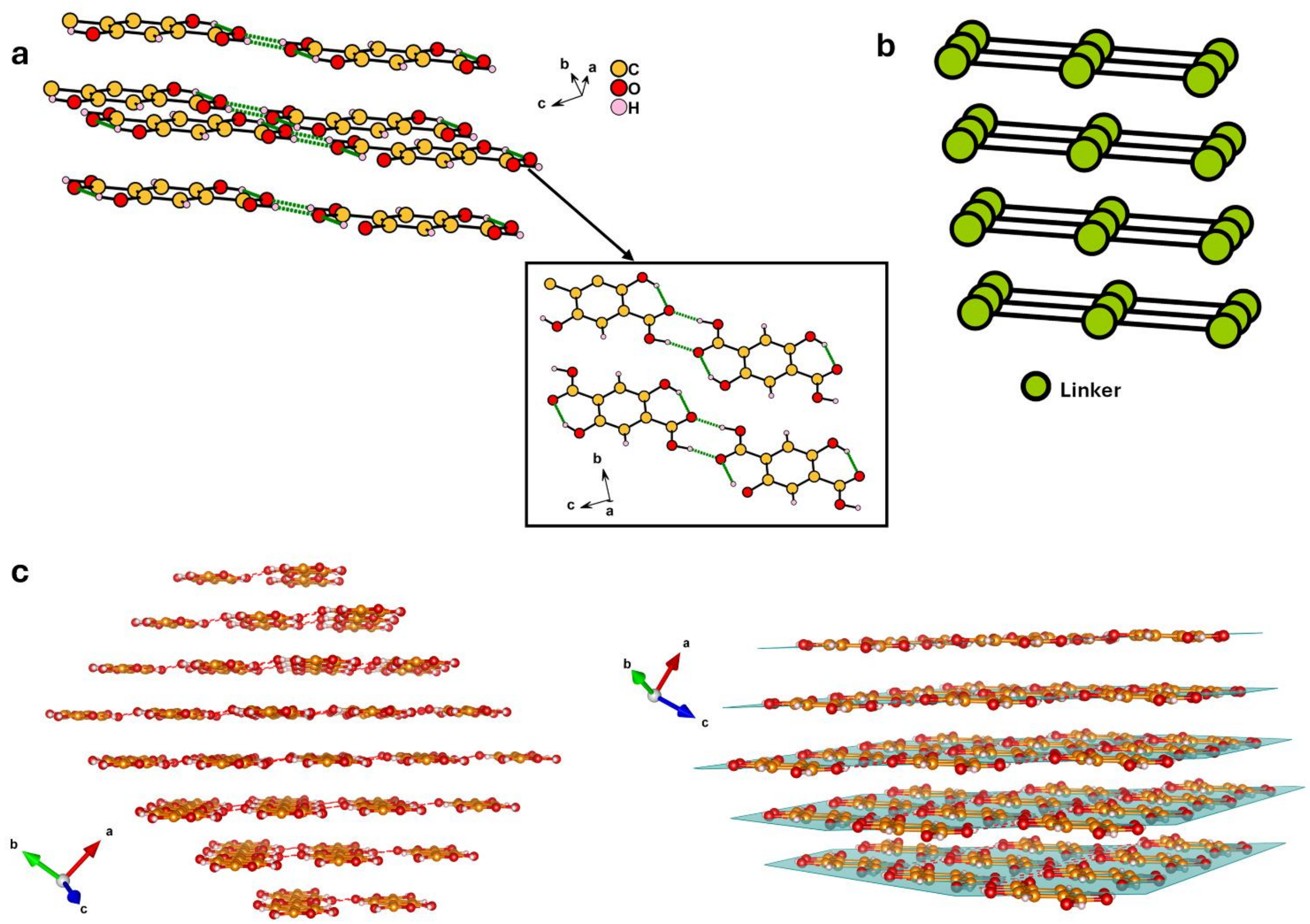

Figure S17. (a) Crystal structure representing hydrogen bonds and connectivity between organic building blocks in BDCDH. (b) Topology of BDCDH crystals, a novel type of topology designated as ***bdc***, with 2-c, uninodal net, point symbol: {0}. (c) Crystal structure of BDCDH in 3D view, color scheme: carbon, orange; oxygen, red; hydrogen, pink. Crystalline BDCDH presents a new structure with triclinic $P\bar{1}$ space group. The structure has been deposited to Cambridge Crystallographic Data Center (CCDC 2490186). The BDCDH molecules hydrogen-bonded with each other in a head-to-head mode to form a 1D molecular chain. The 1D chains are bridged by adjacent hydroxyl and carboxylate groups through relatively weak hydrogen bonding interaction, yielding 2D networks in $(11\bar{1})$ plane. The 2D networks further compile in an ABAB mode along the $[11\bar{1}]$ direction to afford the 3D structure.

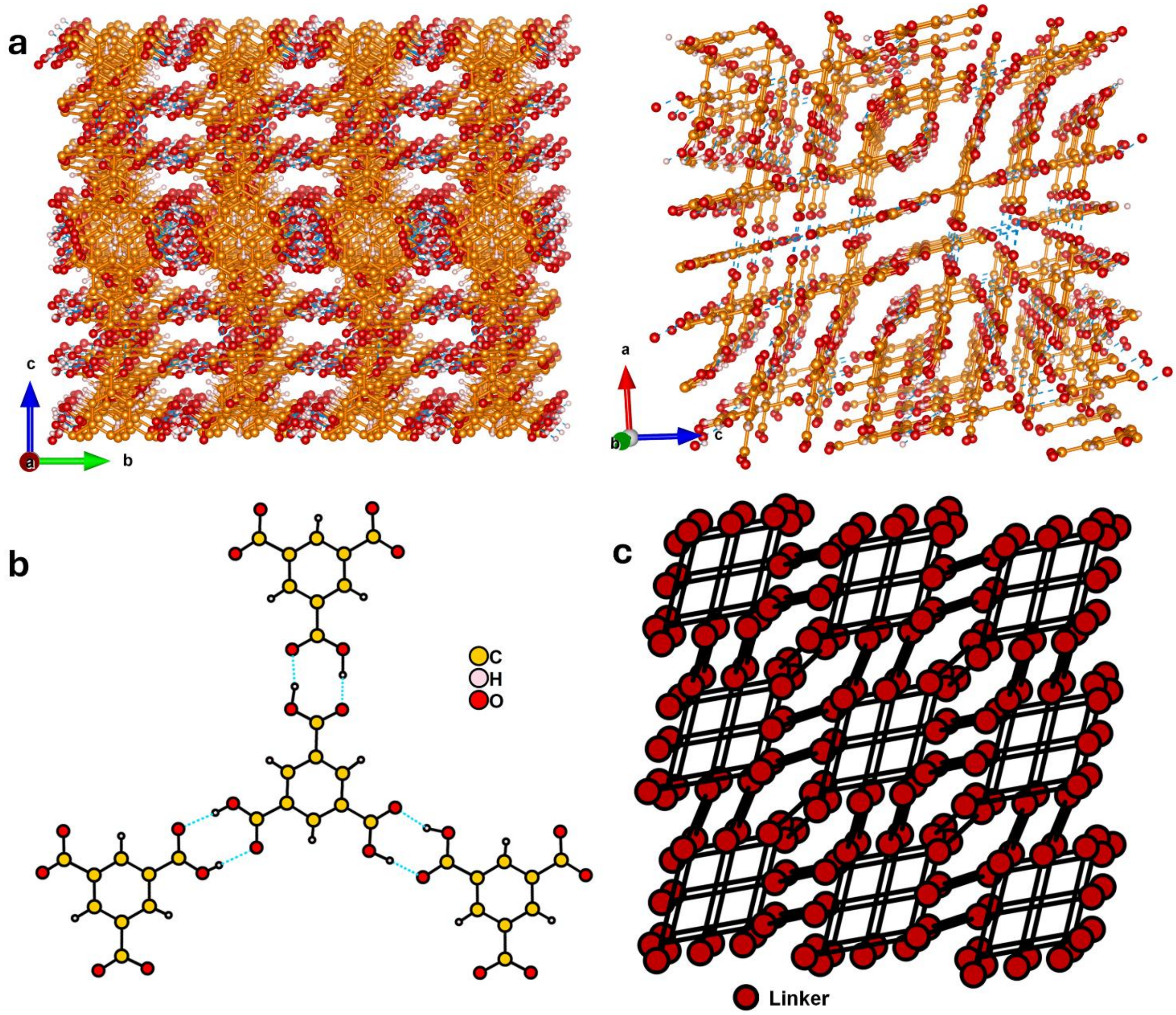

Figure S18. (a) Crystal structure of BTC in 3D view, color scheme: carbon, orange; oxygen, red; hydrogen, pink. (b) Crystal structure representing hydrogen bonds and connectivity between organic building blocks in BTC. (c) Topology of BTC crystals, which presents ***hcb*** type of topology, with 3-c, uninodal net, point symbol: $\{6^3\}$. BTC crystalizes in a space group of monoclinic *C*2/*c*.[19] The structure is constructed by discrete hydrogen bonds among BTC molecules, resulting in inconsecutive hydrogen-bonding networks.

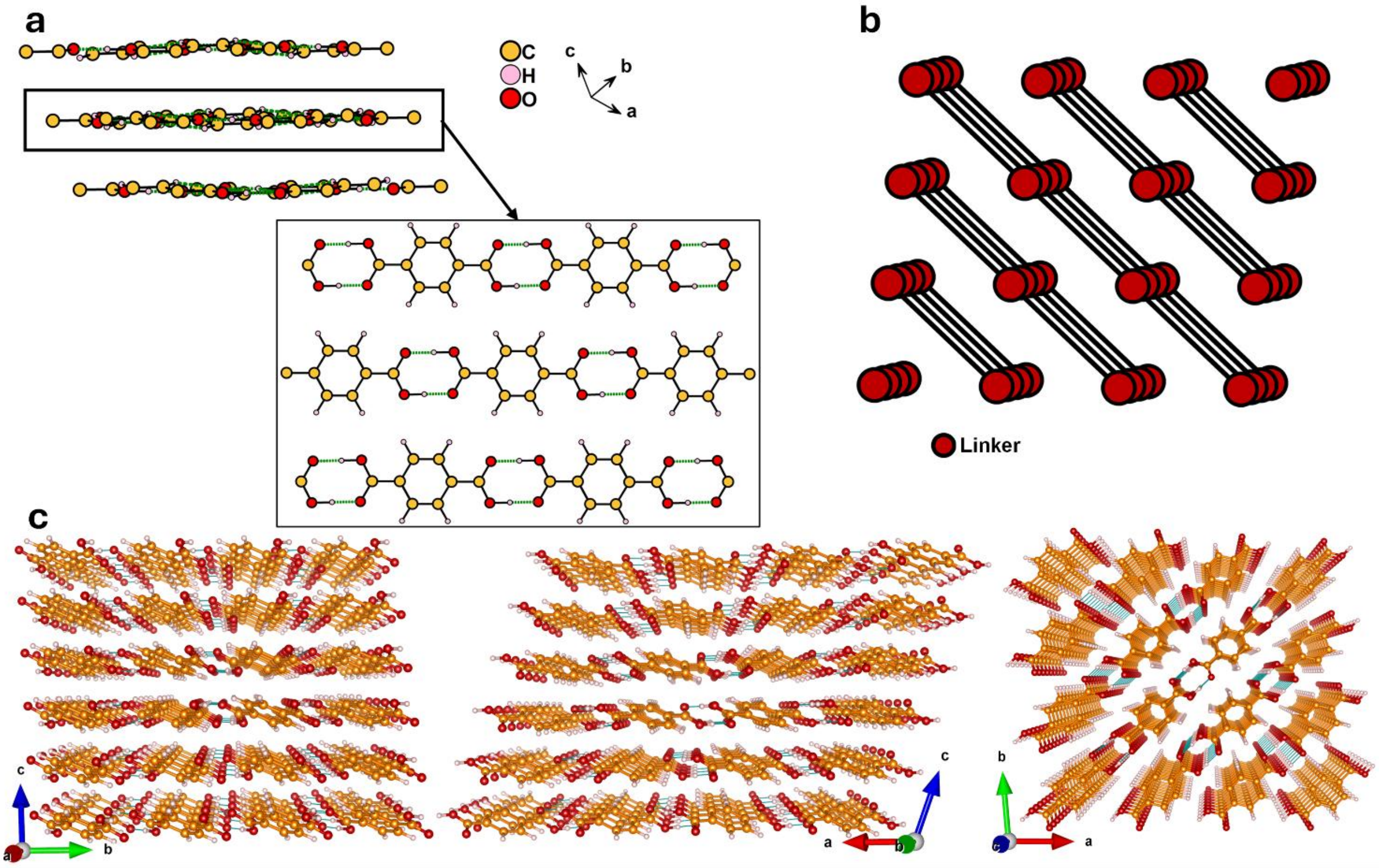

Figure S19. (a) Crystal structure representing hydrogen bonds and connectivity between organic building blocks in BDC. (b) Topology of BDC crystals, following the same topology as BDCDH, *i.e.*, ***bdc*** with 2-c uninodal net, point symbol: {0}. (c) Crystal structure of BDC in 3D view, color scheme: carbon, orange; oxygen, red; hydrogen, pink. BDC crystalizes in triclinic $P\bar{1}$ space group,[20] where BDC molecules are hydrogen-bonded with each other through carboxylate groups in a head-to-head manner, forming 1D chains that further align in the ab plane to yield a layered structure. Those hydrogen bonds formed are totally separated from each other, giving rise to a discontinuous hydrogen-bonding system.

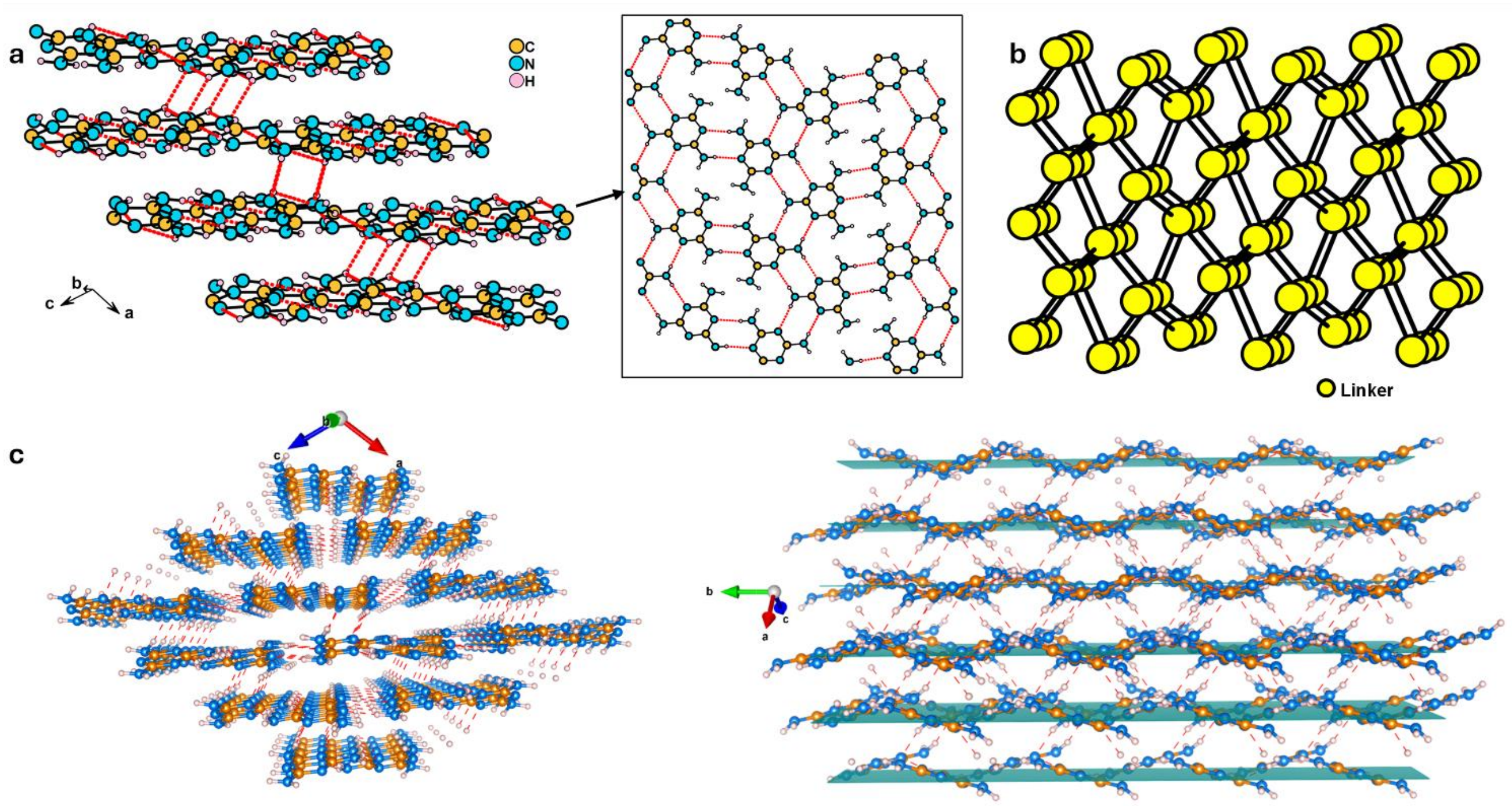

Figure S20. (a) Crystal structure representing hydrogen bonds and connectivity between organic building blocks in MA. (b) Topology of MA crystals, following ***dmp*** type of topology, with 4-c uninodal net, point symbol: $\{6^5.8\}$. (c) Crystal structure of MA in 3D view, color scheme: carbon, orange; nitrogen, blue; hydrogen, pink. MA crystalizes in monoclinic $P2_1/a$ space group (CCDC 2490187), where MA molecules are hydrogen-bonded mutually through amino groups to form 2D hydrogen-bonding networks in (201) plane. The 2D hydrogen-bonded layers adopt a corrugated topology, in which the molecules are arranged in an alternating tilted fashion, giving rise to a zigzag-like, twisted sheet architecture.

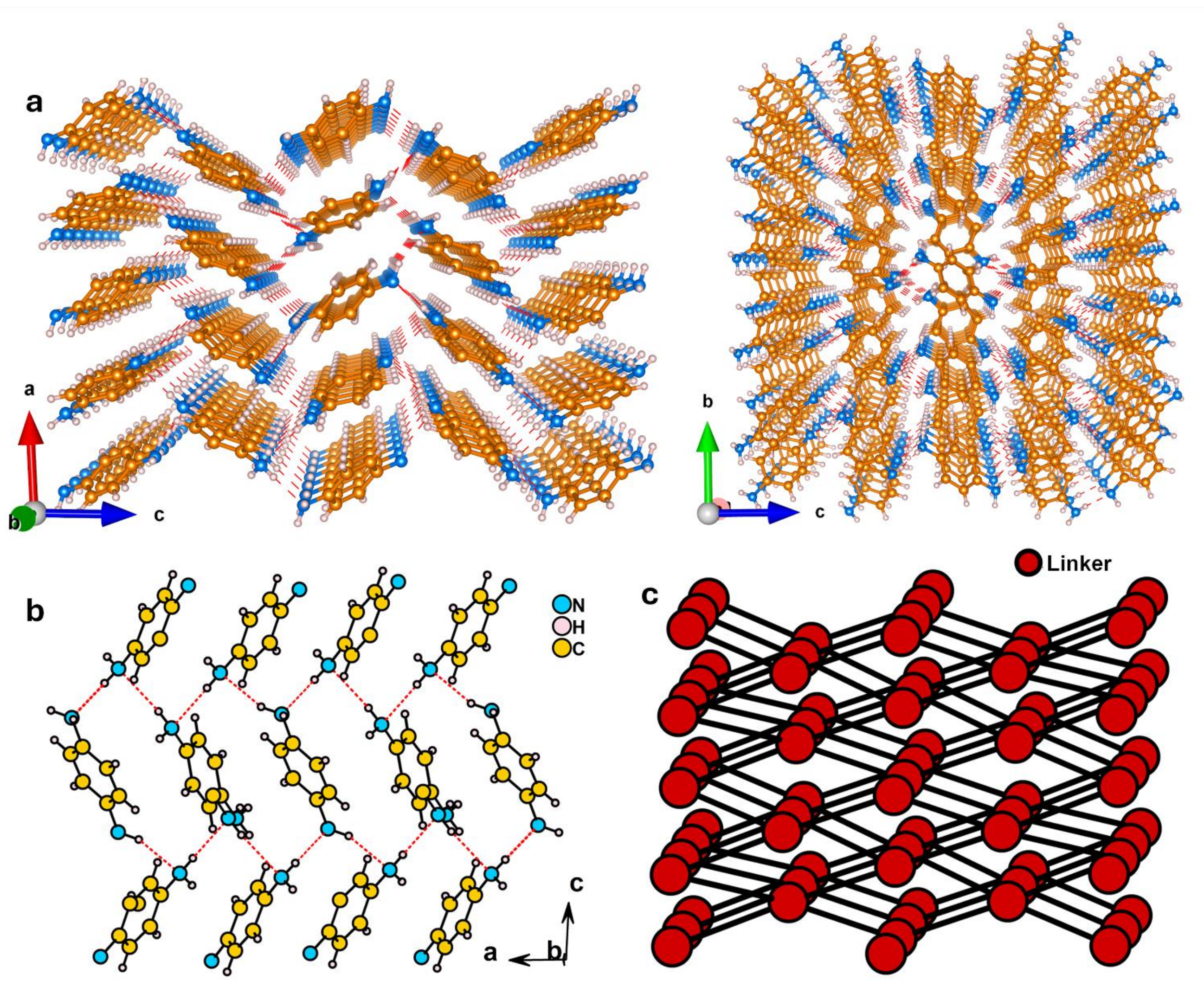

Figure S21. (a) Crystal structure of BDA in 3D view, color scheme: carbon, orange; nitrogen, blue; hydrogen, pink. (b) Crystal structure representing hydrogen bonds and connectivity between organic building blocks in BDA. (c) Topology of BDA crystals, following ***cds*** type of topology, with 4-c uninodal net, point symbol: $\{6^5.8\}$. BDA crystalizes in monoclinic $P2_1/c$ space group,[21] where BDA molecules are hydrogen-bonded with each other through amino groups, forming zigzag-like layers along *a*-axis. The protruding amino groups in the interspace connected mutually through hydrogen bonds to bridge neighboring layers, yielding the final 3D structure. In addition, there are 1D zigzag-like hydrogen-bonding networks running through the structure along *a*-axis.

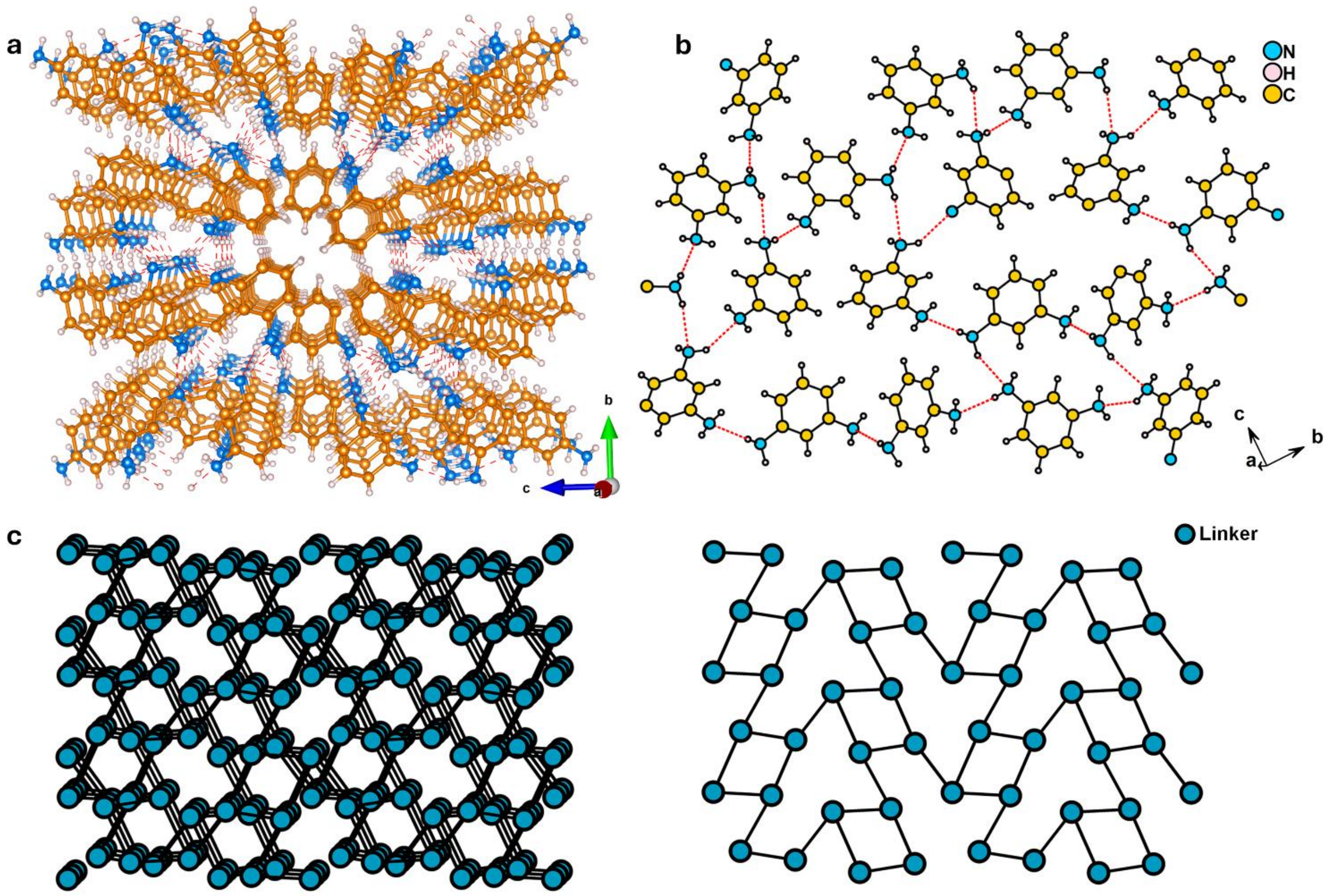

Figure S22. (a) Crystal structure of mBDA in 3D view, color scheme: carbon, orange; nitrogen, blue; hydrogen, pink. (b) Crystal structure representing hydrogen bonds and connectivity between organic building blocks in mBDA. (c) Topology of mBDA crystals, following ***fes*** type of topology, with 3-c uninodal net, point symbol: $\{4.8^2\}$. mBDA crystalizes in monoclinic $P2_1/c$ space group,[22] where mBDA molecules are hydrogen-bonded with each other through amino groups in a 3D manner. However, the hydrogen-bonding networks are generated only in short-range and isolate with each other. Lacking continuous hydrogen-bonding networks accounts for inferior proton conducting performance.

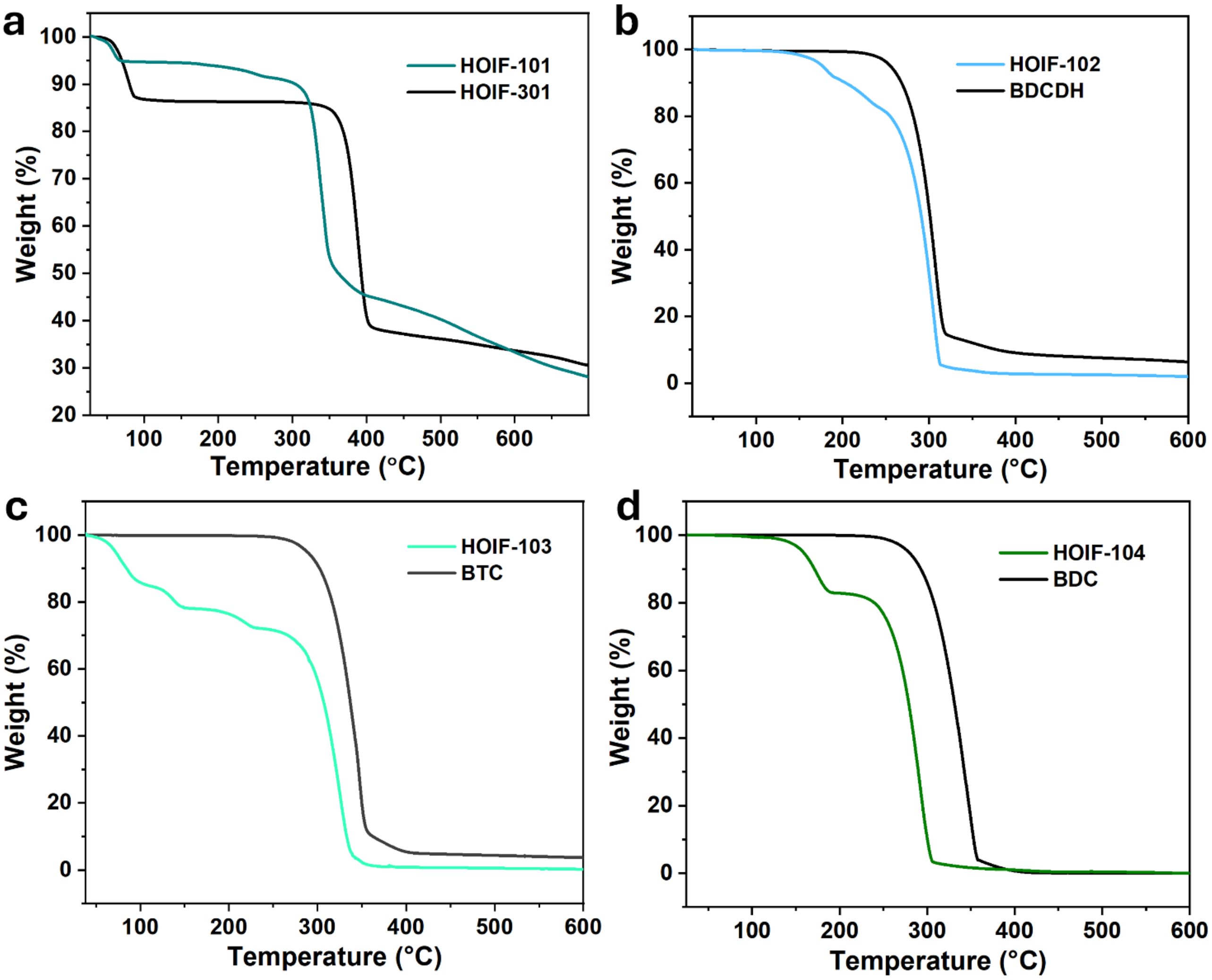

Figure S23. Thermogravimetric analysis (TGA) profiles of (a) HOIF-101 and HOIF-301, (b) HOIF-102, (c) HOIF-103, and (d) HOIF-104, along with their parent compounds, measured under a nitrogen atmosphere in the temperature range from room temperature to 600 °C.

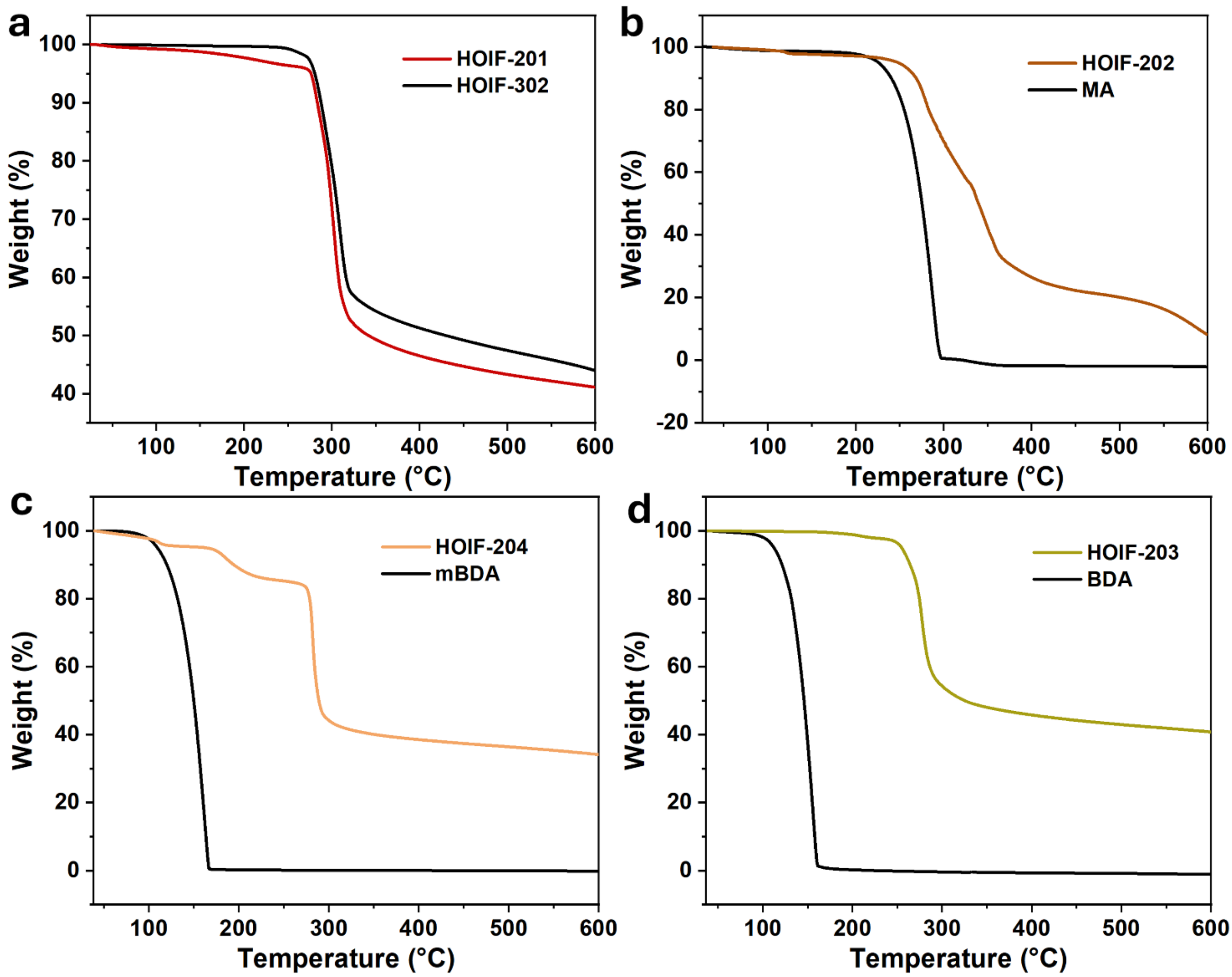

Figure S24. TGA profiles of (a) HOIF-201 and HOIF-302, (b) HOIF-202, (c) HOIF-203, and (d) HOIF-204, along with their parent compounds, measured under a nitrogen atmosphere in the temperature range from room temperature to 600 °C.

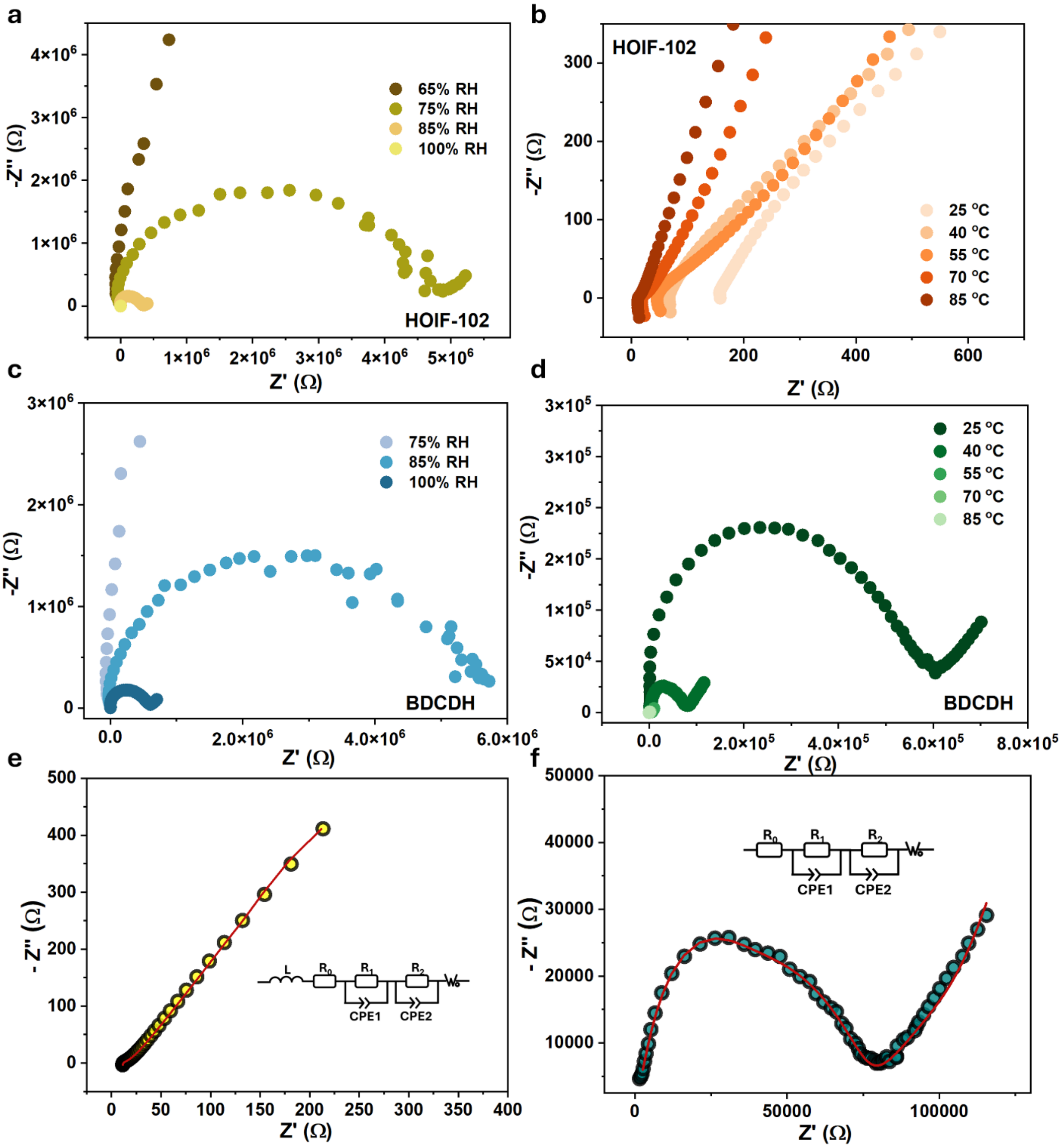

Figure S25. (a) Nyquist plots of HOIF-102 under various relative humidity conditions at room temperature. (b) Nyquist plots of HOIF-102 under different temperatures at 100% RH. (c) Nyquist plots of the parent counterpart, BDCDH, under different RH conditions at room temperature. (d) Nyquist plots of BDCDH at different temperatures under 100% RH. (e) Fitting of the HOIF-102 Nyquist plot using the proposed equivalent circuit (85 °C, 100% RH). (f) Nyquist plot of DAS fitted with the proposed equivalent circuit (40 °C, 100% RH).

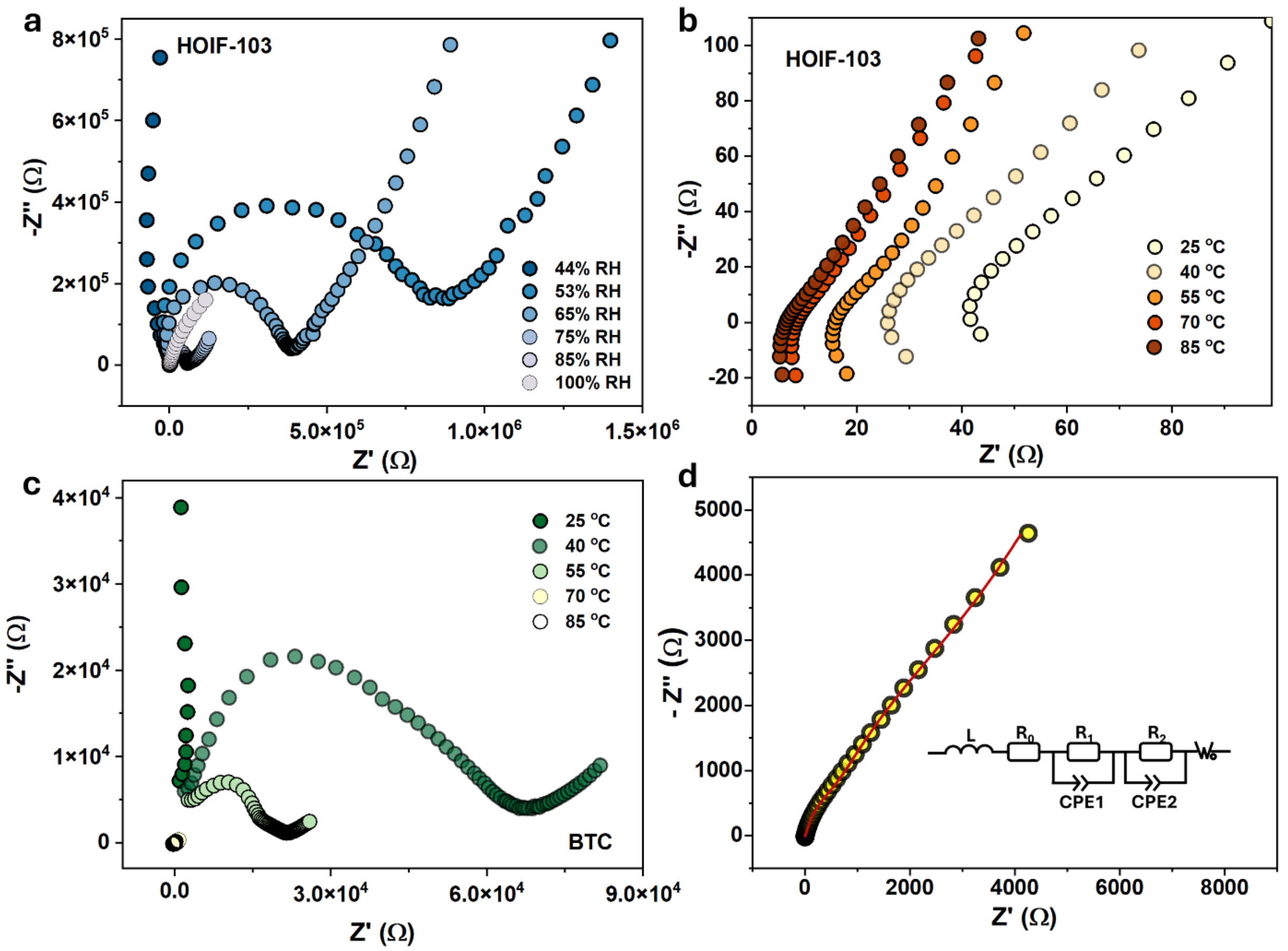

Figure S26. (a) Nyquist plots of HOIF-103 under various relative humidity conditions at room temperature. (b) Nyquist plots of HOIF-103 under different temperatures at 100% RH. (c) Nyquist plots of BTC at different temperatures under 100% RH. (d) Equivalent circuit fitting of Nyquist plot measured at 85 °C, 100% RH for HOIF-103. Note that the Nyquist plots of BTC at low humidity could not be measured due to its high resistance, while those of BDCDH were measurable at low humidity probably owing to the formation of continuous hydrogen-bonding network.

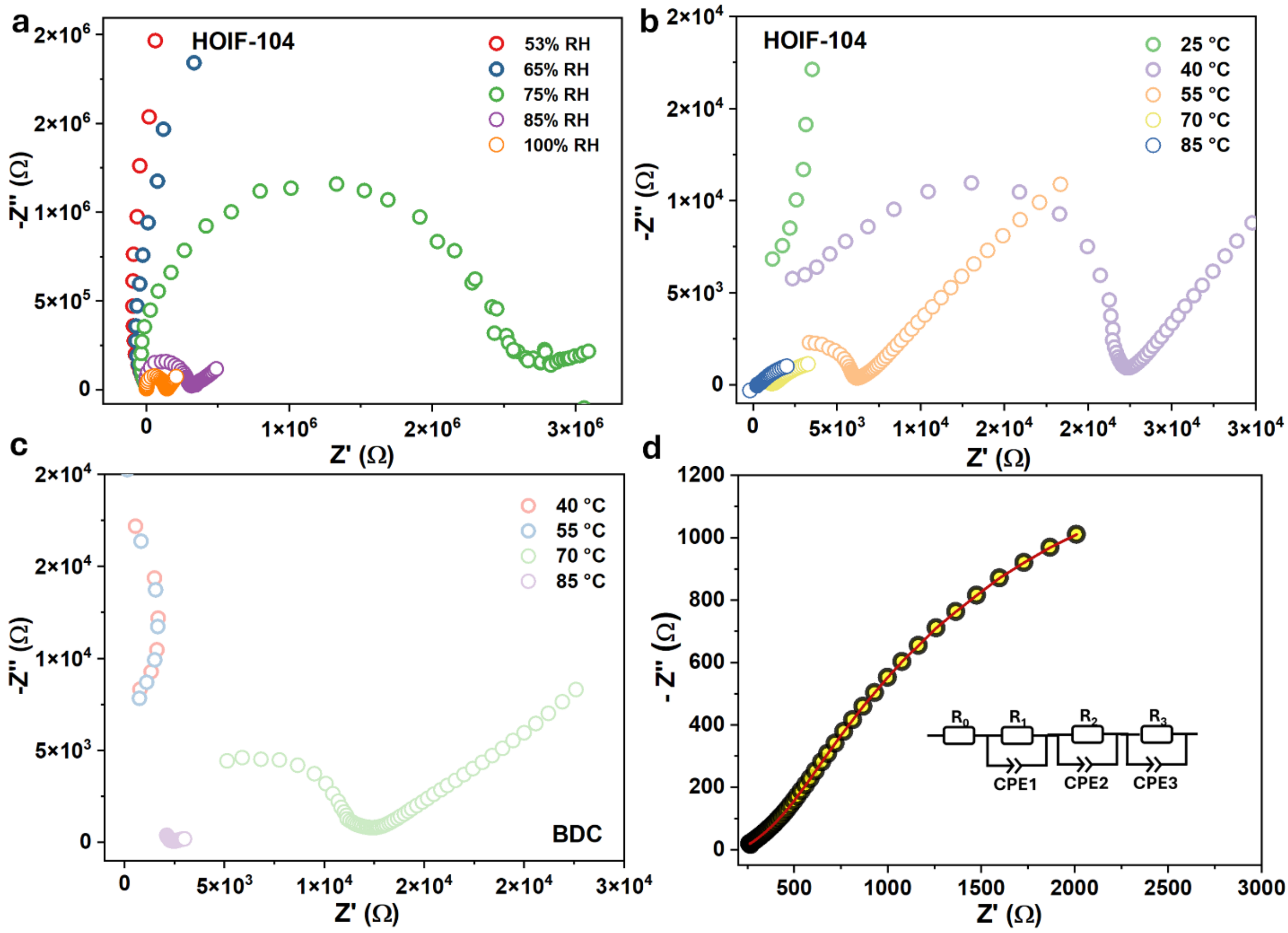

Figure S27. Nyquist plots of HOIF-104 under various relative humidity conditions at room temperature. (b) Nyquist plots of HOIF-104 under different temperatures at 100% RH. (c) Nyquist plots of BDC at different temperatures under 100% RH. (d) Equivalent circuit fitting of Nyquist plot measured at 85 °C, 100% RH for HOIF-104. Note that the Nyquist plots of BTC at low humidity as well as at 85 °C and 100% RH could not be measured due to its high resistance.

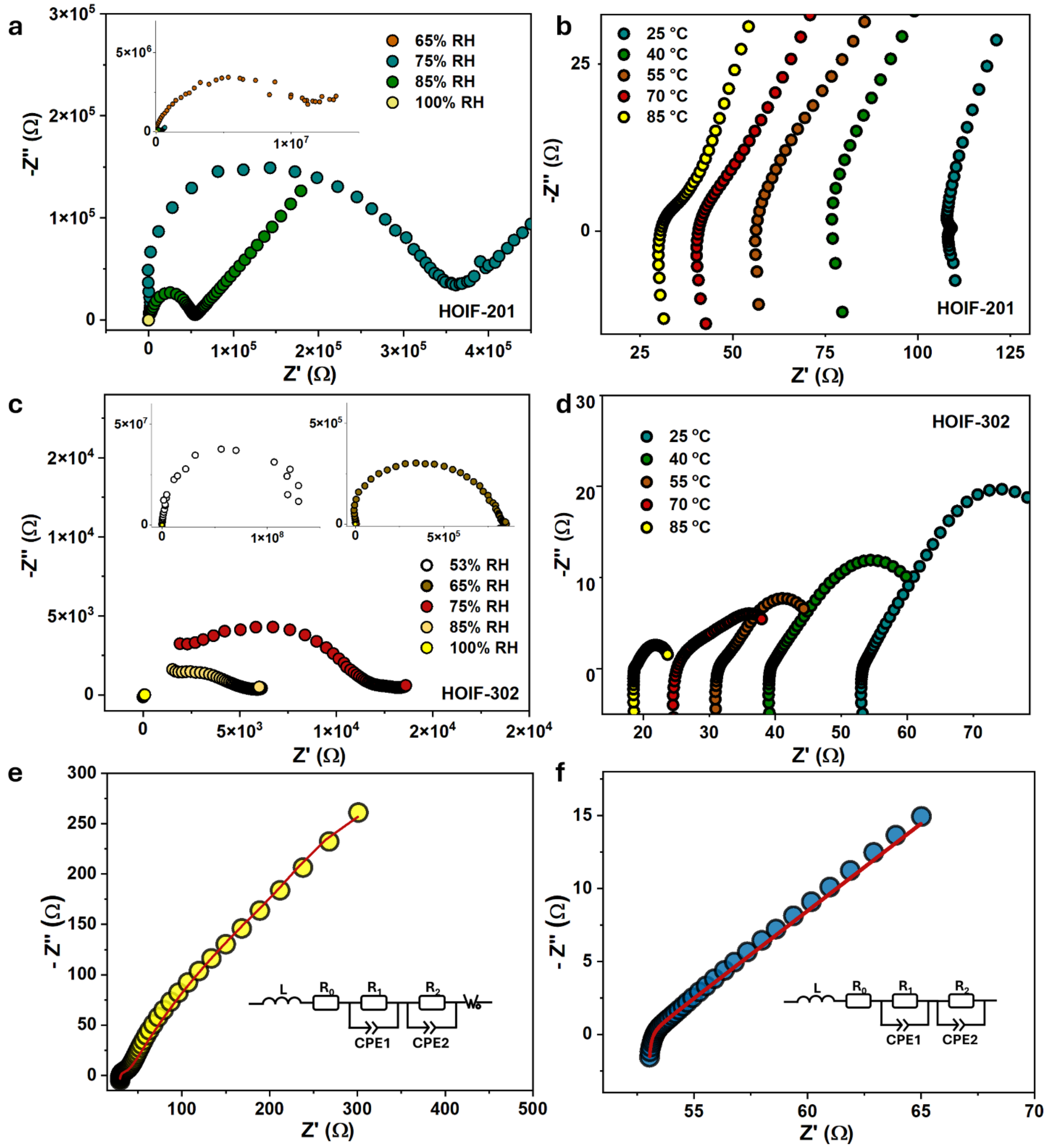

Figure S28. (a) Nyquist plots of HOIF-201 under various relative humidity conditions at room temperature. (b) Nyquist plots of HOIF-201 under different temperatures at 100% RH. (c) Nyquist plots of HOIF-302 under different RH conditions at room temperature. (d) Nyquist plots of HOIF-302 at different temperatures under 100% RH. (e) Fitting of the HOIF-201 Nyquist plot using the proposed equivalent circuit (85 °C, 100% RH). (f) Nyquist plot of DAS fitted with the proposed equivalent circuit (25 °C, 100% RH).

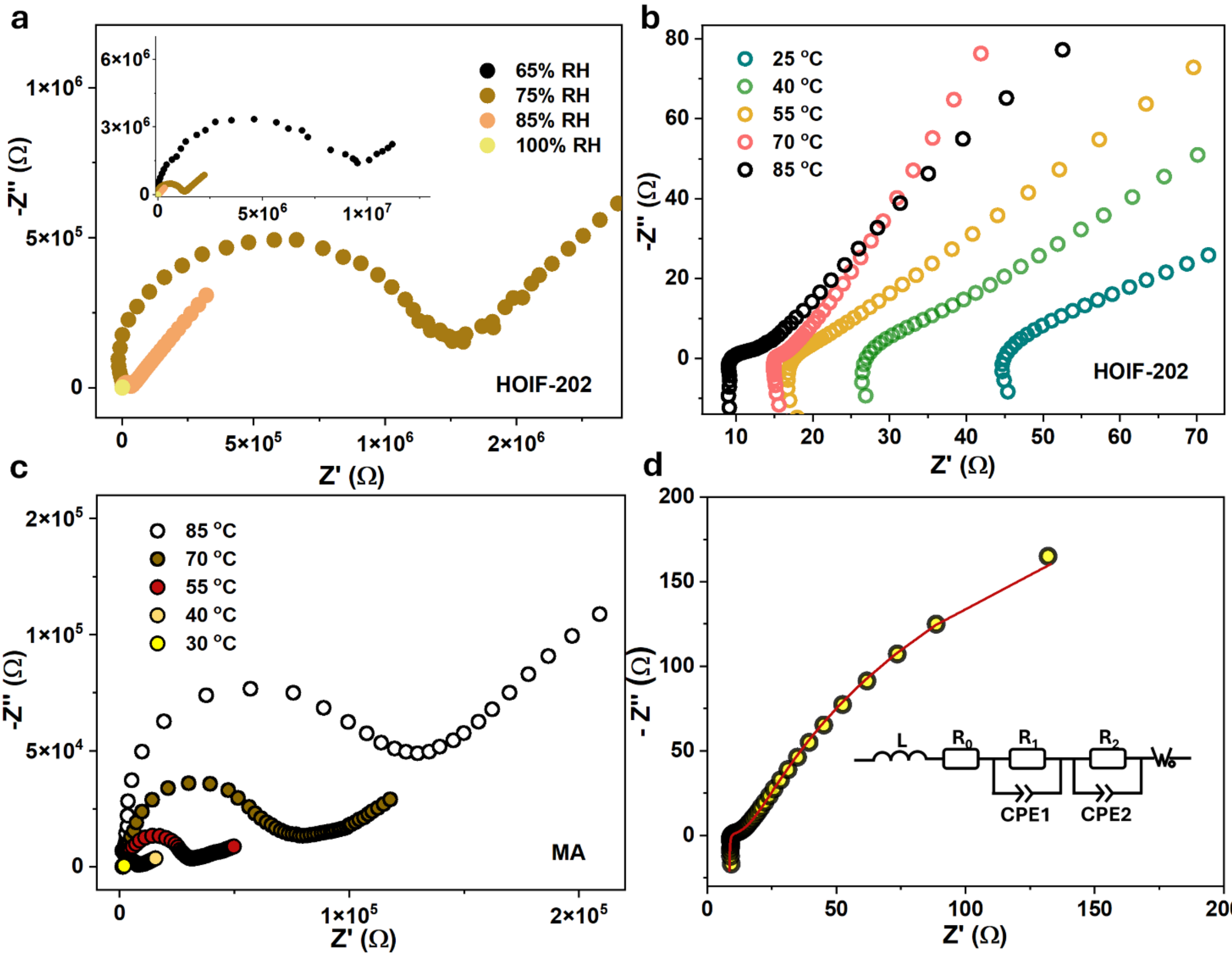

Figure S29. Nyquist plots of HOIF-202 under various relative humidity conditions at room temperature. (b) Nyquist plots of HOIF-202 under different temperatures at 100% RH. (c) Nyquist plots of MA at different temperatures under 100% RH. (d) Equivalent circuit fitting of Nyquist plot measured at 85 °C, 100% RH for HOIF-202. Note that the Nyquist plots of MA at low humidity could not be measured due to its high resistance.

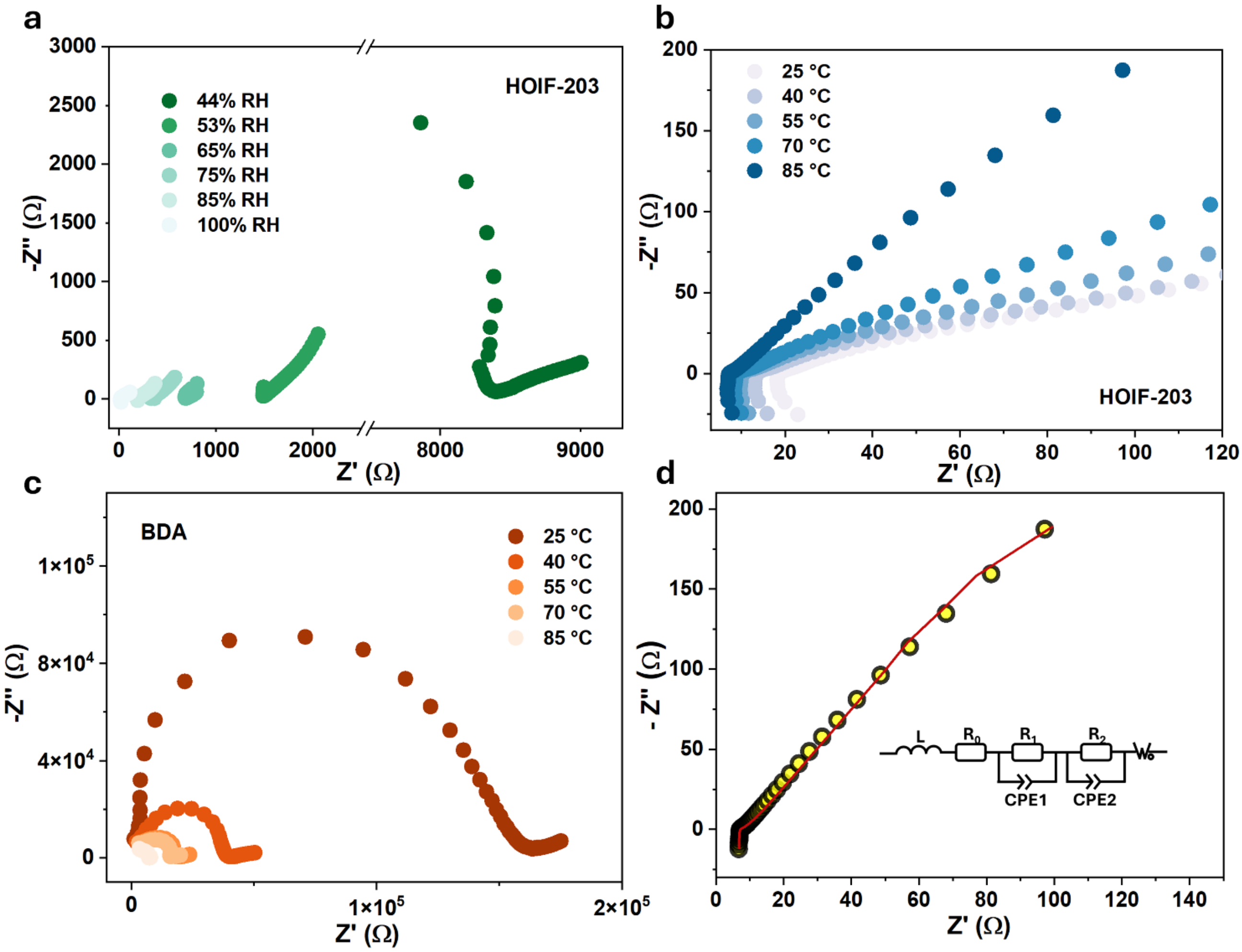

Figure S30. Nyquist plots of HOIF-203 under various relative humidity conditions at room temperature. (b) Nyquist plots of HOIF-203 under different temperatures at 100% RH. (c) Nyquist plots of BDA at different temperatures under 100% RH. (d) Equivalent circuit fitting of Nyquist plot measured at 85 °C, 100% RH for HOIF-203. Note that the Nyquist plots of BDA at low humidity could not be measured due to its high resistance.

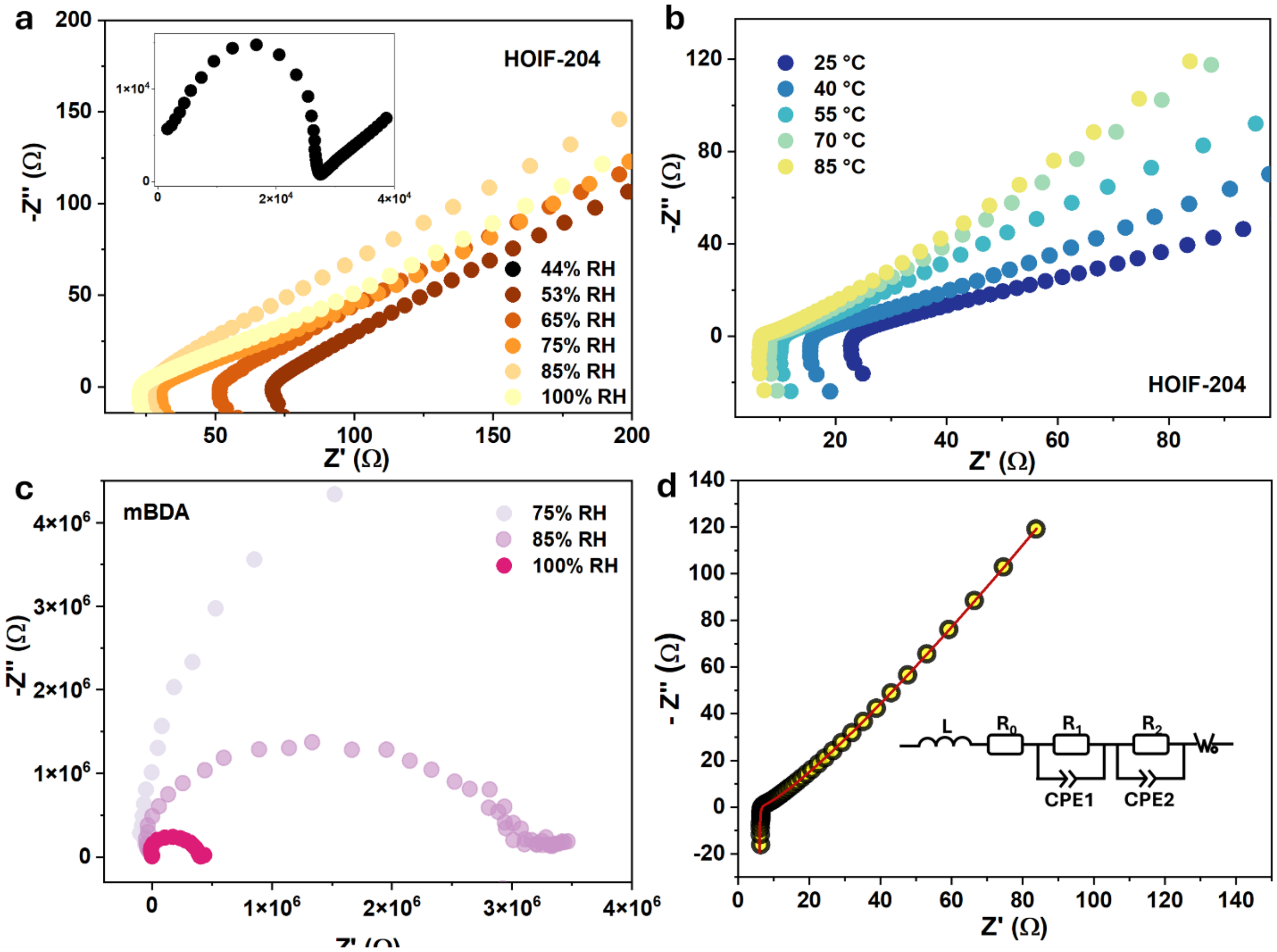

Figure S31. Nyquist plots of HOIF-204 under various relative humidity conditions at room temperature. (b) Nyquist plots of HOIF-204 under different temperatures at 100% RH. (c) Nyquist plots of mBDA at different RHs and room temperature. (d) Equivalent circuit fitting of Nyquist plot measured at 85 °C, 100% RH for HOIF-204. Note that the Nyquist plots of mBDA at high temperature could not be measured due to sample degradation.

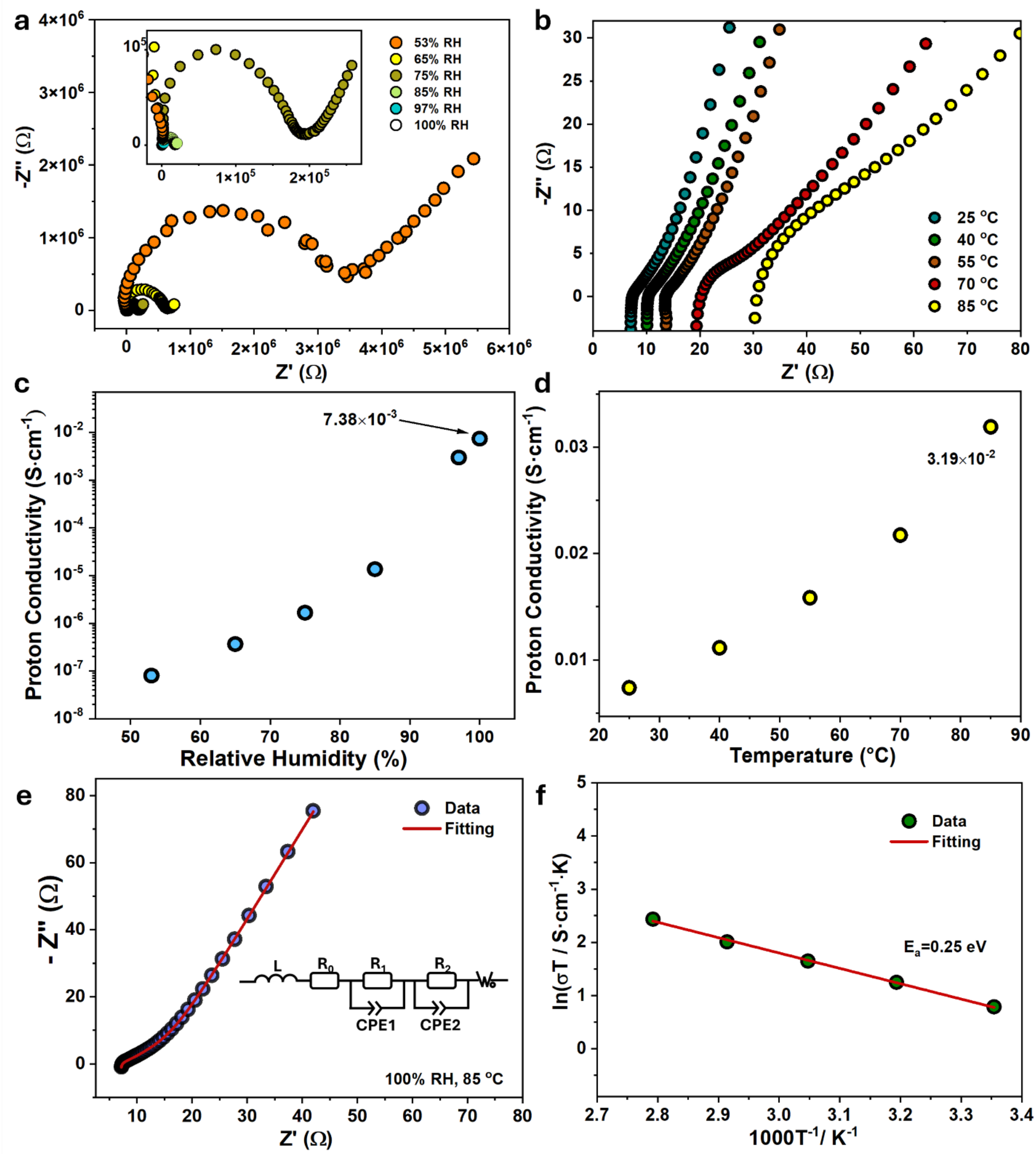

Figure S32. Impedance analysis for HOIF-101: (a) Nyquist plots recorded under different RH conditions at room temperature; (b) Nyquist plots recorded at different temperatures under 100% RH; (c) humidity-dependence of proton conductivity at room temperature. (d) temperature-dependence of proton conductivity at 100% RH; (e) an example of employing the proposed equivalent circuit for fitting the experimental data measured at 100% RH and 85 °C; (f) the *Arrhenius* analysis and derived activation energy.

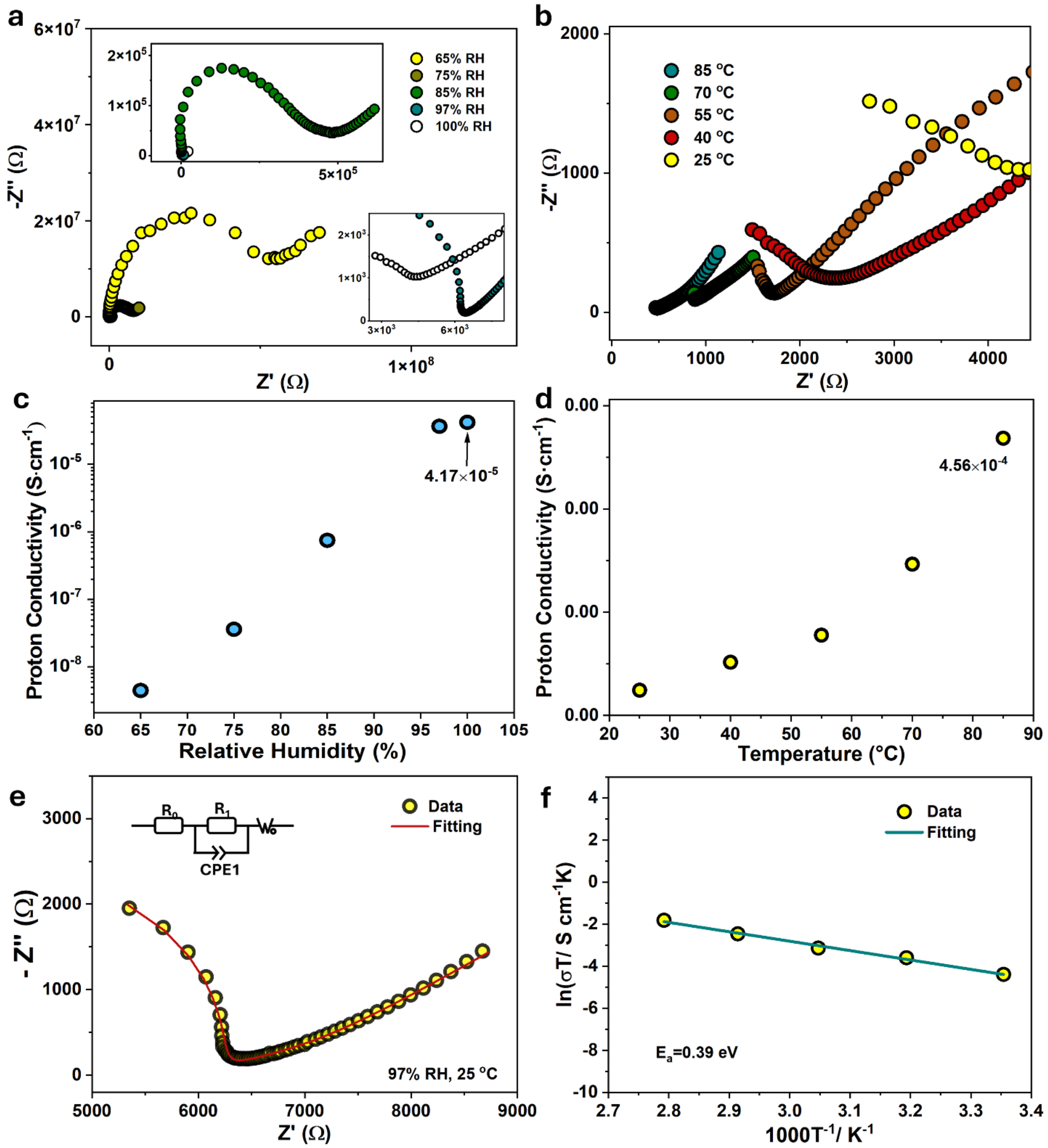

Figure S33. Impedance analysis for HOIF-301: (a) Nyquist plots measured under different RH at room temperature; (b) Nyquist plots measured at different temperatures under 100% RH; (c) proton conductivity as a function of humidity at room temperature. (d) proton conductivity as a function of temperature at 100% RH; (e) an example of employing the proposed equivalent circuit for fitting the experimental data measured at 97% RH and 25 °C; (f) the *Arrhenius* analysis and derived activation energy.

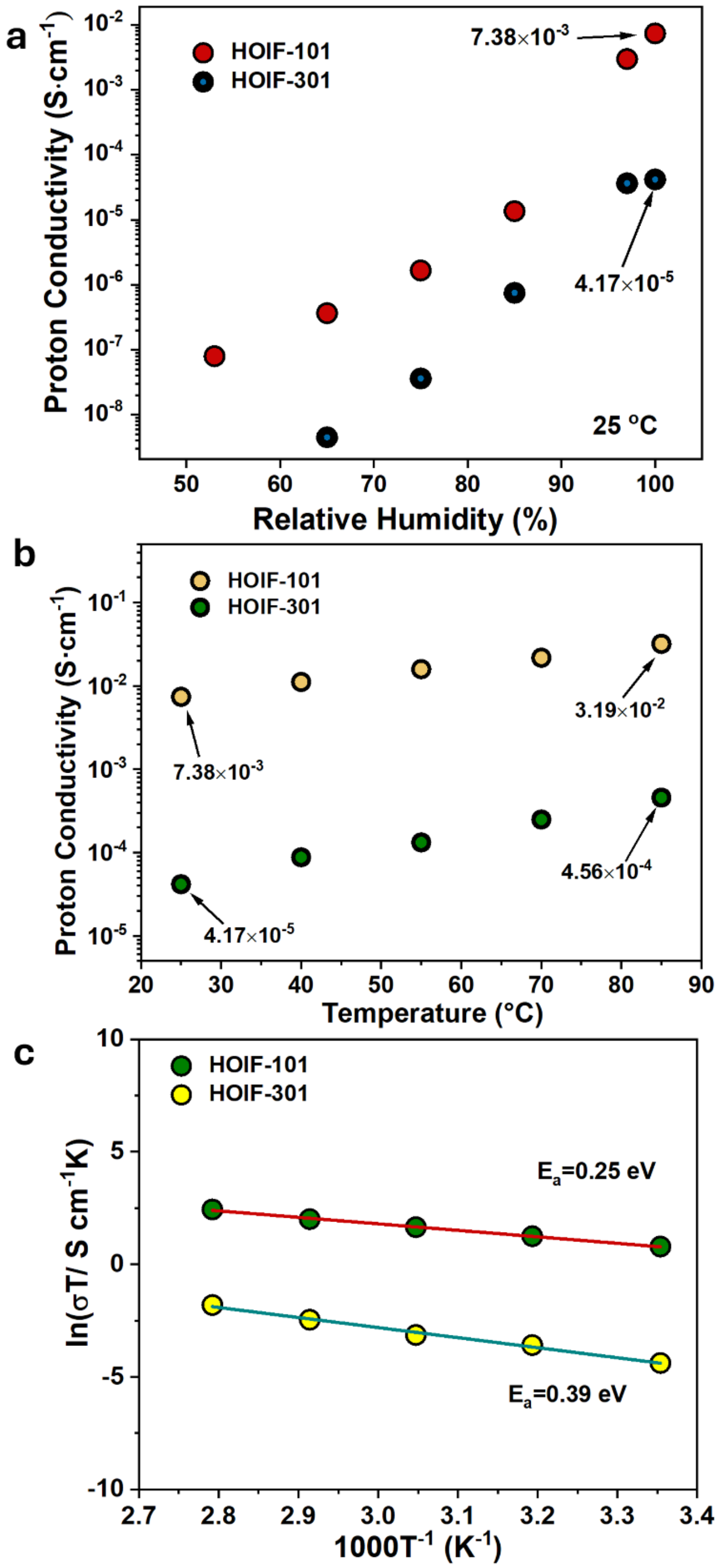

Figure S34. (a) Proton conductivity of HOIF-101 and HOIF-301 as a function of RH at room temperature. (b) Proton conductivity of HOIF-101 and HOIF-301 as a function of temperature under 100% RH. (c) The *Arrhenius* plots and related activation energy of HOIF-101 and HOIF-301.

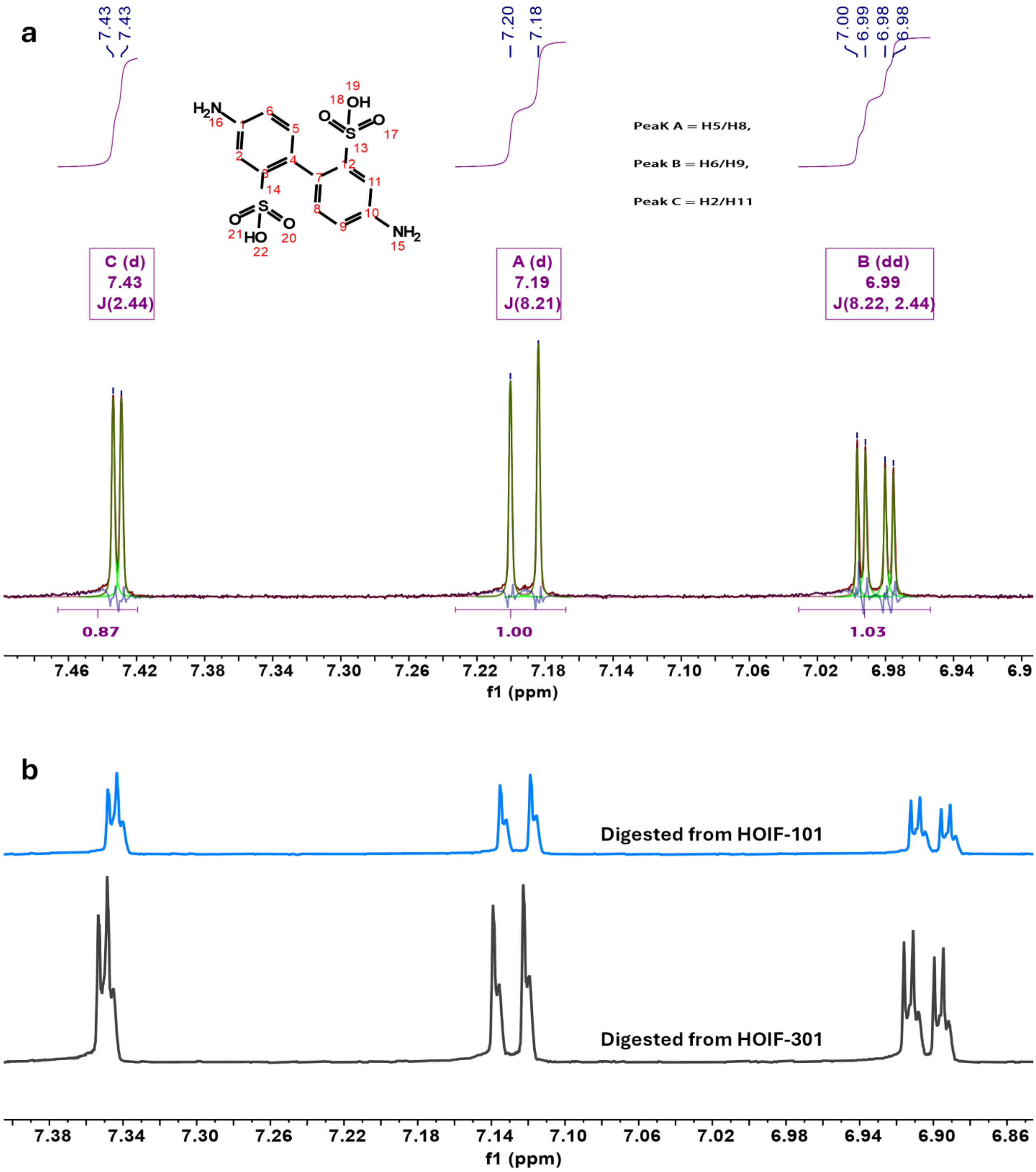

Figure S35. ^{1}H NMR spectra of (a) HOIF-101 in deuterated water (D_2O), (b) HOIF-101 and HOIF-301 digested in sodium deuteroxide solution in D_2O.

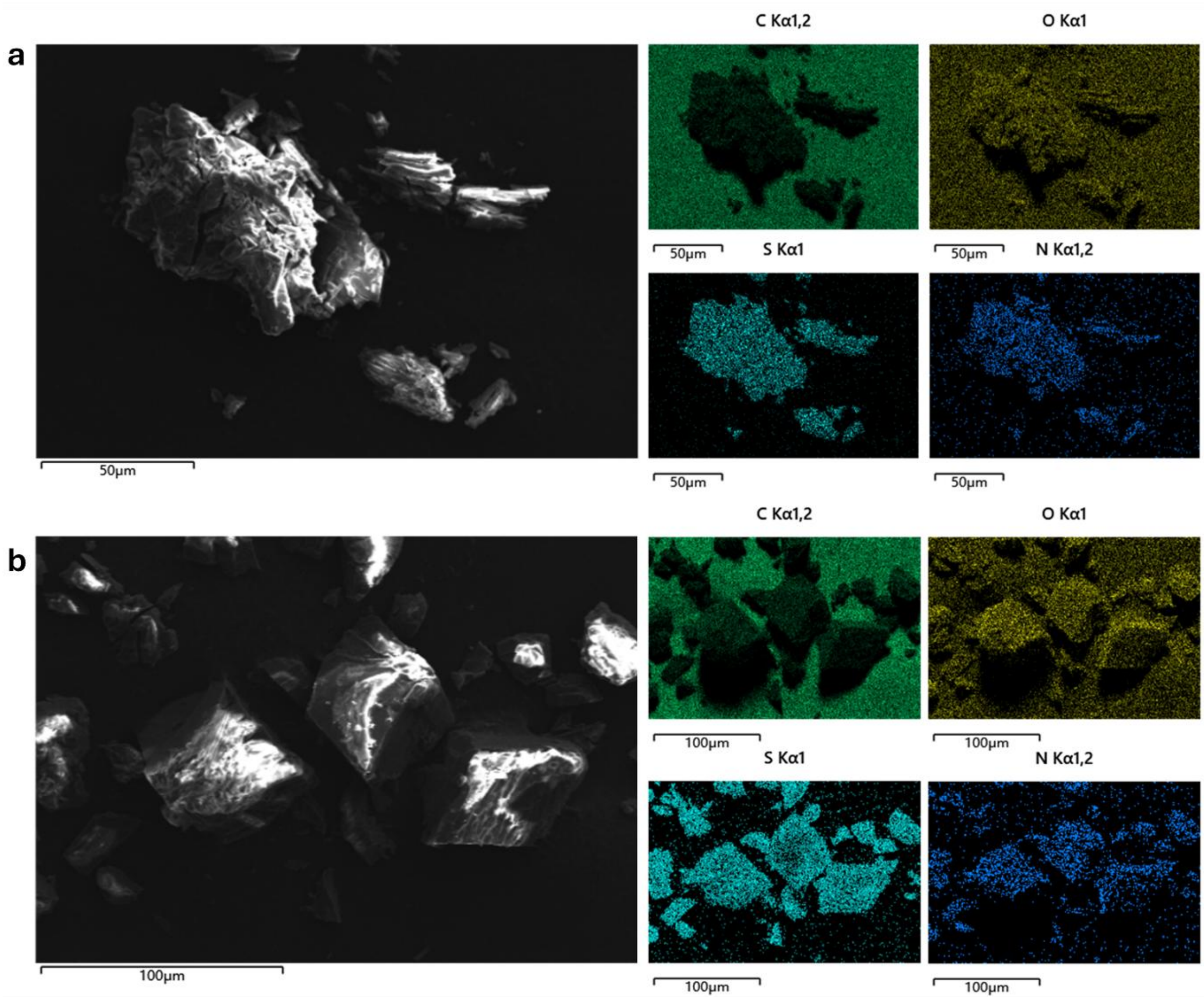

Figure S36. SEM images and EDX mapping of carbon, nitrogen, oxygen and sulfur for (a) HOIF-301 with scale bar of 50 µm and (b) HOIF-101 with scale bar of 100 µm.

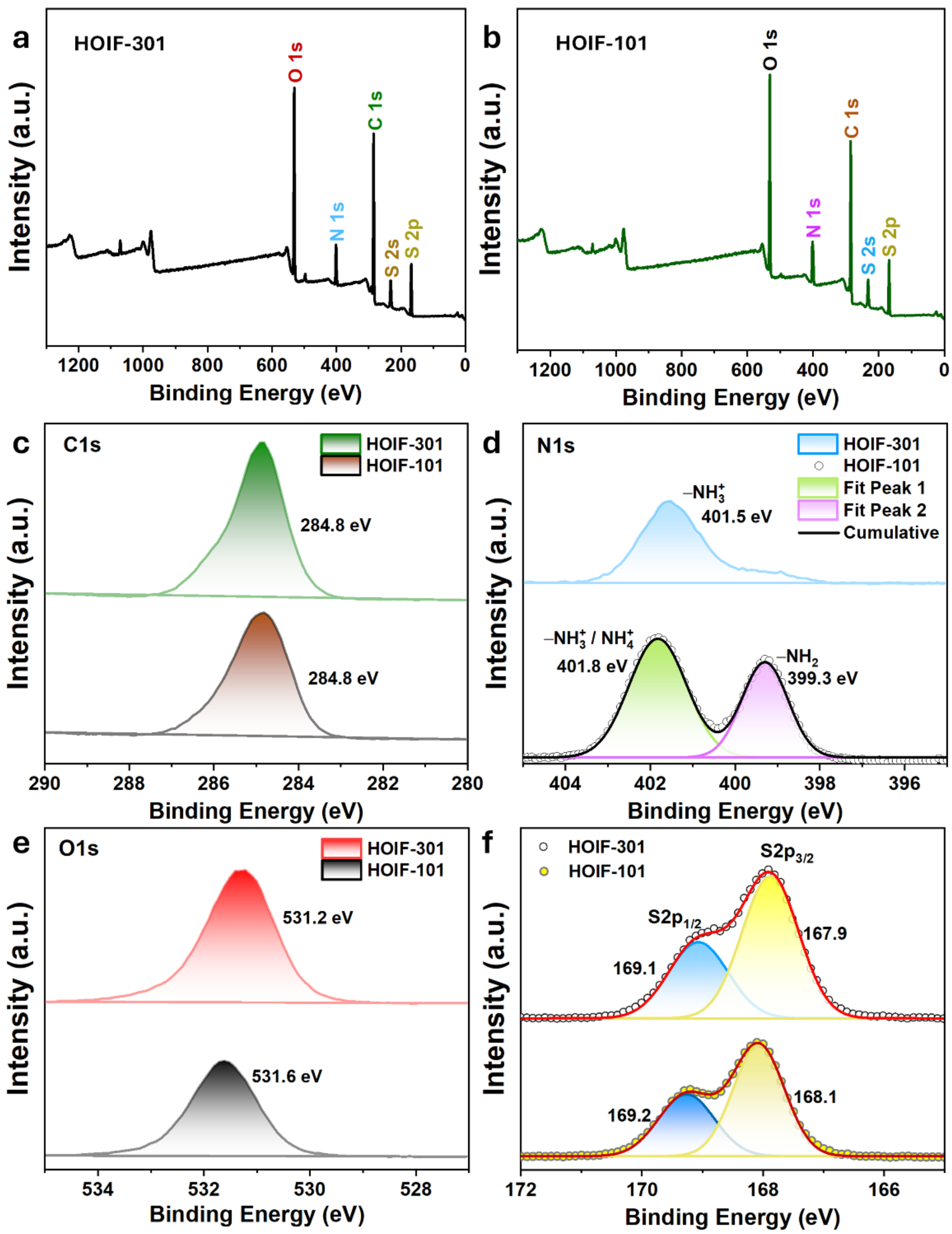

Figure S37. XPS spectra of HOIF-301 and HOIF-101. Survey spectra of (a) HOIF-301 and (b) HOIF-101. High-resolution XPS spectra of (c) C 1s, (d) N 1s, (e) O 1s, and (f) S 2p regions for HOIF-301 and HOIF-101.

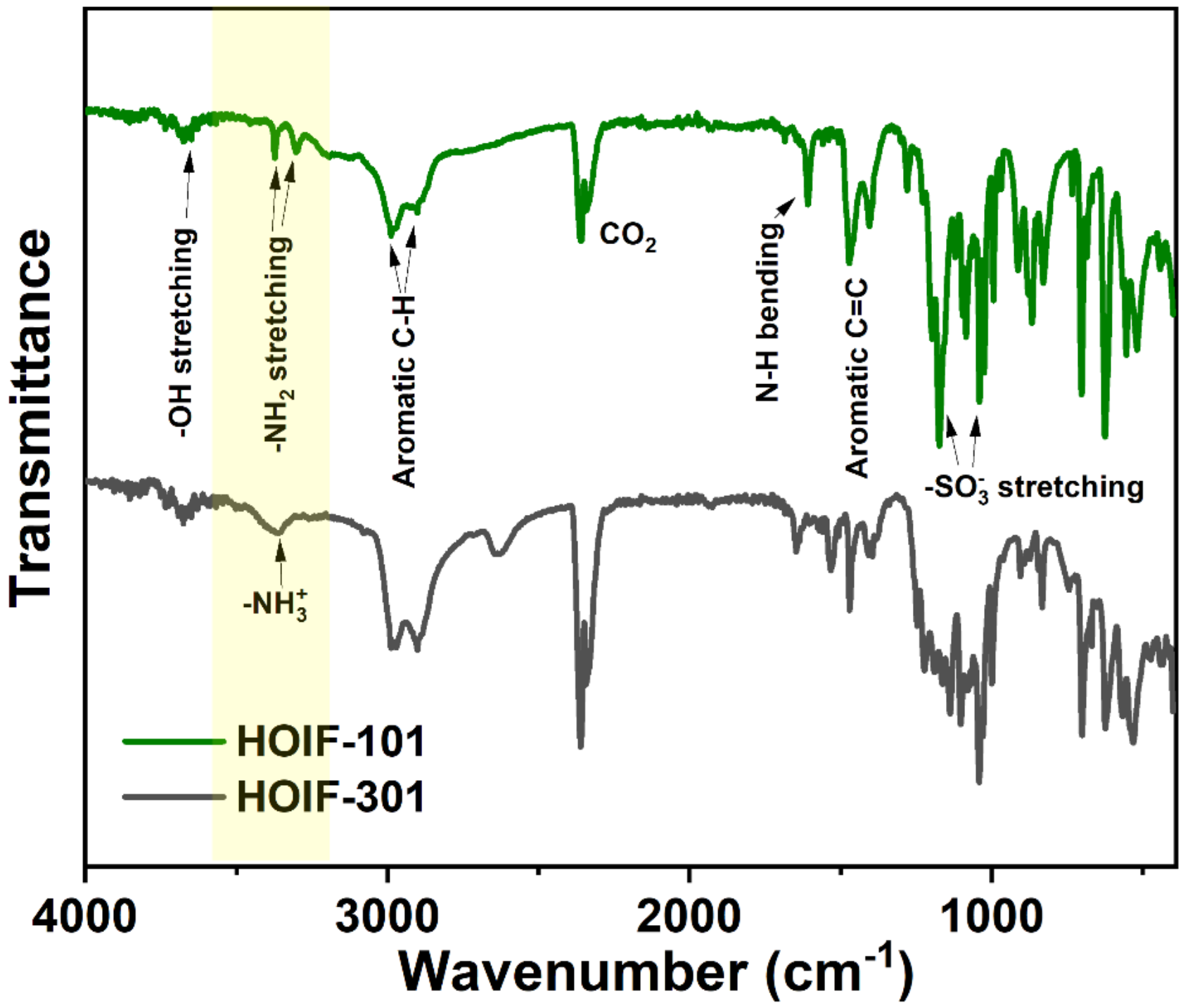

Figure S38. FTIR spectra of HOIF-101 and HOIF-301 in the range of 4000-400 cm^{-1}.

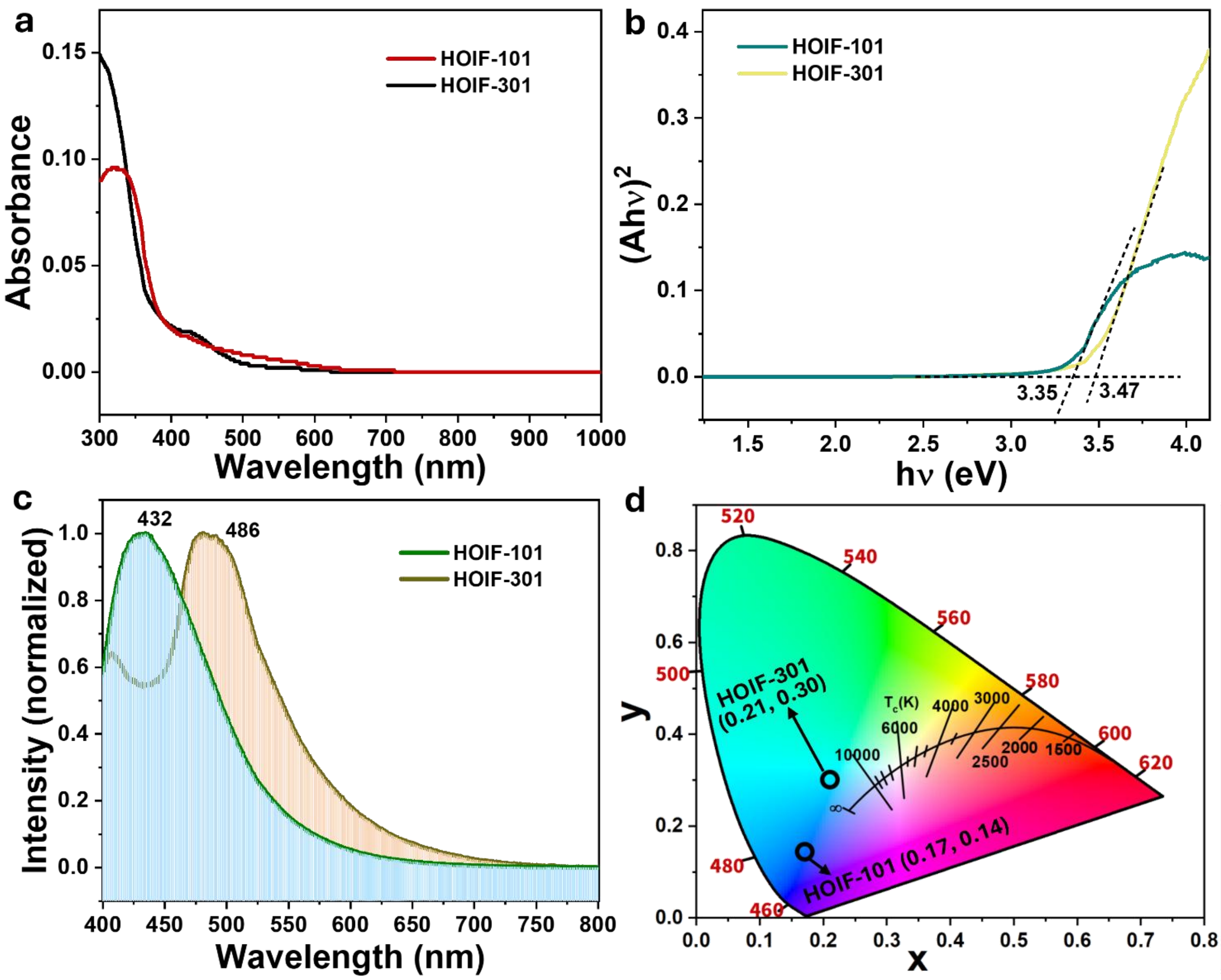

Figure S39. (a) UV–vis diffuse reflectance spectra and (b) corresponding Tauc plots of HOIF-101 and HOIF-301. The optical band gaps were estimated by substituting absorbance (Abs) for the molar absorption coefficient (α) in the equation of $(\alpha h\nu)^2 = A(h\nu\text{-}E_g)$.[1,23] (c) Fluorescent spectra and (d) corresponding Commission Internationale de l'Éclairage (CIE) 1931 chromaticity coordinates for HOIF-101 and HOIF-301 (excitation: 365 nm).

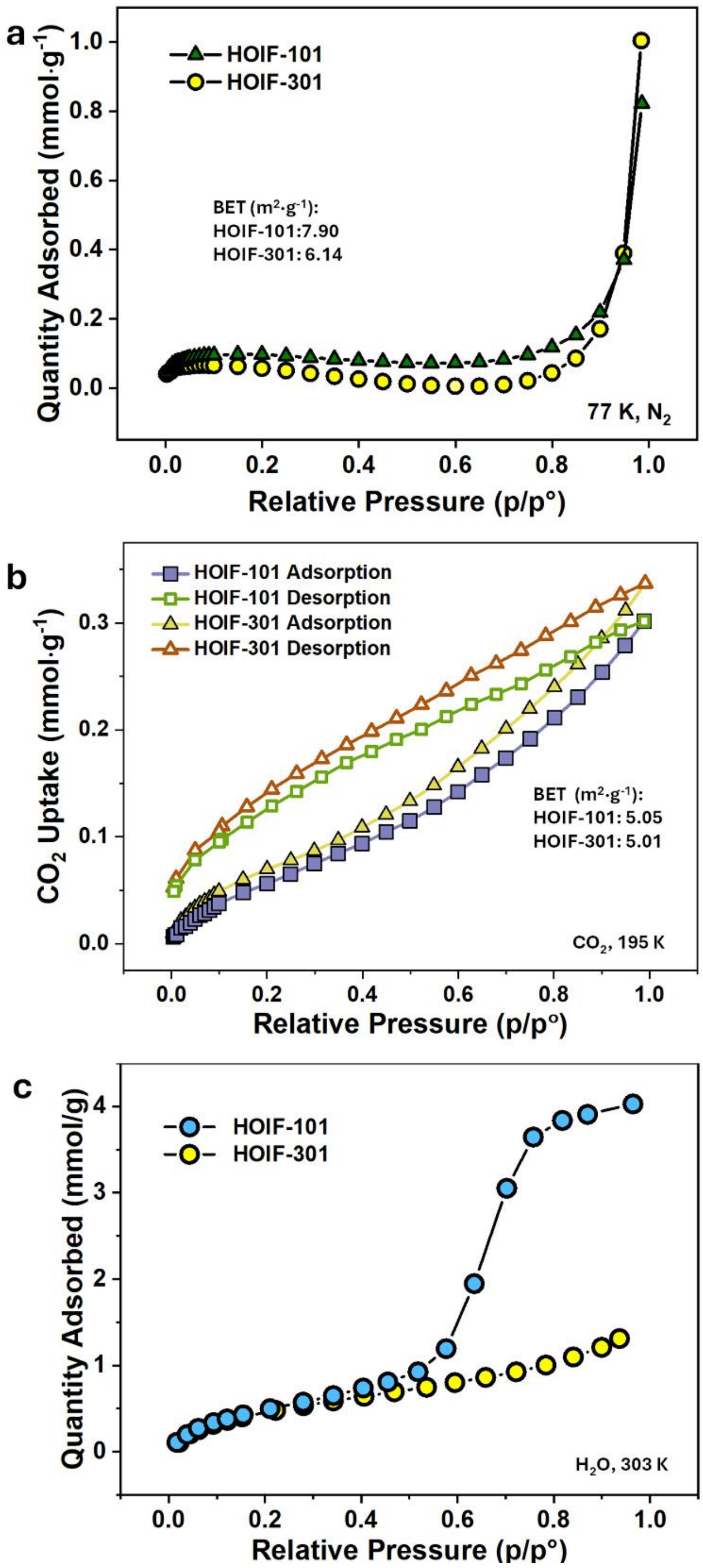

Figure S40. (a) Nitrogen gas sorption isotherms of HOIF-101 and HOIF-303 at 77 K. (b) CO_2 adsorption/desorption isotherms of HOIF-101 and HOIF-301 at 195 K. (c) Water vapor adsorption isotherms of HOIF-101 and HOIF-301 at 303 K.

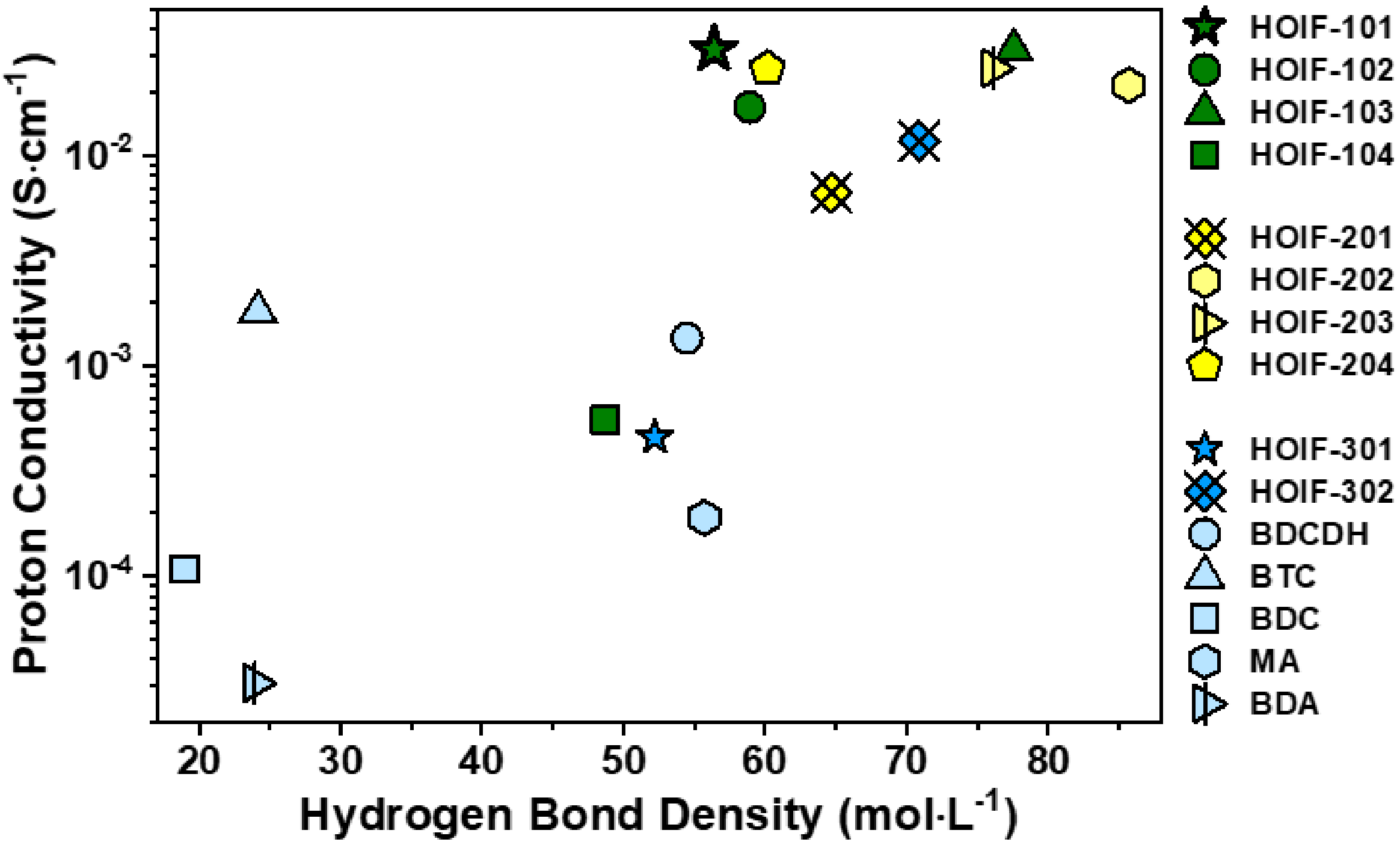

Figure S41. Illustration of the relationship between proton conductivity and hydrogen bond density in HOIFs and corresponding HOF counterparts.

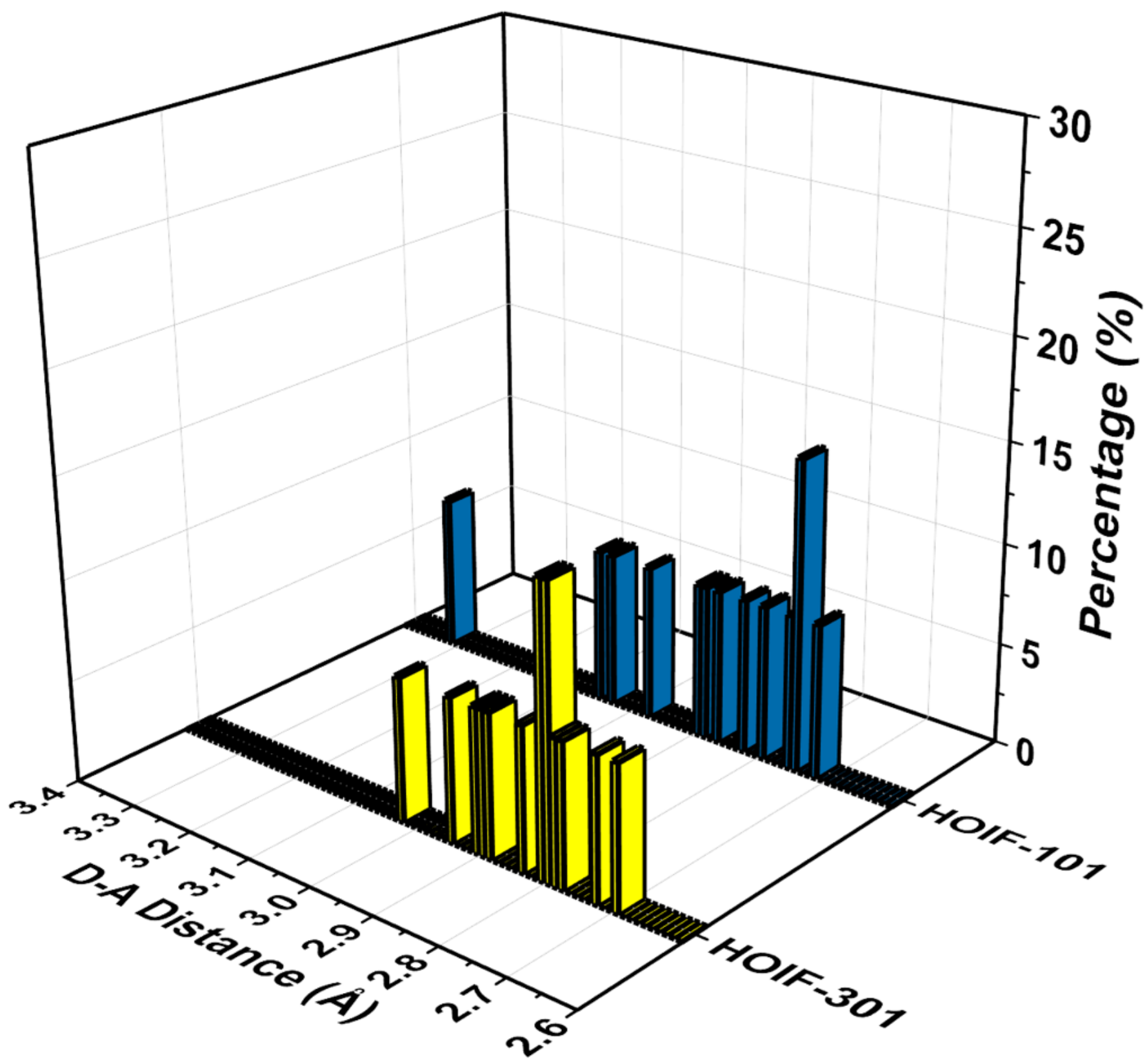

Figure S42. Distribution of hydrogen bond length (donor, D, to acceptor, A) of HOIF-101 and HOIF-301.

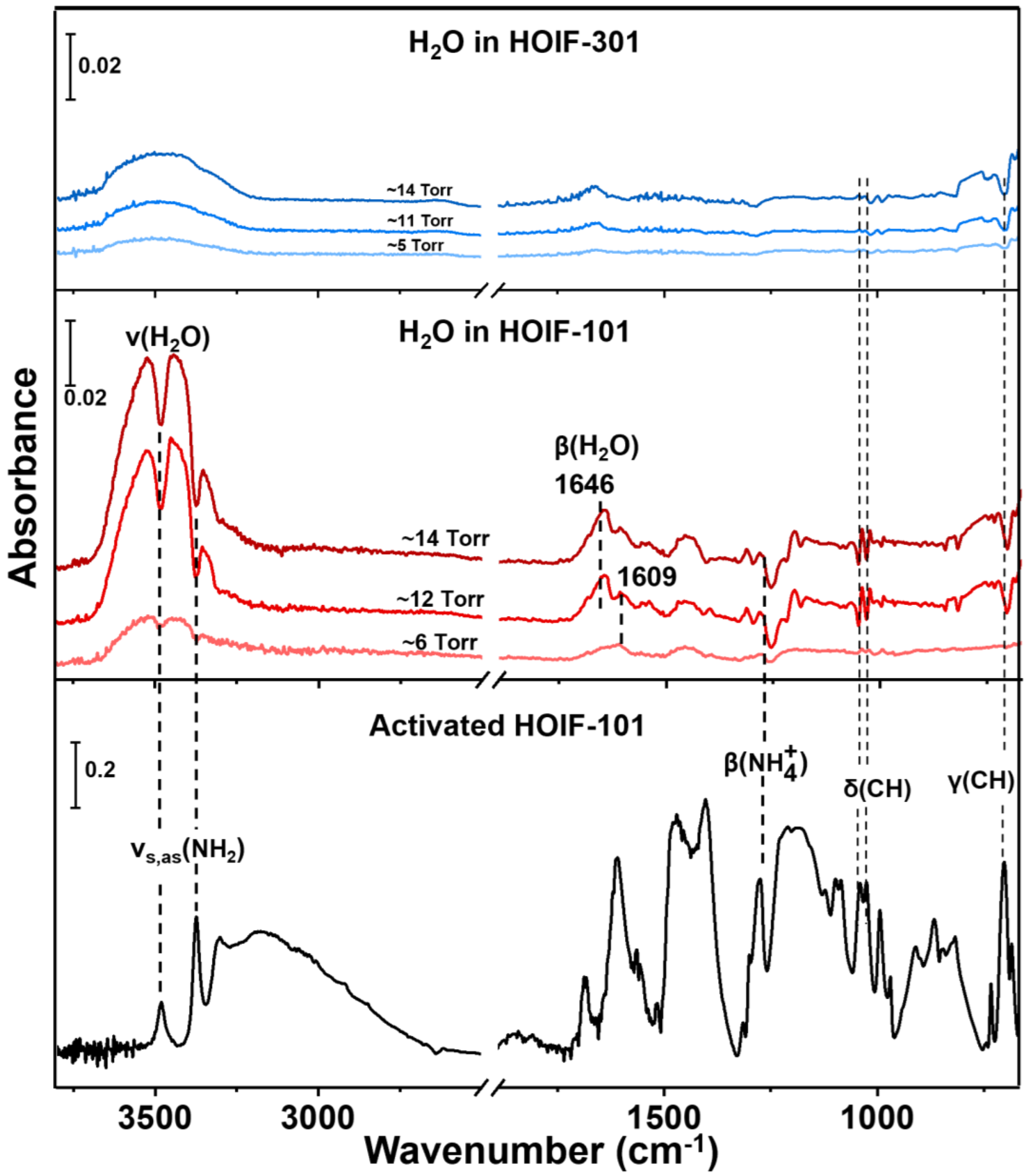

Figure S43. In situ IR difference spectra for HOIF-301 (top) and HOIF-101 (middle) upon increasing moisture pressure at 24 °C, referenced to the spectra of activated structures of HOIF-101 (bottom). Notation and acronym: *v* = stretching, *β* = bending (scissoring), *δ* = in plane deformation, *γ* = out of plane deformation, *s* = symmetric, *as* = asymmetric.

To probe the interaction of adsorbed water with HOIF structures, we conducted *in-situ* infrared (IR) spectroscopy measurements on HOIF-101 and HOIF-301 samples upon exposure to water moisture. The spectroscopic results are presented in (Figure S43). The amino/ammonia species in HOIF-101 structure are clearly characterized by the stretching bands (v_s/v_{as}) of amino groups at 3481/3374 cm^{-1} and bending band (β) of ammonia ions at 1275 cm^{-1}.[24] Upon water moisture exposure, the adsorbed H_2O is typified by its stretching (v) band at 3600-3000 cm^{-1} and bending (β) band at 1700-1600 cm^{-1} in the difference spectra. Close inspection of $\beta(H_2O)$ band in HOIF-101 reveals that it contains two components at 1609 and 1646 cm^{-1}. Both are upward (blue-) shifted with respect to free water molecule at 1595 cm^{-1}.[25] The 1646 cm^{-1} band becomes more prominent at higher vapor pressure. The large blue-shift (+51 cm^{-1}) indicates formation of hydrogen-bond network,[26] consistent with what is observed in isotherm measurement, *i.e.*, steep increase of water uptake above 0.4, p/p°. Along with the growth of water bands, significant perturbations occur to the bands belonging to ammonia species, *e.g.*, intensity

loss in the difference spectra. Such phenomenon has been observed in previous work and ascribed to the interaction of NH_3 with nearby water molecules through H-bonding that strongly freezes N-H bond oscillation.[24] Due to the perturbed v_s/v_{as} (NH_2) bands occurring in the same region, exact determination of water stretching band position is hindered in HOIF-101. In addition, phenyl ring CH deformation bands (δ/γ) in HOIF-101, which were shown sensitive toward guest molecule occupation in its proximity,[27,28] are noticeably perturbed, *e.g.*, derivative-like feature in the difference spectra. This implies their interaction of adsorbed water. Compared with HOIF-101, only phenyl δ/γ(CH) modes are slightly affected in HOIF-301. This provides direct evidence that addition of ammonia species helps to attract water within HOIF-101, promoting the formation of hydrogen-bonding networks and proton transport across the structure.

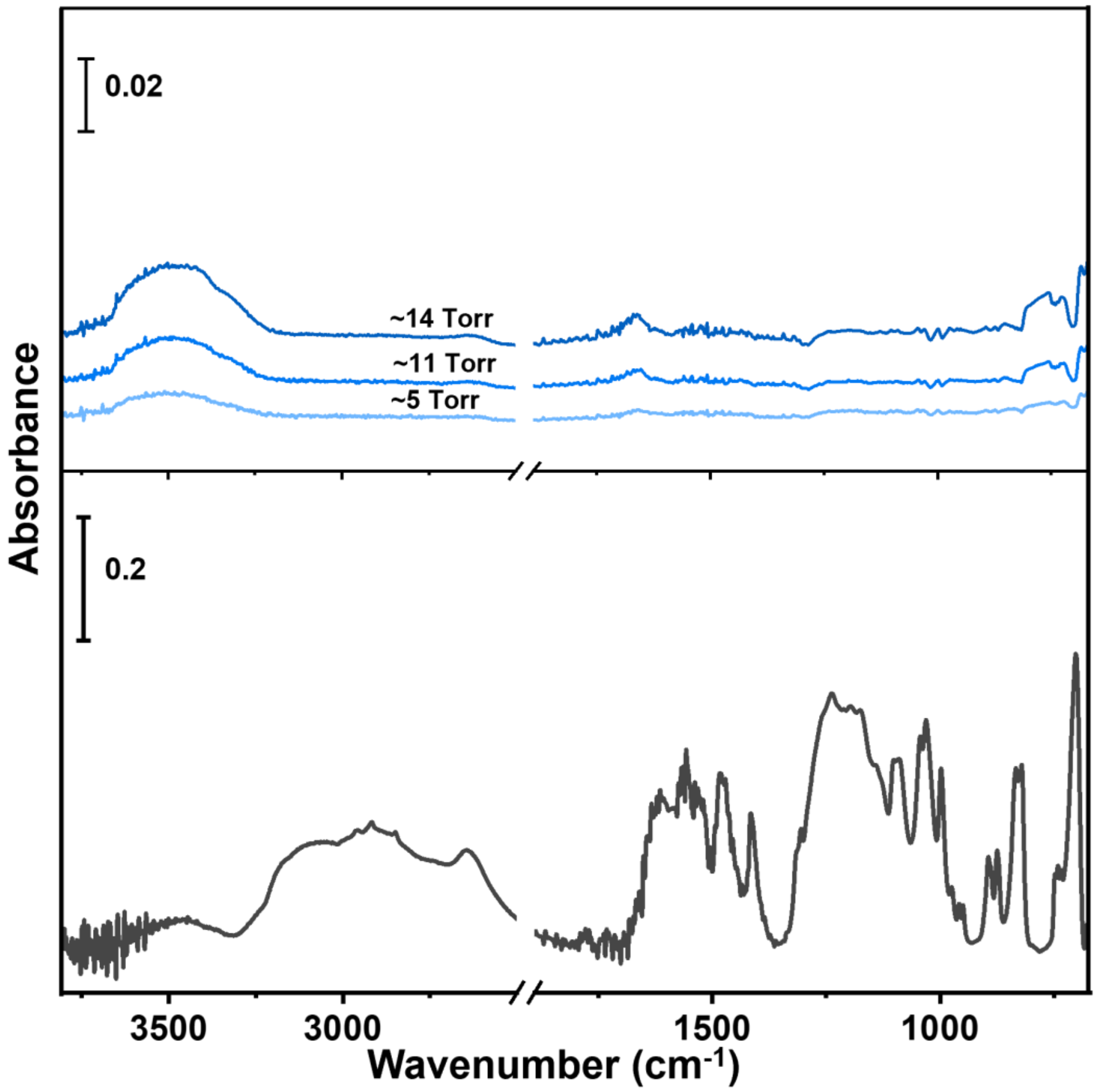

Figure S44. In situ IR difference spectra (top) for HOIF-301 upon increasing moisture pressure at 24 °C, referenced to the spectrum of activated structure of HOIF-301 (bottom).

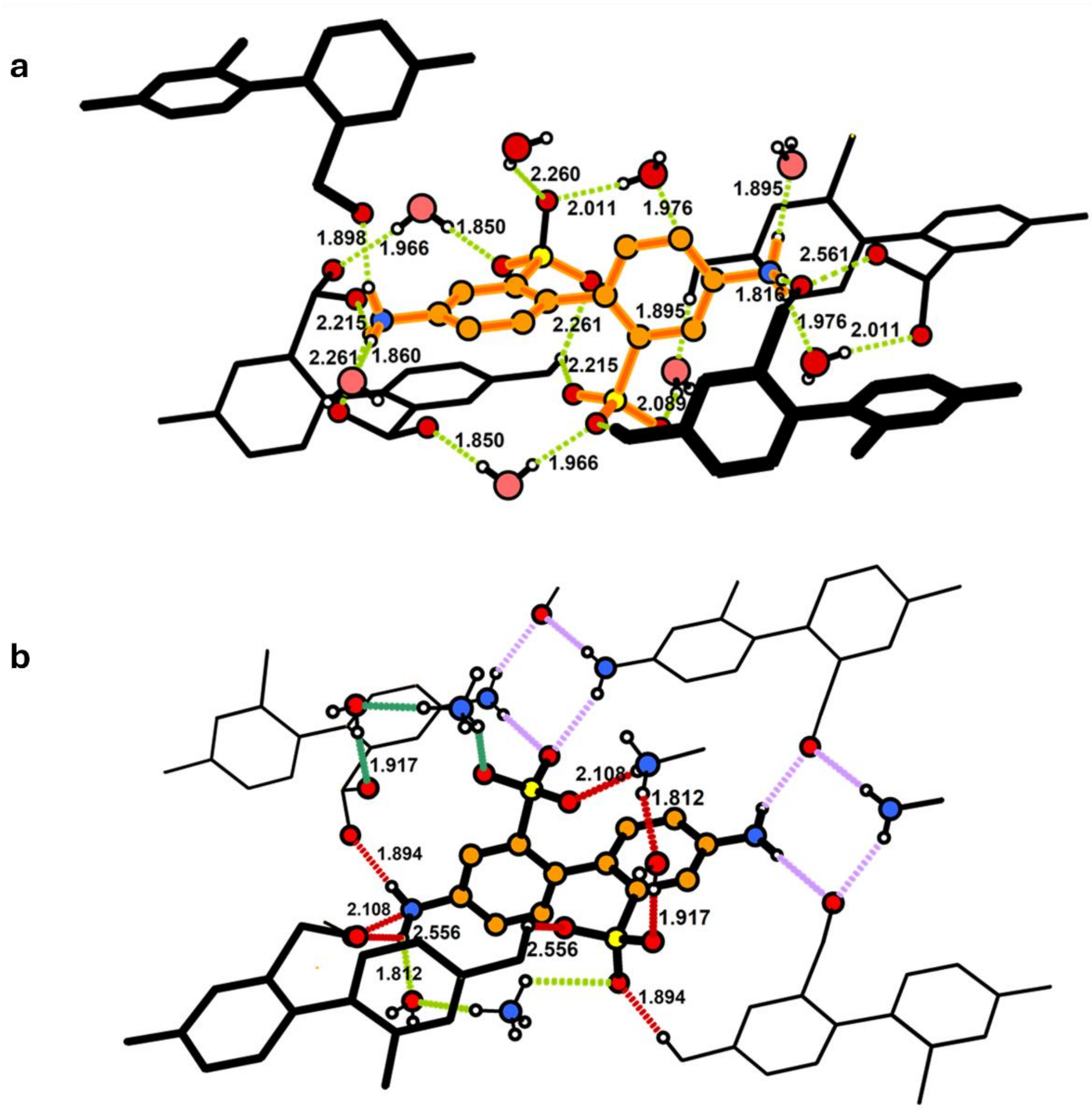

Figure S45. Illustration and comparison of hydrogen bond length of (a) HOIF-301 and (b) HOIF-101 in terms of the hydrogen-bonding interactions in $-NH_3^+\cdots OH_2$, $-NH_3^+\cdots O_3^-S-$ and $-SO_3^-\cdots H_2O$.

In HOIF-101, the mobile protons originate from partial proton transfer from the sulfonic acid groups to NH_3/-NH_2, forming NH_4^+/-NH_3^+, which can subsequently donate a proton to a neighboring water molecule or sulfonate, initiating Grötthuss-type hopping. The relatively weak acidity of these species counterintuitively with high proton conductivity can be explained as follows: DADS molecules in HOIF-301 are zwitterions: all sulfonic acid protons are fully transferred to the amino groups, and no NH_4^+ is present. When DADS reacts with $NH_3{\cdot}H_2O$ to form HOIF-101, a competitive protonation occurs between the amino groups of DADS and the incoming NH_3 molecules, beneficial to improve proton mobility. In addition, incorporation of NH_4^+ significantly shortens the hydrogen-bond distances between ammonium ions and sulfonate groups, water molecules (Table S11, Figure S45). This is evidenced by SC-XRD, XPS (higher binding energies of N 1s and O 1s), DFT interaction energies, and IGMH analysis. These shorter, stronger, and more directional hydrogen bonds make the proton motions around NH_4^+ more intense, i.e., the protons are more readily "pulled" by neighboring acceptors (-SO_3^-, H_2O). Under an external electric field, this intensified local dynamics translates into efficient long-range proton migration in the dense, uninterrupted H-bond networks. Thus, even though NH_4^+ and -NH_3^+ are relatively weak Brønsted acids, the continuous and dense hydrogen-bond network compensates for the moderate proton-donor strength or low density of proton sources by dramatically improving proton mobility. This explains how HOIF-101 achieves superprotonic conductivity without strong acid doping. This also demonstrates the effectiveness of such a metal-free hybridization to enhance proton conductivity, which is conceptually complementary to traditional strong-acid functionalization and expands the design space for high-performance solid proton conductors.

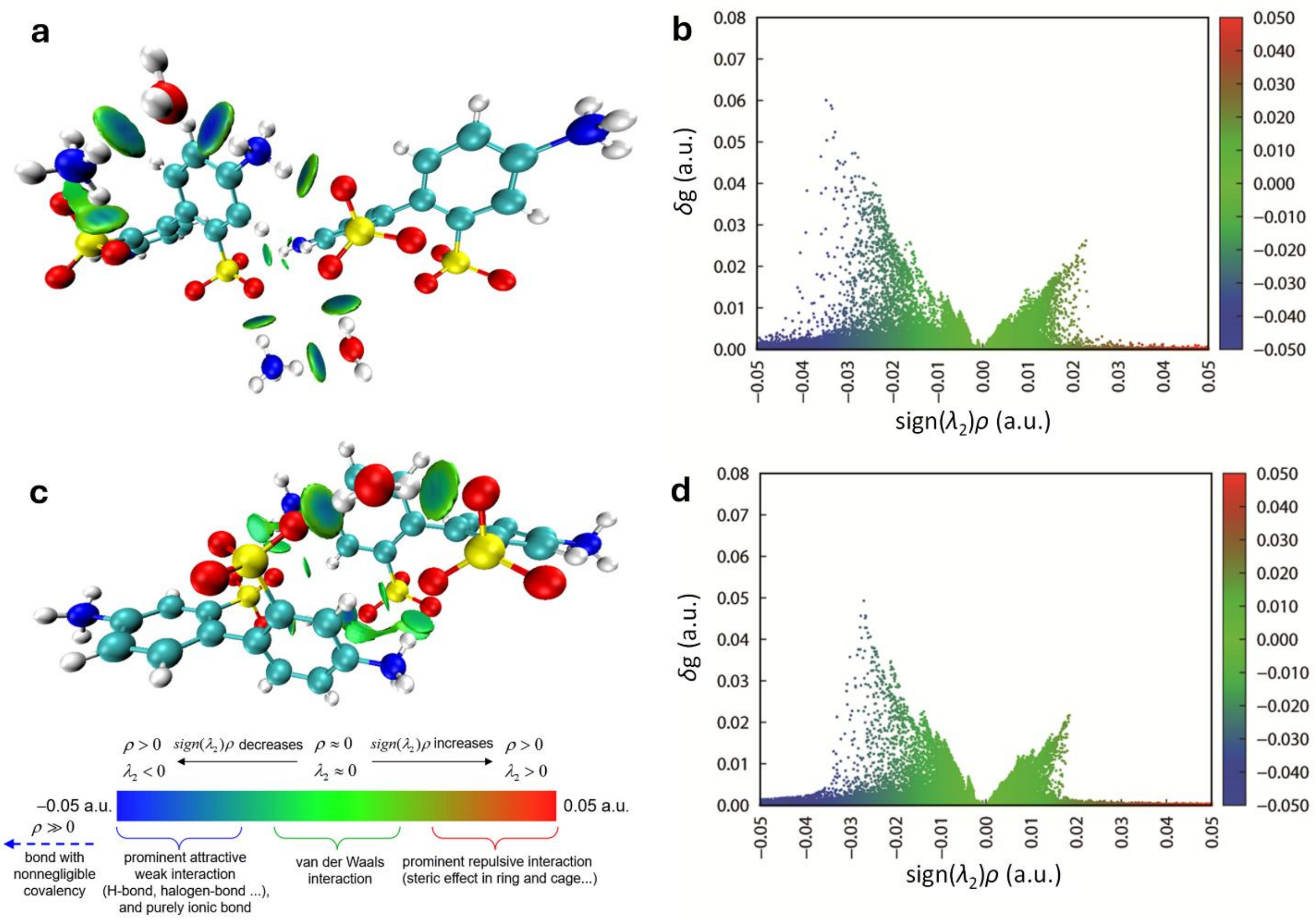

Figure S46. (a) IGMH isosurfaces for HOIF-101, illustrating intermolecular interaction regions based on the independent gradient model with Hirshfeld partition. The color scale represents the value of sign(λ_2)ρ, where blue, green, and red correspond to attractive (hydrogen bonding), van der Waals, and repulsive (steric repulsion) interactions, respectively. (b) Corresponding scatter plots of the associated gradient (δg) against sign(λ_2)ρ for HOIF-101, providing quantitative visualization of the interaction strength and type. (c) IGMH isosurfaces and (d) corresponding scatter plots for HOIF-301.

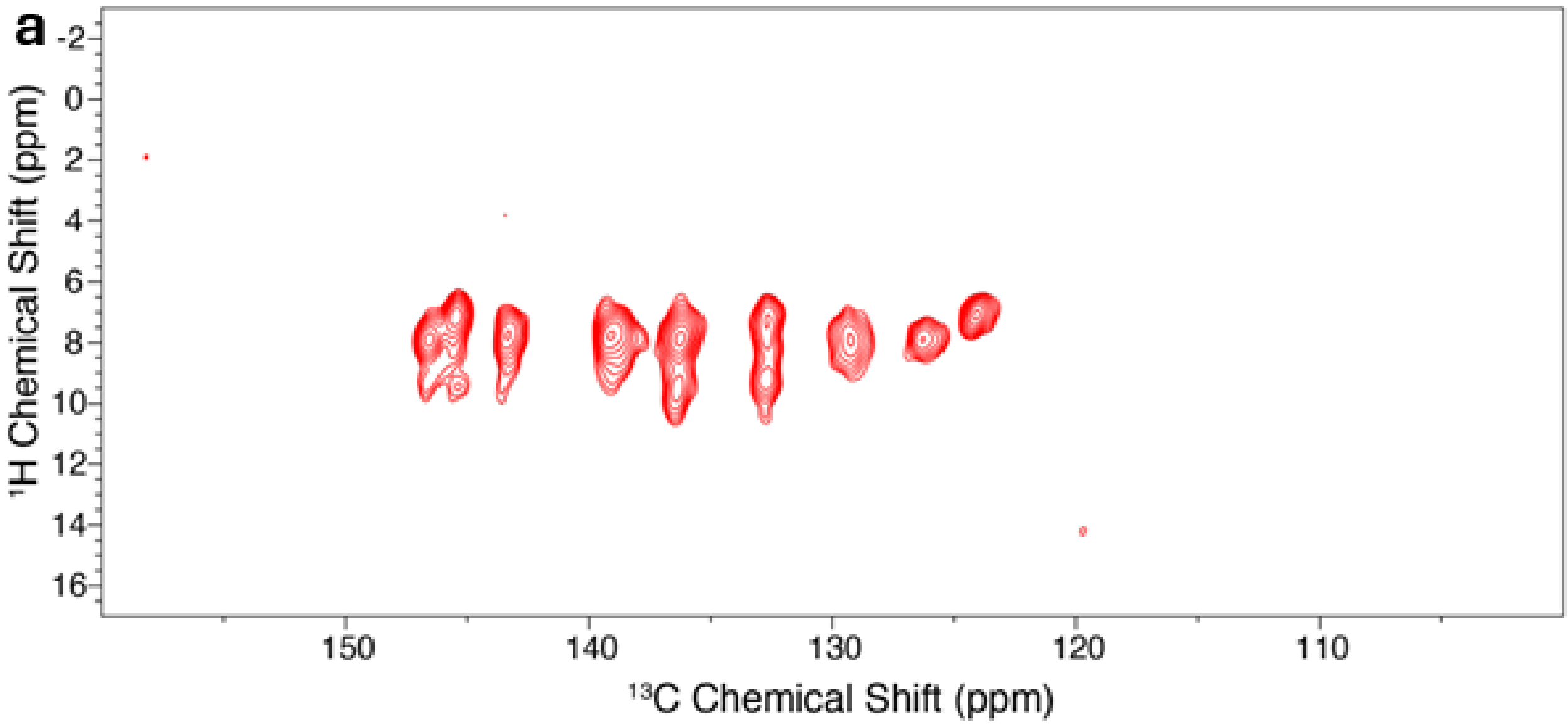

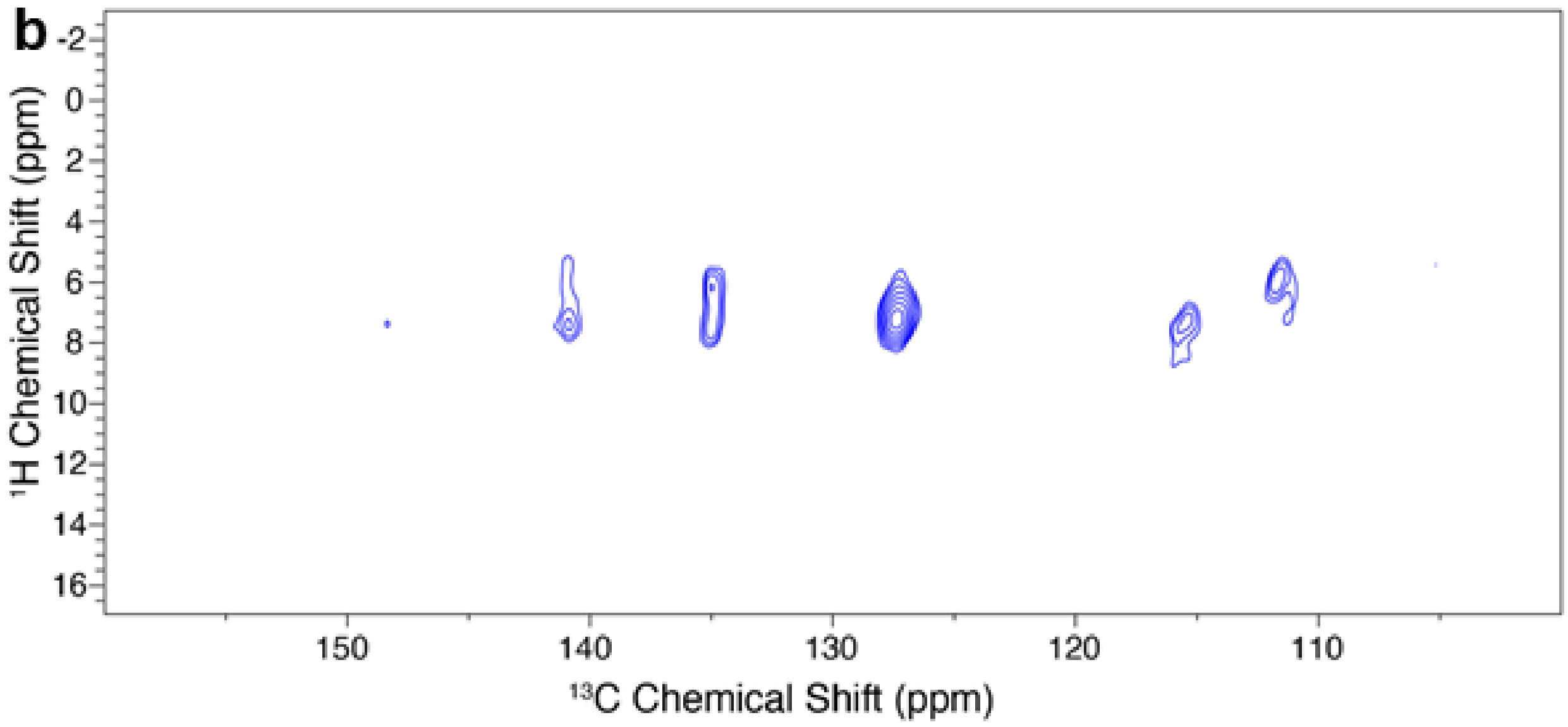

Figure S47. Cross-polarization-based ^{1}H-^{13}C heteronuclear correlation 2D MAS NMR spectra of (a) HOIF-301 (red) and (b) HOIF-101 (blue). The spectra confirm the peaks between 6 and 10 ppm in the 1H spectra belong to aromatic protons attached to the phenyl rings in the organic portion of both HOIFs.

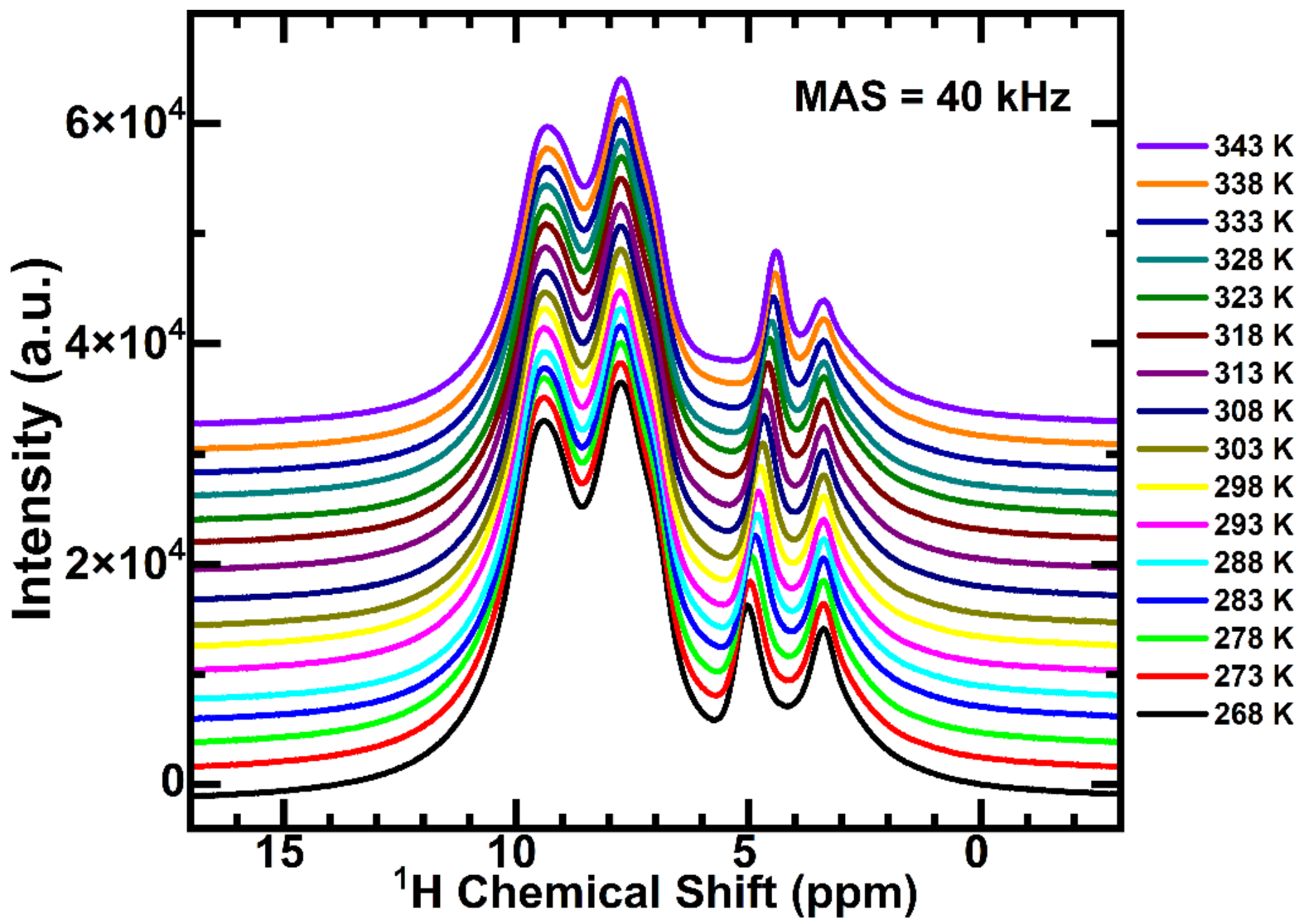

Figure S48. MAS ^{1}H NMR spectra of HOIF-301 at different temperatures (spinning at 40 kHz).

The ^{1}H 1D MAS NMR spectrum of HOIF-301 (50 °C VT set point, 40 kHz MAS) displays two well-resolved resonances at ~3.2 and 4.5 ppm. T_2 analysis (T_2 = 1.0 and 1.5 ms) indicates that these signals arise from relatively mobile proton species, in contrast to the broad feature spanning 6–11 ppm, which exhibits a much shorter T_2 (0.25 ms). Two-dimensional ^{1}H–^{13}C heteronuclear correlation (HC) experiments (Figure S47) assign this broad resonance to aromatic protons in the organic component. In HOIF-101, the broad aromatic signal persists with a comparable T_2 value (0.27 ms), while only a single mobile resonance at ~5.0 ppm remains, exhibiting a markedly longer T_2 (1.6 ms at 0 °C). Notably, this resonance shows pronounced temperature-dependent chemical shifts and progressive line broadening upon heating, indicative of entry into an intermediate chemical exchange regime. By contrast, the mobile resonances in the pristine HOIF-301 remain sharp even at elevated temperatures, suggesting the absence of chemical exchange. These observations imply that proton motion in HOIF-301 is largely restricted to local dynamics, whereas in HOIF-101 the presence of ammonia species enables dynamic chemical exchange associated with larger-amplitude proton motions.

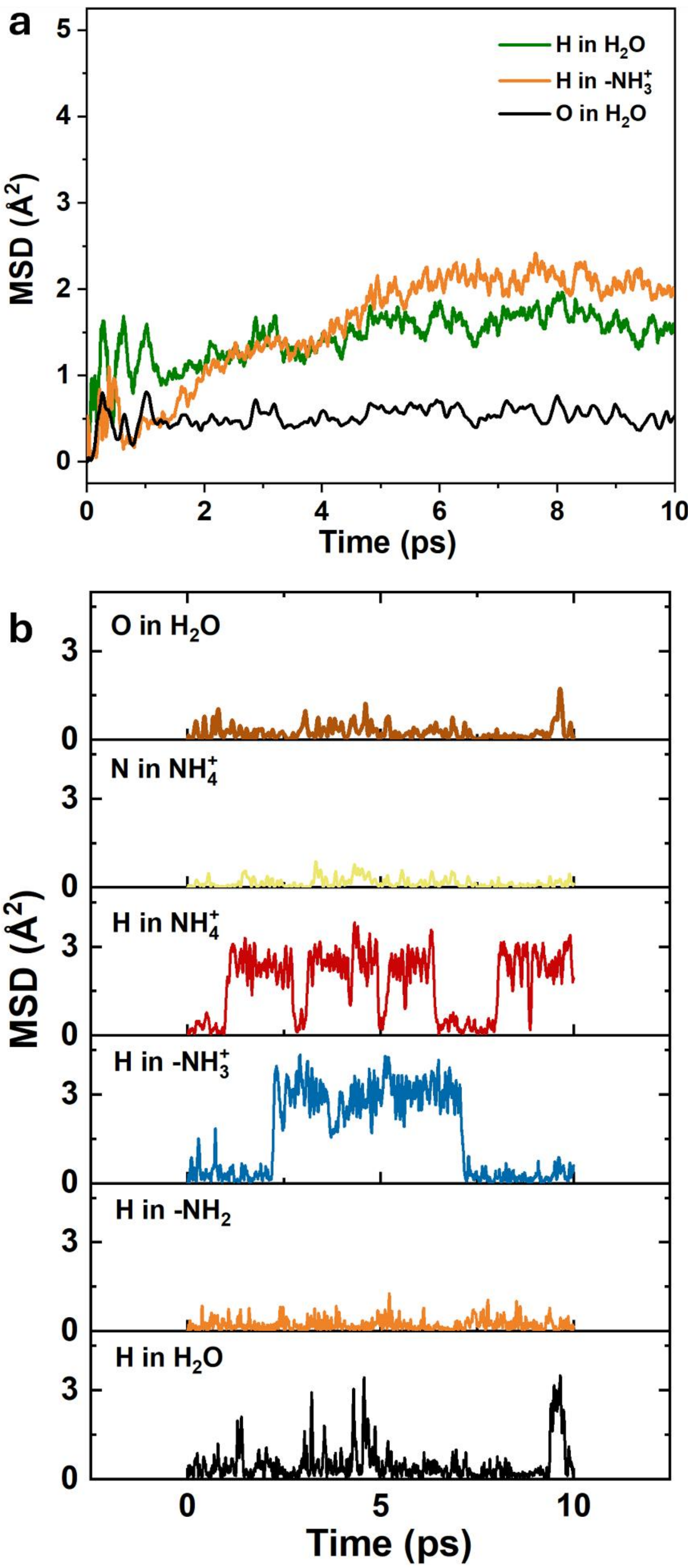

Figure S49. Mean square displacement (MSD) as a function of time derived from MD simulations for (a) HOIF-301 and (b) HOIF-101.

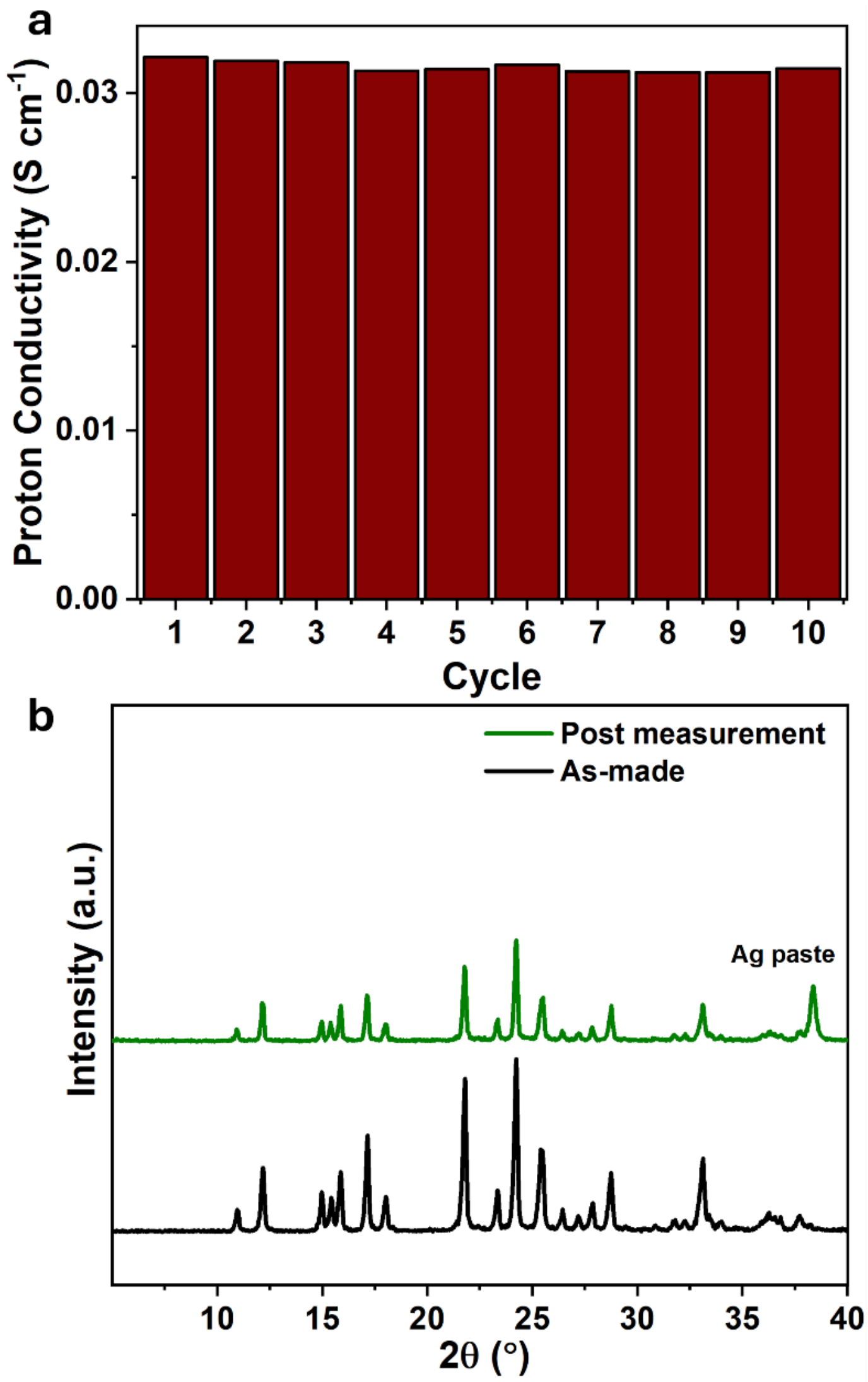

Figure S50. (a) Proton conductivity of HOIF-101 measured during heating-cooling (drying) cycling. (b) PXRD patterns of as-synthesized and recycled HOIF-101.

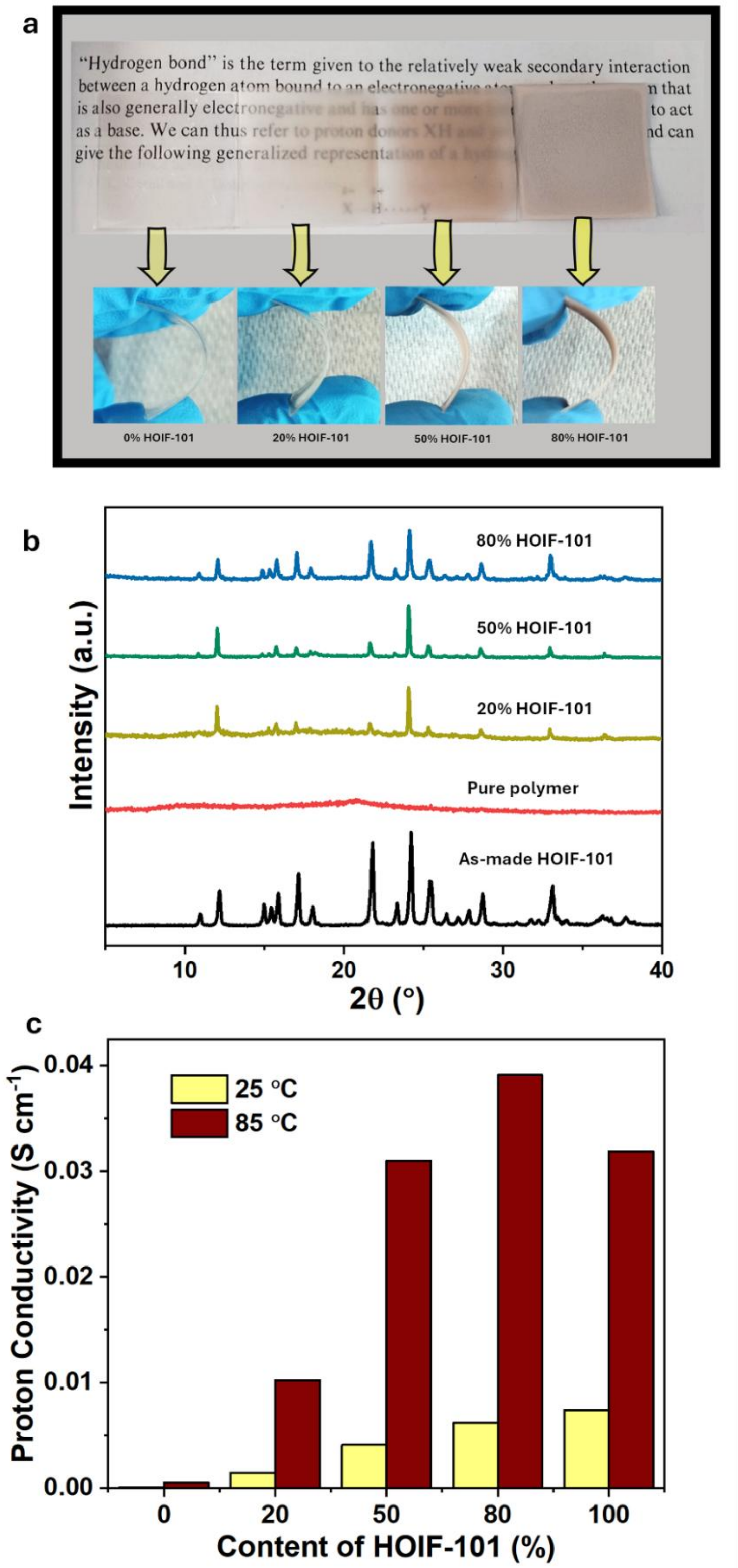

Figure S51. (a) Photographs of mixed matrix membranes of HOIF-101 with different loading amounts. (b) PXRD patterns and (c) proton conductivity of as synthesized HOIF-101, PVP/PVDF polymer and HOIF-101 membranes with different loading amount of HOIF-101.

3. Supplementary Tables

Table S1. Single crystal data and structural refinement for HOIF-101 ~ HOIF-104.

	HOIF-101	HOIF-102	HOIF-103	HOIF-104
Empirical formula	$C_{12}H_{17}N_3O_7S_2$	$C_8H_{12}N_2O_6$	$C_9H_{19}N_3O_8$	$C_8H_{12}N_2O_4$
Formula weight	379.4	232.2	297.27	200.2
Temperature (K)	100(2)	100(2)	100(2)	298.15
Crystal system	triclinic	monoclinic	monoclinic	monoclinic
Space group	$P\bar{1}$	$P2_1/n$	$P2_1/n$	$P2_1/c$
a (Å)	8.4254(9)	3.7779(4)	7.9355(10)	20.883(2)
b (Å)	8.7305(9)	8.8107(8)	18.745(2)	3.9934(4)
c (Å)	12.0735(13)	14.4628(14)	18.636(2)	11.7357(11)
α (°)	79.889(4)	90	90	90
β (°)	80.597(4)	95.429(3)	101.248(5)	106.068(4)
γ (°)	61.223(4)	90	90	90
Volume (Å^3)	763.12(14)	479.25(8)	2718.9(5)	940.46(16)
Z	2	2	8	4
ρ_{calc} (g·cm^{-3})	1.651	1.609	1.452	1.414
μ (mm^{-1})	0.415	0.146	0.134	0.12
F(000)	396	244	1264	424
Crystal size (mm^3)	0.17 × 0.1 × 0.01	0.29 × 0.02 × 0.01	0.33 × 0.03 × 0.02	0.15 × 0.05 × 0.02
Radiation (Å)	synchrotron (λ = 0.7288)	synchrotron (λ = 0.7288)	synchrotron (λ = 0.7288)	synchrotron (λ = 0.7288)
2θ range for data collection (°)	3.528 ~ 62.854	5.558 ~ 62.836	3.192 ~ 58.282	3.704 ~ 54.374
Index ranges	$-12 \le h \le 12$, $-12 \le k \le 12$, $-17 \le l \le 17$	$-5 \le h \le 5$, $-12 \le k \le 12$, $-20 \le l \le 20$	$-10 \le h \le 10$, $-25 \le k \le 25$, $-24 \le l \le 24$	$-26 \le h \le 26$, $-4 \le k \le 4$, $-14 \le l \le 14$
Reflections collected	24542	15938	85480	12803
Independent reflections	4679 [R_{int} = 0.0562, R_{sigma} = 0.0414]	1473 [R_{int} = 0.0456, R_{sigma} = 0.0240]	6763 [R_{int} = 0.0651, R_{sigma} = 0.0309]	1891 [R_{int} = 0.0661, R_{sigma} = 0.0510]
Data/restraints/parameters	4679/650/349	1473/0/97	6763/0/489	1891/174/188
Goodness-of-fit on F^2	1.064	1.102	1.04	1.071
Final R indexes [I>=2σ(I)]	R_1 = 0.0517, wR_2 = 0.1355	R_1 = 0.0313, wR_2 = 0.0883	R_1 = 0.0357, wR_2 = 0.0963	R_1 = 0.0488, wR_2 = 0.1313
Final R indexes [all data]	R_1 = 0.0650, wR_2 = 0.1432	R_1 = 0.0340, wR_2 = 0.0905	R_1 = 0.0500, wR_2 = 0.1065	R_1 = 0.0584, wR_2 = 0.1435
Largest diff. peak/hole (eÅ^{-3})	0.99/-0.68	0.56/-0.24	0.42/-0.28	0.42/-0.34
CCDC number	2490175	2490177	2490178	2490179

Table S2. Single crystal data and structural refinement for HOIF-201 ~ HOIF-204.

	HOIF-201	HOIF-202	HOIF-203	HOIF-204
Empirical formula	$C_{24}H_{36}N_8O_{17}S_5$	$C_3H_8N_6O_4S$	$C_6H_{12}N_2O_8S_2$	$C_{12}H_{24}N_4O_{13}S_3$
Formula weight	868.91	224.21	304.3	528.53
Temperature (K)	100(2)	100(2)	100(2)	100(2)
Crystal system	monoclinic	monoclinic	monoclinic	monoclinic
Space group	$I\,2/a$	$C\,2/c$	$P\,2_1/n$	$P\,2_1/c$
a (Å)	6.1484(5)	18.4586(18)	4.6243(4)	17.4170(18)
b (Å)	16.6902(14)	8.6538(8)	5.6128(5)	17.7074(18)
c (Å)	16.0197(16)	12.724(2)	21.854(2)	13.3511(14)
α (°)	90	90	90	90
β (°)	90.033(3)	130.290(2)	90.254(4)	93.043(4)
γ (°)	90	90	90	90
Volume (Å^3)	1643.9(3)	1550.3(4)	567.23(9)	4111.8(7)
Z	2	8	2	8
ρ_{calc} (g·cm^{-3})	1.755	1.921	1.782	1.708
μ (mm^{-1})	0.472	0.447	0.537	0.463
F(000)	904	928	316	2208
Crystal size (mm^3)	0.33 × 0.01 × 0.01	0.05 × 0.05 × 0.05	0.07 × 0.05 × 0.01	0.08 × 0.06 × 0.05
Radiation	synchrotron (λ = 0.7288)	synchrotron (λ = 0.7288)	synchrotron (λ = 0.7288)	synchrotron (λ = 0.7288)
2θ range for data collection (°)	3.614 ~ 62.794	5.666 ~ 68.234	3.822 ~ 60.09	3.366 ~ 62.798
Index ranges	$-8 \le h \le 8$, $-23 \le k \le 23$, $-22 \le l \le 22$	$-28 \le h \le 28$, $-13 \le k \le 10$, $-19 \le l \le 19$	$-6 \le h \le 6$, $-7 \le k \le 7$, $-30 \le l \le 29$	$-24 \le h \le 24$, $-25 \le k \le 25$, $-18 \le l \le 18$
Reflections collected	23387	12389	12466	139383
Independent reflections	2525 [R_{int} = 0.0606, R_{sigma} = 0.0325]	2953 [R_{int} = 0.0470, R_{sigma} = 0.0409]	1529 [R_{int} = 0.0756, R_{sigma} = 0.0430]	12498 [R_{int} = 0.0687, R_{sigma} = 0.0349]
Data/restraints/parameters	2525/0/158	2953/0/159	1529/0/106	12498/9/766
Goodness-of-fit on F^2	1.294	1.038	1.128	1.03
Final R indexes [I>=2σ(I)]	R_1 = 0.0626, wR_2 = 0.1490	R_1 = 0.0319, wR_2 = 0.0877	R_1 = 0.0461, wR_2 = 0.1151	R_1 = 0.0374, wR_2 = 0.0954
Final R indexes [all data]	R_1 = 0.0644, wR_2 = 0.1499	R_1 = 0.0362, wR_2 = 0.0917	R_1 = 0.0511, wR_2 = 0.1217	R_1 = 0.0489, wR_2 = 0.1024
Largest diff. peak/hole (eÅ^{-3})	0.44/-0.56	0.59/-0.52	0.46/-0.56	0.83/-0.72
CCDC number	2490180	2490181	2490182	2490183

Table S3. Single crystal data and structural refinement for HOIF-301 and HOIF-302.

	HOIF-301	HOIF-302
Empirical formula	$C_{12}H_{18}N_2O_9S_2$	$C_{12}H_{22}N_4O_9S_2$
Formula weight	398.4	430.45
Temperature (K)	100(2)	100(2)
Crystal system	triclinic	triclinic
Space group	$P\bar{1}$	$P\bar{1}$
a (Å)	8.3243(8)	9.0760(7)
b (Å)	9.4961(9)	10.6552(8)
c (Å)	11.1038(10)	11.0777(8)
α (°)	93.936(3)	114.327(2)
β (°)	109.099(3)	94.158(3)
γ (°)	90.836(3)	109.742(2)
Volume (Å^3)	826.83(13)	890.37(12)
Z	2	2
ρ_{calc} (g·cm^{-3})	1.600	1.606
μ (mm^{-1})	0.395	0.376
F(000)	416	452
Crystal size (mm^3)	0.1 × 0.07 × 0.05	0.1 × 0.04 × 0.01
Radiation	synchrotron (λ = 0.7288)	synchrotron (λ = 0.7288)
2θ range for data collection (°)	3.992 ~ 68.278	4.27 ~ 68.298
Index ranges	-12 ≤ h ≤ 12, -14 ≤ k ≤ 14, -17 ≤ l ≤ 17	-13 ≤ h ≤ 13, -16 ≤ k ≤ 16, -17 ≤ l ≤ 16
Reflections collected	29133	32542
Independent reflections	6278 [R_{int} = 0.0367, R_{sigma} = 0.0317]	6772 [R_{int} = 0.0371, R_{sigma} = 0.0309]
Data/restraints/parameters	6278/0/298	6772/0/332
Goodness-of-fit on F^2	1.053	1.049
Final R indexes [I>=2σ(I)]	R_1 = 0.0322, wR_2 = 0.0837	R_1 = 0.0336, wR_2 = 0.0900
Final R indexes [all data]	R_1 = 0.0382, wR_2 = 0.0870	R_1 = 0.0433, wR_2 = 0.0955
Largest diff. peak/hole (eÅ^{-3})	0.54/-0.4	0.56/-0.53
CCDC number	2490184	2490185

Table S4. Summary of topology analysis for HOIFs and related compounds.

	Name	Configuration	Point Symbol
HOIF-101	***hoi******	5,10-c net	$\{3^3.4^7\}\{3^6.4^{18}.5^{18}.6^3\}$
HOIF-102	***coa******	4,8-c net	$\{4^{10}.6^{14}.8^4\}\{4^5.6\}_2$
HOIF-103	***cta******	5,5,5,6,10,11-c net	$\{4^{10}\}_3\{4^{13}.6^2\}\{4^{28}.6^{17}\}\{4^{32}.6^{23}\}$
HOIF-104	***cca******	5,10-c net	$\{4^{26}.6^{18}.8\}\{4^9.6\}_2$
HOIF-201	***hio******	4,8-c net	$\{3^6.4^{12}.5^8.6^2\}_2\{4.5^5\}$
HOIF-202	***mes******	6,7-c net	$\{3.4^{10}.5^3.6\}\{3^2.4^{11}.5^5.6^3\}$
HOIF-203	***pas******	6,8-c net	$\{3^4.4^{16}.5^8\}\{3^4.4^6.5^4.6\}_2$
HOIF-204	***mas******	4,5,5,5,9,12-c net	$\{3.4^7.5.6\}\{3.4^8.5\}\{3^2.4^5.5^3\}\{3^4.4^{17}.5^4.6^{11}\}\{3^4.4^{22}.5^6.6^{30}.7^2.8^2\}\{4^5.6\}$
HOIF-301	***oih******	3,5,12-c net	$\{3.4^2\}\{3^2.4^5.5^3\}\{3^6.4^{25}.5^{23}.6^{12}\}$
HOIF-302	***das******	6,6,8-c net	$\{3^2.4^{10}.5^2.6\}\{3^2.4^{14}.5^5.6^7\}\{3^2.4^9.5^2.6^2\}$
BDCDH	***bdc******	2-c net	{0}
BTC	***hcb***	3-c net	$\{6^3\}$
BDC	***bdc******	2-c net	{0}
MA	***dmp***	4-c net	$\{6^5.8\}$
BDA	***cds***	4-c net	$\{6^5.8\}$
mBDA	***fes***	3-c net	$\{4.8^2\}$

*Novel topological types

Table S5. Proton conductivity and activation energy of HOIFs and related organic compounds.

**HOIFs**	**σ (S·cm^{-1})***	**E_a (eV)**	**Dimension of H-bonding networks (D)**
HOIF-101	3.19×10^{-2}	0.25	3
HOIF-102	1.69×10^{-2}	0.42	3
HOIF-103	3.18×10^{-2}	0.33	3
HOIF-104	5.25×10^{-4}	0.93	2
HOIF-201	6.64×10^{-3}	0.22	2
HOIF-202	2.16×10^{-2}	0.25	3
HOIF-203	2.60×10^{-2}	0.15	2
HOIF-204	2.58×10^{-2}	0.23	3
HOIF-301 (DADS)	4.56×10^{-4}	0.39	3
HOIF-302 (DAS)	1.17×10^{-2}	0.18	2

**Organic building units**	**σ (S·cm^{-1})**	**E_a (eV)**
BDCDH	1.35×10^{-3}	1.27
BTC	1.80×10^{-3}	1.36
BDC	1.07×10^{-4}	2.61
MA	1.89×10^{-4}	0.71
BDA	3.09×10^{-5}	0.46

*85 °C and 100% RH. Note that σ for mBDA was unmeasurable at 85 °C.

Table S6. Comparison of proton conductivity of exemplified HOFs, their representative, descendant HOIFs and MOFs without extra modification.

**Name**	***T*** **(℃)**	***RH*** **(%)**	***σ*** **(S/cm)**	E_a **(eV)**	***Refs***
BDC	85	100	1.07×10^{-4}	2.61	This work
HOIF-104	85	100	5.25×10^{-4}	0.93	This work
Ce-BDC	100	98	1.63×10^{-5}	0.56	[29]
UiO-66	80	90	4.30×10^{-6}	0.43	[30,31]
MIL-53	80	95	3.60×10^{-7}	0.47	[32]
BTC	85	100	1.80×10^{-3}	1.36	This work
HOIF-103	85	100	3.18×10^{-2}	0.33	This work
MOF-808	80	98	4.21×10^{-5}	0.90	[33-36]
Ca-BTC	72	98	3.20×10^{-5}	0.18	[37]
HKUST-1	90	70	1.08×10^{-8}	0.69	[38,39]
BDCDH	85	100	1.35×10^{-3}	1.27	This work
HOIF-102	85	100	1.69×10^{-2}	0.42	This work
Ho-BDCDH	70	98	2.53×10^{-4}	0.41	[40]
MOF-74(Ni)	80	95	1.40×10^{-4}	0.20	[41]
CPO-27(Co)	90	95	2.50×10^{-7}	0.30	[42]

Table S7. Comparison of Proton conductivity of HOIF and HOF materials.

Name	*T* (℃)	*RH* (%)	*σ* (S/cm)	E_a (eV)	*Refs*
HOF-g	100	0	5.62×10^{-2}	0.23	[43]
HOIF-101	85	100	3.19×10^{-2}	0.25	This work
HOIF-103	85	100	3.18×10^{-2}	0.33	This work
GLYOA	60	95	3.03×10^{-2}	0.22	[44]
HOIF-203	85	100	2.60×10^{-2}	0.15	This work
HOIF-204	85	100	2.58×10^{-2}	0.23	This work
p-6PA-HPB	RT	95	2.50×10^{-2}	-	[45]
BALAOA	60	95	2.43×10^{-2}	0.4	[44]
HOL-DMSO	100	0	2.21×10^{-2}	0.4	[46]
CPOS-2	60	98	2.20×10^{-2}	0.61	[47]
HOIF-202	85	100	2.16×10^{-2}	0.25	This work
iHOF-16	100	98	2.11×10^{-2}	0.88	[48]
HOF-GS-11	30	95	1.80×10^{-2}	0.135	[49]
HOIF-102	85	100	1.69×10^{-2}	0.42	This work
TPMA-3F/MTBP	95	90	1.34×10^{-2}	1.01	[50]
HOF-TPB-A_3	23	98	1.34×10^{-2}	0.33	[51]
LEUOA	80	95	1.19×10^{-2}	0.86	[44]
HOIF-302	85	100	1.17×10^{-2}	0.18	This work
GC-4	85	98	1.11×10^{-2}	0.47	[52]
GC-3	85	98	1.09×10^{-2}	0.57	[52]
CPOS-1	60	98	1.00×10^{-2}	0.93	[47]
HOF-GS-10	30	95	7.50×10^{-3}	0.489	[49]
HDSD-1	80	98	7.50×10^{-3}	0.44	[53]
HOIF-201	85	100	6.64×10^{-3}	0.22	This work
HOF-SXU-1	160	0	6.32×10^{-3}	0.16	[54]
L-CH_3	80	98	4.20×10^{-3}	0.38	[55]
HOF-SZU-1	100	98	4.00×10^{-3}	0.31	[56]
HCOS-1	100	93	3.32×10^{-3}	0.32	[57]
GC-2	85	98	2.83×10^{-3}	0.46	[52]
BTC	85	100	1.80×10^{-3}	1.36	This work
NKM-HOF-10	85	98	1.70×10^{-3}	0.22	[58]
KUF-1a	80	90	1.53×10^{-3}	0.79	[59]
BDCDH	85	100	1.35×10^{-3}	1.27	This work
CB[6]·H_2SO_4	25	98	1.30×10^{-3}	0.31	[60]
iHOF-13	100	98	1.30×10^{-3}	0.15	[61]
NKM-HOF-11	85	98	1.29×10^{-3}	0.33	[58]
Im@HOF 2	80	98	1.20×10^{-3}	0.15	[62]
CB[6]·HCl	25	98	1.10×10^{-3}	0.39	[60]
RCCl-HCl	30	95	1.10×10^{-3}	0.35	[63]
CPOS-4	60	98	7.40×10^{-4}	0.82	[47]
HOIF-104	85	100	5.25×10^{-4}	0.93	This work
HOIF-301	85	100	4.56×10^{-4}	0.39	This work
HOF 2	50	98	4.32×10^{-4}	0.39	[62]
Im@HOF 1	60	98	4.12×10^{-4}	0.28	[62]
NKM-HOF-9	85	98	3.90×10^{-4}	0.56	[58]
CPOS-3	60	98	3.30×10^{-4}	0.62	[47]
L-Et	80	98	3.30×10^{-4}	0.39	[55]
HOF 1	70	98	3.11×10^{-4}	0.71	[62]
CFOS-2	50	98	2.20×10^{-4}	1.36	[64]
CFOS-1	50	98	1.90×10^{-4}	1.6	[64]
MA	85	100	1.89×10^{-4}	0.71	This work
CB[6]·HCO_2H	25	98	1.30×10^{-4}	0.56	[60]
BDC	85	100	1.07×10^{-4}	2.61	This work
HOF-H_3L	100	98	6.91×10^{-5}	0.68	[65]
RCC1-H_2SO_4	30	90	6.10×10^{-5}	0.1	[63]
L-H	80	98	6.00×10^{-5}	0.37	[55]
BDA	85	100	3.09×10^{-5}	0.46	This work
HOL-H_2O	60	0	7.07×10^{-6}	1.16	[46]
CC3	30	95	6.40×10^{-6}	0.11	[63]
GTUB5	75	90	4.20×10^{-6}	0.14	[66]
HOF-6a	27	97	3.40×10^{-6}	-	[67]
HOF-SXU-8	100	0	1.31×10^{-7}	0.51	[43]
HOL	60	0	7.08×10^{-9}	0.2	[46]

Table S8. The related hydrogen bond lengths and angles in HOIF-101.

D-H···A	d(D-H) (Å)	d(H···A) (Å)	d(D···A) (Å)	∠D-H···A (°)
O1W-H···O2[1]	0.85	2.02	2.83	161
O1W-Ha···O5[2]	0.85	1.92	2.76	174
N1-H1A···O2[2]	0.80	2.11	2.71	133
N1-H1B···O6[3]	0.90	1.89	2.74	156
N1-H1C···O1W[4]	1.05	1.81	2.75	147
N1-H1C···O4[1]	1.05	2.56	3.31	128
N3-Hb···O1[5]	0.89	1.95	2.80	157
N2-H2A···O3[6]	0.97	2.10	3.05	168
N2-H2B···O3[7]	0.84	2.20	2.97	153
N3-Hc···O1W[4]	0.94	1.98	2.89	161
N3-Hd···O4[4]	0.95	1.96	2.88	163
N3-Hd···O6[4]	0.95	2.55	3.04	112
N3-He···O5[8]	0.85	2.07	2.87	155

Symmetry codes:[1][1-x,1-y,-z]; [2][x,-1+y,z]; [3][1+x,-1+y,z]; [4][x,y,z]; [5][-1+x,y,z]; [6][-1+x,1+y,z]; [7][1-x,2-y,1-z]; [8][-x,2-y,-z].

Table S9. The related hydrogen bond lengths and angles in HOIF-301.

D-H···A	d(D-H) (Å)	d(H···A) (Å)	d(D···A) (Å)	∠D-H···A (°)
N1-H1NA···O2[1]	0.92	1.82	2.723	172
N1-H1NB···O1W[2]	0.90	1.98	2.83	157
N1-H1NB···O4[2]	0.90	2.56	2.88	101
N1-H1NC···O3W[3]	0.93	1.90	2.80	164
N2-H2NA···O2W[4]	0.93	1.86	2.79	178
N2-H2NB···O3[5]	0.91	1.90	2.78	162
N2-H2NC···O1[6]	0.92	2.22	2.89	131
N2-H2NC···O4[6]	0.92	2.26	3.02	140
O1W-H1WA···O6[3]	0.81	2.26	2.94	142
O1W-H1WB···O6[7]	0.80	2.01	2.80	175
O2W-H2WA···O5[7]	0.85	1.85	2.70	178
O2W-H2WB···O2[6]	0.83	1.97	2.78	172
O3W-H3WA···O2W[7]	0.86	1.93	2.79	174
O3W-H3WB···O3[5]	0.86	2.09	2.91	161

Symmetry codes:[1][1-x,-y,1-z]; [2][-x,-y,1-z]; [3][-x,1-y,1-z]; [4][1+x,y,z]; [5][x,1+y,z]; [6][1-x,1-y,2-z]; [7][x,y,z].

Table S10. Statistics on hydrogen bond length for HOIF-101 and HOIF-301.

Name	Minimum	Median	Maximum	Mean	Standard Deviation
HOIF-101	2.71	2.87	3.31	2.89	0.17
HOIF-301	2.70	2.80	3.02	2.83	0.09

Table S11. Comparison of hydrogen bond length in HOIF-101 and HOIF-301.

Name	Bond Length (H···A) (Å)		
	$-NH_3^+\cdots OH_2$	$-NH_3^+\cdots O_3^-S-$	$-SO_3^-\cdots H_2O$
HOIF-101	1.81	1.89	1.92
	-	2.56	2.02
	-	2.11	-
Average	1.81	2.19	1.97
HOIF-301	1.98	1.82	2.01
	1.90	2.56	2.26
	1.86	2.22	2.09
	-	2.26	1.85
	-	-	1.97
Average	1.91	2.22	2.04

Table S12. Hydrogen-bonding interaction energies in HOIF-101 and HOIF-301

**Name**	**Interaction Energy (kJ·mol^{-1})**		
	$-NH_3^+\cdots OH_2$	$-NH_3^+\cdots O_3^-S-$	$-SO_3^-\cdots H_2O$
HOIF-101	-398.79	-105.94	-298.59
HOIF-301	-43.72	-96.36	-21.51

4. Supplementary References

(1) Tauc, J., Grigorovici, R. & Vancu, A. Optical Properties and Electronic Structure of Amorphous Germanium. *Phys. Status Solidi B* **1966**, 15, 627-637.
(2) Bruker Apex3, Bruker AXS Inc., Madison, WI, 2019.
(3) Bruker SAINT: SAX Area-Detector Integration Program v8.40b, Bruker AXS Inc., Madison, WI, 2017.
(4) Krause, L., Herbst-Irmer, R., Sheldrick, G. M. & Stalke, D. Comparison of Silver and Molybdenum Microfocus X-Ray Sources for Single-Crystal Structure Determination. *J. Appl. Cryst.* **2015**, 48, 3-10.
(5) Brennan, S. & Cowan, P. L. A Suite of Programs for Calculating X-Ray Absorption, Reflection, and Diffraction Performance for a Variety of Materials at Arbitrary Wavelengths. *Rev. Sci. Instrum.* **1992**, 63, 850-853.
(6) Farrugia, L. WinGX and ORTEP for Windows: An Update. *J. Appl. Cryst.* **2012**, 45, 849-854.
(7) Sheldrick, G. A Short History of SHELX. *Acta Crystallogr. Sect. A.* **2008**, 64, 112-122.
(8) Kresse, G. & Furthmüller, J. Efficient Iterative Schemes for *ab initio* Total-Energy Calculations Using a Plane-Wave Basis Set. *Phys. Rev. B* **1996**, 54, 11169-11186.
(9) Bliichl, P. Projector Augmented-Wave Method. *Phys. Rev. B* **1994**, 50, 17953-17979.
(10) Kresse, G. & Joubert, D. From Ultrasoft Pseudopotentials to the Projector Augmented-Wave Method. *Phys. Rev. B* **1999**, 59, 1758-1775.
(11) Perdew, J. P., Burke, K. & Ernzerhof, M. Generalized Gradient Approximation Made Simple. *Phys. Rev. Lett.* **1996**, 77, 3865-3868.
(12) Klimeš, J., Bowler, D. R. & Michaelides, A. Chemical Accuracy for the van der Waals Density Functional. *J. Phys. Condens. Matter* **2009**, 22, 022201.
(13) Zhao, Y. & Truhlar, D. G. The M06 Suite of Density Functionals for Main Group Thermochemistry, Thermochemical Kinetics, Noncovalent Interactions, Excited States, and Transition Elements: Two New Functionals and Systematic Testing of Four M06-Class Functionals and 12 Other Functionals. *Theor. Chem. Acc.* **2008**, 120, 215-241.
(14) Weigend, F. & Ahlrichs, R. Balanced Basis Sets of Split Valence, Triple Zeta Valence and Quadruple Zeta Valence Quality for H to Rn: Design and Assessment of Accuracy. *Phys. Chem. Chem. Phys.* **2005**, 7, 3297-3305.
(15) Boys, S. F. & Bernardi, F. The Calculation of Small Molecular Interactions by the Differences of Separate Total Energies. Some Procedures with Reduced Errors. *Mol. Phys.* **1970**, 19, 553-566.
(16) Lu, T. & Chen, Q. in *Comprehensive Computational Chemistry (First Edition)* (eds Manuel Yáñez & Russell J. Boyd) 240-264 (Elsevier, 2024).
(17) Lu, T. & Chen, Q. Independent Gradient Model Based on Hirshfeld Partition: A New Method for Visual Study of Interactions in Chemical Systems. *J. Comput. Chem.* **2022**, 43, 539-555.
(18) Humphrey, W., Dalke, A. & Schulten, K. VMD: Visual Molecular Dynamics. *J. Mol. Graph.* **1996**, 14, 33-38.
(19) Duchamp, D. J. & Marsh, R. E. The Crystal Structure of Trimesic Acid (Benzene-1,3,5-Tricarboxylic Acid). *Acta Crystallogr. Sect. B.* **1969**, 25, 5-19.
(20) Bailey, M. & Brown, C. J. The Crystal Structure of Terephthalic Acid. *Acta Crystallogr.* **1967**, 22, 387-391.
(21) Czapik, A., Konowalska, H. & Gdaniec, M. p-Phenylenediamine and Its Dihydrate: Two-Dimensional Isomorphism and Mechanism of the Dehydration Process, and N-H...N and N-H...[pi] Interactions. *Acta Crystallogr. Sect. C* **2010**, 66, o128-o132.
(22) Betz, R., Klufers, P. & Mayer, P. m-Phenylenediamine. *Acta Crystallogr. Sect. E* **2008**, 64, o2501.
(23) Makuła, P., Pacia, M. & Macyk, W. How To Correctly Determine the Band Gap Energy of Modified Semiconductor Photocatalysts Based on UV–Vis Spectra. *J. Phys. Chem. Lett.* **2018**, 9, 6814-6817.
(24) Tan, K. *et al.* Competitive Adsorption of NH3 and H2O in Metal–Organic Framework Materials: MOF-74. *Chemistry of Materials* **2022**, 34, 7906-7915.
(25) Tan, K. *et al.* Water Reaction Mechanism in Metal Organic Frameworks with Coordinatively Unsaturated Metal Ions: MOF-74. *Chemistry of Materials* **2014**, 26, 6886-6895.
(26) Seki, T. *et al.* The Bending Mode of Water: A Powerful Probe for Hydrogen Bond Structure of Aqueous Systems. *The Journal of Physical Chemistry Letters* **2020**, 11, 8459-8469.
(27) Xie, F. *et al.* Complete separation of benzene-cyclohexene-cyclohexane mixtures via temperature-dependent molecular sieving by a flexible chain-like coordination polymer. *Nature Communications* **2024**, 15, 2240.
(28) Koupepidou, K. *et al.* One Atom Can Make All the Difference: Gas-Induced Phase Transformations in Bisimidazole-Linked Diamondoid Coordination Networks. *Journal of the American Chemical Society* **2023**, 145, 10197-10207.
(29) Ren, H.-M., Liu, B.-Y., Zuo, B.-T., Li, Z.-F. & Li, G. The Effect of Free Carboxylic Acid Groups on the Proton Conductivity of a Series of UiO-66-Ce(IV) Metal-Organic Frameworks. *Microporous Mesoporous Mater.* **2023**, 351, 112481.
(30) Phang, W. J. *et al.* Superprotonic Conductivity of a UiO-66 Framework Functionalized with Sulfonic Acid Groups by Facile Postsynthetic Oxidation. *Angew. Chem. Int. Ed.* **2015**, 54, 5142-5146.
(31) Yang, F. *et al.* Proton Conductivities in Functionalized UiO-66: Tuned Properties, Thermogravimetry Mass, and Molecular Simulation Analyses. *Cryst. Growth Des.* **2015**, 15, 5827-5833.
(32) Shigematsu, A., Yamada, T. & Kitagawa, H. Wide Control of Proton Conductivity in Porous Coordination Polymers. *Journal of the American Chemical Society* **2011**, 133, 2034-2036.
(33) Li, X.-M., Wang, Y., Mu, Y., Gao, J. & Zeng, L. Oriented Construction of Efficient Intrinsic Proton Transport Pathways in MOF-808. *J. Mater. Chem. A* **2022**, 10, 18592-18597.

(34) Sharma, A. *et al.* Superprotonic Conductivity of MOF-808 Achieved by Controlling the Binding Mode of Grafted Sulfamate. *Angew. Chem. Int. Ed.* **2021**, 60, 14334-14338.
(35) Luo, H.-B. *et al.* High Proton Conductivity Achieved by Encapsulation of Imidazole Molecules into Proton-Conducting MOF-808. *ACS Appl. Mater. Interfaces* **2019**, 11, 9164-9171.
(36) Luo, H.-B. *et al.* Proton Conductance of a Superior Water-Stable Metal–Organic Framework and Its Composite Membrane with Poly(vinylidene fluoride). *Inorg. Chem.* **2017**, 56, 4169-4175.
(37) Mallick, A., Kundu, T. & Banerjee, R. Correlation between Coordinated Water Content and Proton Conductivity in Ca–BTC-Based Metal–Organic Frameworks. *Chem. Comm.* **2012**, 48, 8829-8831.
(38) Liu, Y. *et al.* Polyoxometalate-Functionalized Metal–Organic Frameworks with Improved Water Retention and Uniform Proton-Conducting Pathways in Three Orthogonal Directions. *Chem. Comm.* **2014**, 50, 10023-10026.
(39) Jeong, N. C., Samanta, B., Lee, C. Y., Farha, O. K. & Hupp, J. T. Coordination-Chemistry Control of Proton Conductivity in the Iconic Metal–Organic Framework Material HKUST-1. *J. Am. Chem. Soc.* **2012**, 134, 51-54.
(40) Wu, D.-Q. *et al.* Slow Magnetic Relaxation and Proton Conduction in a Highly Hydrogen-Bonded Dinuclear Holmium(III) Complex. *J. Mol. Struct.* **2024**, 1306, 137874.
(41) Phang, W. J. *et al.* pH-Dependent Proton Conducting Behavior in a Metal–Organic Framework Material. *Angew. Chem.* **2014**, 126, 8523-8527.
(42) Hwang, S., Lee, E. J., Song, D. & Jeong, N. C. High Proton Mobility with High Directionality in Isolated Channels of MOF-74. *ACS Applied Materials & Interfaces* **2018**, 10, 35354-35360.
(43) Yang, F.-F., Wang, X.-L., Tian, J., Yin, Y. & Liang, L. Vitrification-Enabled Enhancement of Proton Conductivity in Hydrogen-Bonded Organic Frameworks. *Nat. Commun.* **2024**, 15, 3930.
(44) Pramanik, T. *et al.* Enhanced Proton Conductivity in Amino Acid Based Self-Assembled Non-Porous Hydrogen-Bonded Organic Frameworks. *Chem. Comm.* **2022**, 58, 5972-5975.
(45) Jiménez-García, L. *et al.* Phosphonated Hexaphenylbenzene: A Crystalline Proton Conductor. *Angew. Chem. Int. Ed.* **2009**, 48, 9951-9953.
(46) Wang, X.-L. *et al.* Boosting Proton Conductivity in Hydrogen-Bonded Organic Layers by Modulating Embedded Guest Molecules. *Cryst. Growth Des.* **2023**, 23, 6221-6227.
(47) Xing, G., Yan, T., Das, S., Ben, T. & Qiu, S. Synthesis of Crystalline Porous Organic Salts with High Proton Conductivity. *Angew. Chem. Int. Ed.* **2018**, 57, 5345-5349.
(48) Cao, X.-J., Cao, L.-H., Bai, X.-T., Hou, X.-Y. & Li, H.-Y. An Ultra-Robust and 3D Proton Transport Pathways iHOF with Single-Crystal Superprotonic Conductivity Around 0.4 $S.cm^{-1}$. *Adv. Funct. Mater.* **2024**, 34, 2409359.
(49) Karmakar, A. *et al.* Hydrogen-Bonded Organic Frameworks (HOFs): A New Class of Porous Crystalline Proton-Conducting Materials. *Angew. Chem. Int. Ed.* **2016**, 55, 10667-10671.
(50) Ami, T., Oka, K., Kitajima, S. & Tohnai, N. Highly Fluorinated Nanospace in Porous Organic Salts with High Water Stability/Capability and Proton Conductivity. *Angew. Chem. Int. Ed.* **2024**, 63, e202407484.
(51) Wang, Z. *et al.* Anisotropic Superprotonic Conduction in a Layered Single-Component Hydrogen-Bonded Organic Framework with Multiple In-Plane Open Channels. *Adv. Mater.* **2024**, 36, 2409202.
(52) Wu, W.-W. *et al.* Controllable Synthesis and Ultrahigh Anisotropic Single-Crystal Proton Conduction of a Hydrogen-Bonded Organic Framework. *Chem. Mater.* **2023**, 35, 6549-6556.
(53) Yang, Q. *et al.* Enhanced Stability and Ultrahigh Proton Conductivity of Hydrogen-Bonded Organic Frameworks. *J. Mater. Chem. C* **2023**, 11, 12206-12212.
(54) Wang, X.-L., Liang, L., Dou, S. & Zhang, X.-M. Two-in-One Tecton Strategy to Construct Single Crystalline Hydrogen-Bonded Organic Framework with High Proton Conductivity above 100 °C. *Sci. China Chem.* **2023**, 66, 2563-2568.
(55) Sun, Y. *et al.* Bio-Inspired Synthetic Hydrogen-Bonded Organic Frameworks for Efficient Proton Conduction. *Adv. Mater.* **2023**, 35, 2208625.
(56) Du, J. *et al.* A Crystalline Imidazole-Carboxylic Acid-Based Hydrogen-Bonded Organic Framework with High Proton Conductivity. *J. Solid State Chem.* **2025**, 342, 125109.
(57) Yang, Y. *et al.* Enhanced Proton Conduction of Crystalline Organic Salt Hybrid Membranes and the Performance of Fuel Cells. *Mater. Chem. Front.* **2022**, 6, 3402-3408.
(58) Xu, L. *et al.* Rational Tuning the Proton Conductivity and Stability of Hydrogen-Bonded Organic Frameworks. *Inorg. Chem.* **2024**, 63, 17747-17754.
(59) Kang, D. W. *et al.* A Hydrogen-Bonded Organic Framework (HOF) with Type IV NH_3 Adsorption Behavior. *Angew. Chem. Int. Ed.* **2019**, 58, 16152-16155.
(60) Yoon, M. *et al.* High and Highly Anisotropic Proton Conductivity in Organic Molecular Porous Materials. *Angew. Chem. Int. Ed.* **2011**, 50, 7870-7873.
(61) Bai, X.-T., Cao, L.-H., Zhao, F. & Li, S.-H. Arylsulfonate Ionic Hydrogen-Bonded Organic Frameworks Enable Highly Stable and Superprotonic Conductivity for Enhancing Direct Methanol Fuel Cells. *ACS Mater. Lett.* **2024**, 6, 3351-3357.
(62) Hao, B.-B., Wang, X.-X., Zhang, C.-X. & Wang, Q. Two Hydrogen-Bonded Organic Frameworks with Imidazole Encapsulation: Synthesis and Proton Conductivity. *Cryst. Growth Des.* **2021**, 21, 3908-3915.
(63) Liu, M. *et al.* Three-Dimensional Protonic Conductivity in Porous Organic Cage Solids. *Nat. Commun.* **2016**, 7, 12750.
(64) Wang, C. *et al.* Electron and Proton Conducting Framework Prganic Salt Single Crystals. *J. Solid State Chem.* **2022**, 308, 122903.
(65) Sun, Z.-B. *et al.* A Path to Improve Proton Conductivity: From a 3D Hydrogen-Bonded Organic Framework to a 3D

Copper-Organic Framework. *New J. Chem.* **2019**, 43, 10637-10644.
(66) Tholen, P. *et al.* Semiconductive Microporous Hydrogen-Bonded Organophosphonic Acid Frameworks. *Nat. Commun.* **2020**, 11, 3180.
(67) Yang, W. *et al.* Microporous Diaminotriazine-Decorated Porphyrin-Based Hydrogen-Bonded Organic Framework: Permanent Porosity and Proton Conduction. *Cryst. Growth Des.* **2016**, 16, 5831-5835.
